# Integrative genomics and zero‐shot deep learning–based phenotyping reveal the genetic architecture of seed traits in mungbean (*Vigna radiata* L.)

**DOI:** 10.1002/tpg2.70294

**Published:** 2026-08-19

**Authors:** Venkata Naresh Boddepalli, Talukder Zaki Jubery, Steven B. Cannon, Andrew Farmer, Somak Dutta, Baskar Ganapathysubramanian, Arti Singh

**Affiliations:** ^1^ Department of Agronomy Iowa State University Ames Iowa USA; ^2^ Department of Mechanical Engineering Iowa State University Ames Iowa USA; ^3^ USDA‐ARS Corn Insects, and Crop Genetics Research Unit Ames Iowa USA; ^4^ National Center for Genome Resources Santa Fe New Mexico USA; ^5^ Department of Statistics Iowa State University Ames Iowa USA

## Abstract

Improving seed size and weight is a major breeding goal in mungbean (*Vigna radiata* (L.) R. Wilczek). Improved genomic resources and precision phenotyping may enable more efficient selection for seed trait improvement. In this study, we integrated a deep learning–based segment anything model phenotyping pipeline with genome‐wide association studies (GWAS), comparative mapping, and genomic prediction (GP) to dissect the genetic architecture of seed size, shape, and weight traits in the Iowa mungbean diversity panel. The zero‐shot segmentation approach reliably captured seed size traits, which exhibited high heritability (*H* ^2^ > 0.90) and strong correlation with seed weight (*r* > 0.91). A multi‐model GWAS identified 82 unique single nucleotide polymorphisms (SNPs) across all seven traits, of which 13 were major pleiotropic SNPs governing multiple seed dimensions, including high‐confidence regions on chromosomes 1, 4, and 6 that explained over 20% of the phenotypic variance. Within these SNP regions, comparative mapping highlighted candidate genes including an ABC transporter (*Virad01G0084400*) and two colocated candidates, NPGR1 (*Virad06G0255600*) and a RING‐type E3 ubiquitin‐protein ligase (*Virad06G0255800*), presented as hypothesis‐generating candidates for seed size regulation. GP using genomic best linear unbiased prediction (gBLUP) produced moderate to high accuracies for seed size and weight traits (*r* = 0.76–0.84). Incorporating significant GWAS SNPs (gBLUP + SNPs) yielded slight improvements, suggesting the standard gBLUP model is sufficiently robust for selection. Collectively, this study provides the most comprehensive genomic dissection of seed size and weight traits in mungbean to date, providing candidate loci and genomic prediction models that can accelerate genetic improvement for seed yield and quality.

## Core Ideas

- Zero‐shot deep learning (segment anything model [SAM]) enables highly precise, automated extraction of mungbean seed traits with minimal training.
- Extracted seed dimensions and weight display high heritability and phenotypic variance across the mungbean diversity panel.
- Genome‐wide association studies (GWAS) revealed 13 pleiotropic single nucleotide polymorphisms (SNPs) and highlighted conserved candidate genes for seed development.
- Genomic prediction models achieved high accuracies for seed size and weight, establishing a robust framework for legume pre‐breeding.

## Plain Language Summary

Considering the growing global demand for mungbeans due to their rich nutritional profile, improving seed yield is crucial. Seed size and weight are major contributors to yield, and understanding their genetic architecture is essential to accelerate cultivar development. To precisely phenotype these size‐related traits, deep learning‐based high‐throughput phenotyping is needed to ensure accuracy and reduce labor costs. This research aimed to integrate high‐throughput image‐based phenotyping with genome‐wide association and prediction analyses to better understand the genetic architecture of seed traits. We found that this integrated approach successfully extracted seed dimensional traits that strongly correlate with manually measured seed weight. We identified several candidate genes associated with seed size and weight traits.

**Abbreviations**
BLINK: Bayesian‐information and linkage‐disequilibrium iteratively nested keyway
BLUP: best linear unbiased prediction
CI: coincidence index
FarmCPU: fixed and random model circulating probability unification
GAPIT: genomic association and prediction integrated tool
GBLUP: genomic best linear unbiased prediction
GEBV: genomic estimated breeding values
GP: genomic prediction
GPA
: genomic prediction accuracy
GWAS: genome‐wide association studies
HSV: hue, saturation, and value
IMD: Iowa mungbean diversity
LD: linkage disequilibrium
MLM: mixed linear model
MTA: marker–trait association
PCA: principal component analysis
QTL: quantitative trait locus
ROI: regions of Interest
SAM: segment anything model
SNP: single nucleotide polymorphism
SVEN: selection of variables with embedded screening using Bayesian methods
TASSEL: trait analysis by association, evolution, and linkage

## 1 INTRODUCTION

Mungbean (*Vigna radiata* (L.) R. Wilczek) is a short‐duration, nitrogen‐fixing grain legume known for its high nutritional profile, grown widely in Asia, Africa, Australia, and some parts of North America (Boddepalli & Singh, 2026; Christian et al., 2023; Lin et al., 2025; J. Liu et al., 2022; Nair & Schreinemachers, 2020; Sandhu & Singh, 2021; Somta et al., 2022; Wilbur et al., 2025). Despite its nutritional and agronomic advantages, mungbean yields remain low and unstable, averaging 0.73 t/ha across production environments (J. Liu et al., 2022; Nair et al., 2022; Ong et al., 2023; Wilbur et al., 2025). Among the yield components, seed size traits, which directly contribute to seed weight, are important as they can also affect farmer and consumer preferences (Akinmade et al., 2025; Christian et al., 2023; Kassa et al., 2022; J. Liu et al., 2022).

Seed size and weight are the major yield‐contributing traits in mungbean, like in other grain legumes, controlled by many loci and subject to strong domestication and improvement selection (Bohra et al., 2022; Padhy et al., 2025; Reddappa et al., 2025; Sen Gupta et al., 2023; Tayade et al., 2023; Verma et al., 2022; H. Zhang et al., 2022). In mungbean, seed size traits, including seed length (SL), width (SW), and 100‐seed weight (SWT), play a crucial role in determining seedling vigor and, ultimately, seed yield per plant (X. Han et al., 2024; Kadam et al., 2023). Seed size also strongly influences milling performance, affecting dehulling efficiency, flour fineness, and functional properties, thereby enhancing the seed market value (Sakhare et al.,^2026^; Skylas et al., 2025; Yu et al., 2023).

Despite its agronomic and economic importance, the genetic basis of seed size variation in mungbean is poorly characterized. Understanding the genetic architecture underlying seed size traits is therefore essential for accelerating genetic improvement in mungbean breeding programs. Prior genetic studies in mungbean, largely based on biparental quantitative trait loci (QTLs) mapping, have identified genomic regions associated with seed weight; however, these studies have been constrained by small population sizes, limited recombination, and reliance on labor‐intensive manual measurements (X. Han et al., 2024; Humphry et al., 2005; Reddappa et al., 2025). Consequently, candidate genes associated with these size‐related traits remain poorly characterized (Ahmed et al., 2024; S. Kim et al., 2015; Reddappa et al., 2025).

Genome‐wide association studies (GWAS) offer higher mapping resolution over traditional linkage mapping by exploring historical recombination events in diverse germplasm panels and widely used in legumes such as cowpea (Akinmade et al., 2025; L. Han et al., 2025; Kpoviessi et al., 2022; Lay et al., 2024), common bean (Giordani et al., 2022; Izquierdo et al., 2023; Ugwuanyi et al., 2022), and soybean (Bhat et al., 2025; Cao et al., 2022; J. Kim et al., 2025; Shao et al., 2022) to identify loci associated with seed size and weight. However, comprehensive GWAS focused on seed size and weight in mungbean remain limited (J. Liu et al., 2022; Manjunatha et al., 2023), partly due to the challenges of obtaining high‐quality, high‐throughput phenotypic data.

Traditional manual seed size measurements are time‐consuming, prone to human error, and difficult to scale (Morales et al., 2024; Xiao et al., 2022). Recent advances in computer vision have enabled automated phenotyping of grain traits across several species (Singh et al., 2021), including soybean (J. Kim et al., 2025), cowpea (Akinmade et al., 2025; Lay et al., 2024), common bean (Burridge et al., 2017; Giordani et al., 2022), and rice (Guo et al., 2018; Rebolledo et al., 2016; Satrio et al., 2025), demonstrating strong concordance with manual measurements. However, while traditional computer vision relies heavily on global color thresholding (e.g., hue, saturation, and value [HSV] or RGB color spaces) to segment objects, these pipelines are extremely weak when applied to highly diverse germplasm (K. Zhao, 2024; L. Zhao et al., 2022). In mungbean specifically, the high prevalence of complex seed coat phenotypes, such as severe mottling, dark pigmentation, and shiny seed coat, frequently confounds color‐based segmentation. These optical complexities trick traditional algorithms into misclassifying seed pixels as background, resulting in fragmented or merged seed masks (Bente et al., 2026). Unlike traditional methods requiring extensive training data, the segment anything model (SAM) represents a flexible deep learning framework capable of zero‐shot instance segmentation across diverse imaging contexts, making it suitable for seed phenotyping workflows that require minimal training (Archit et al., 2025; Balasundaram et al., 2024; Kirillov et al., 2023; J. Li et al., 2025; Morales et al., 2024; Nichols et al., 2024; Osco et al., 2023; Swartz et al., 2023; R. Wang et al., 2025; Zu et al., 2025). However, the potential of this zero‐shot deep learning framework remains unexplored in mungbean. To our knowledge, this is the first study to deploy SAM for high‐throughput phenotyping of mungbean seeds. To date, no image analysis‐based studies on seed size traits have been conducted in mungbean, apart from a few reports that relied solely on manual phenotyping (J. Liu et al., 2022).

In this study, we aimed to integrate a high‐throughput, deep learning‐based image analysis pipeline using the SAM (Kirillov et al., 2023) with genomic approaches to dissect the genetic architecture underlying seed size, shape, and weight traits in the Iowa mungbean diversity (IMD) panel. The objectives of this study were to (i) extract seed size and shape traits using a zero‐shot SAM‐based image analysis, (ii) identify genomic regions associated with these traits through GWAS and comparative mapping, and (iii) perform genomic prediction (GP) analysis to assess its utility for improving seed size‐related breeding decisions.

**Core Ideas**

- Zero‐shot deep learning (segment anything model [SAM]) enables highly precise, automated extraction of mungbean seed traits with minimal training.
- Extracted seed dimensions and weight display high heritability and phenotypic variance across the mungbean diversity panel.
- Genome‐wide association studies (GWAS) revealed 13 pleiotropic single nucleotide polymorphisms (SNPs) and highlighted conserved candidate genes for seed development.
- Genomic prediction models achieved high accuracies for seed size and weight, establishing a robust framework for legume pre‐breeding.

## 2 MATERIALS AND METHODS

### 2.1 Diversity panel and experimental design

The IMD panel evaluated in this study comprised 372 genotypes: 363 accessions, four breeding lines from the World Vegetable Center, and five commercially released cultivars (Boddepalli et al., 2026a; Chiteri et al., 2023; Sandhu & Singh, 2021). Of these, 368 genotypes with complete genotypic and phenotypic data were retained for downstream genomic association and prediction analyses; the remaining four were excluded due to missing sequencing data. Field experiments with all the above 368 genotypes were conducted across three environments: Agricultural Engineering and Agronomy (AEA) Research Farm (42°00′58.5″ N, 93°46′16.7″ W) and Burkey farm (42° 00′ 36.7″ N 93°47′17.6″ W), in 2022, and Bruner farm (42°00′44.9″ N 93°43′46.8″ W), in Iowa, in 2024. All three environments represent unique site‐year combinations; The IMD panel was planted on June 2, 2022, at AEA and Burkey farms, and on June 2, 2024, at Bruner farm, in a randomized complete block design with two blocks per environment. Each genotype was planted in a three‐row plot measuring 5 feet in length, with 15‐in. row spacing and 2‐in. plant spacing, and 100 seeds per plot. Standard agronomic management practices were followed throughout the experimental period, and all trials were strictly rainfed. Between the two study years, the growing season (June through September) average temperature was nearly identical across locations (22.0°C in both years), yielding highly comparable cumulative growing degree days (base temperature = 10°C, following McMaster [1997] and Boddepalli and Singh [2026]) (1463.0°C·day in 2022 vs. 1463.9°C·day in 2024); however, the 2022 environment received moderately higher total precipitation (377.7 mm) compared to the 2024 environment (332.5 mm).

### 2.2 Image acquisition

**FIGURE 1:**
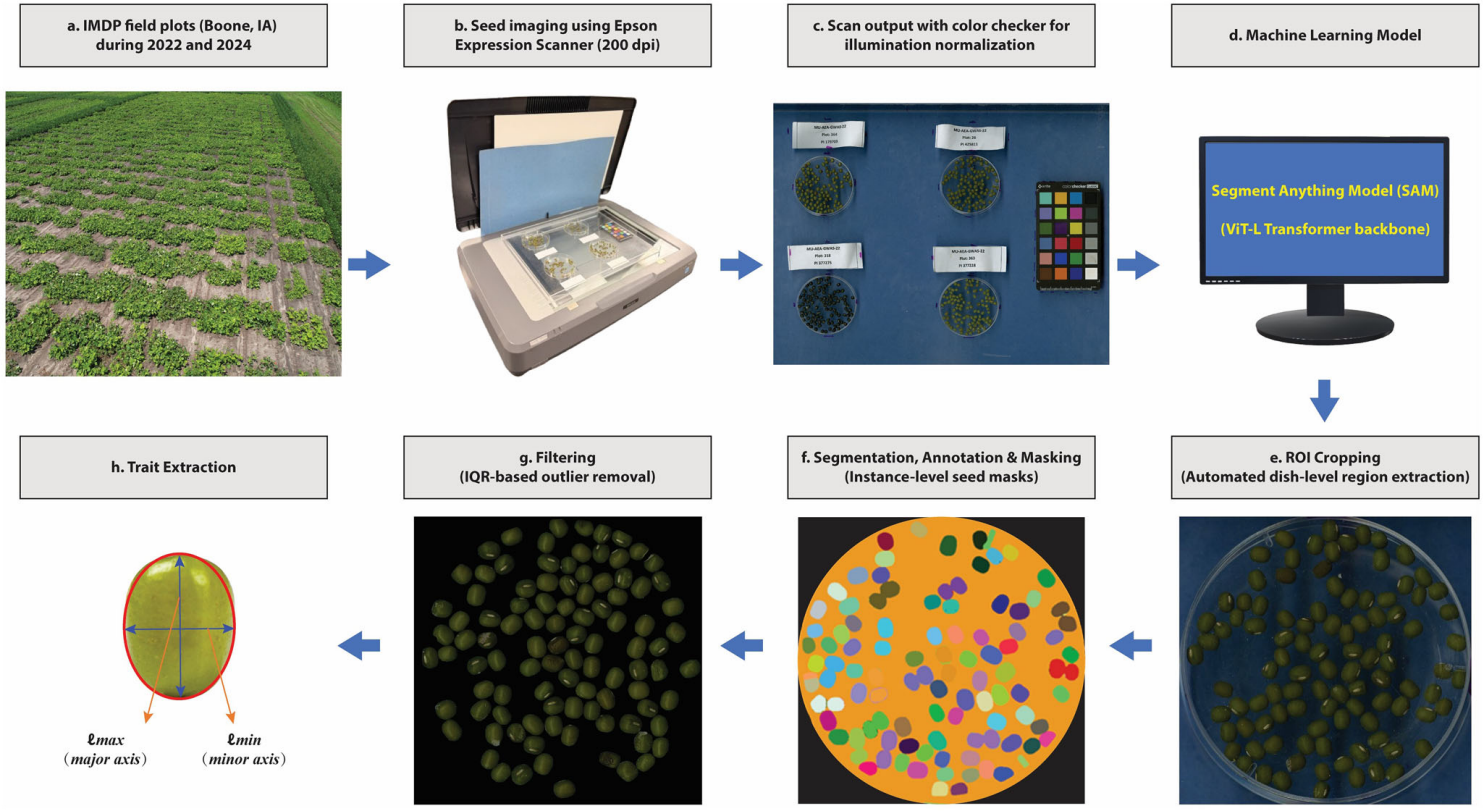
An image‐based mungbean seed phenotyping pipeline. (a) IMDP field plots (Boone, IA; 2022–2024). (b) An Epson expression flatbed scanner for seed imaging. (c) Scanned image output. (d) A machine learning workflow based on the segment anything model (SAM) for seed instance detection. (e–g) Dish‐level cropping, seed masking, and filtering tasks. Each segmented seed mask is displayed in a unique, randomly assigned pseudo‐color for visualization purposes only, to distinguish individual seed instances within a dish. (h) Final trait extraction derived geometric features (e.g., maximum/major axis length, minimum/minor axis length).

The mature pods were harvested from the top three nodes of the five randomly selected plants in each plot when they reached the R6 stage (90% maturity). This standardized upper‐canopy sampling was required to maintain methodological continuity with our prior pod phenotyping study (Boddepalliet al., 2026a), from which these specific seeds were threshed; however, we acknowledge this approach may not capture whole‐plant positional variance in seed size. A random sample of 20 pods was manually threshed, and 100 seeds were selected for scanning. These randomly selected 100 seeds were placed in a transparent 90 mm Petri dish. Four similarly filled Petri plates with seeds of four different genotypes were placed on a large rectangular clear fiber plate, as shown in Figure 1c. Additionally, an X‐Rite Color Checker card was used for normalization, and a white tag with metadata for each genotype was placed in a designated location on the fiber plate. The entire fiber plate was then covered with a thick, blue sheet during imaging with an Epson Expression 11000XL flatbed scanner (Epson America, Inc.). Image resolution was 4299 × 3035 pixels at 200 dpi, saved as 24‐bit JPEGs.

### 2.3 Ground truth measurement and imaging setup

A physical validation experiment was conducted using a subset of 240 seeds to assess the accuracy of the digital trait extraction. These comprised 60 seeds from four distinct genotypes (green, black, mottled, and yellow) selected to capture the phenotypic diversity of mungbean seed coat colors. To validate the zero‐shot SAM pipeline, we employed a color‐stratified sampling design. We selected four accessions representing the panels' distinct visual classes (green, yellow, black, and mottled). Since seed coat color and texture are the primary causes of segmentation failure in traditional models, demonstrating geometric accuracy across these extremes could be sufficient to validate the model for the broader 372‐genotype panel. Seeds were manually arranged in a 6 × 10 spatial grid on a transparent Petri plate, with an X‐Rite ColorChecker Passport included. This strict grid arrangement ensured a one‐to‐one traceability between the physical seed and its corresponding digital mask. High‐resolution images of each batch were captured under controlled, diffuse lighting to minimize shadows and specular reflection. Immediately after imaging, the length and width of each seed (in millimeters) were measured using digital Vernier calipers (Table S1). This data served as ground truth for validating the data derived from the SAM‐based model.

### 2.4 Manual data collection on SWT (g)

After image acquisition, the same set of scanned 100 seeds was immediately weighed to determine the SWT for each genotype. Measurements were taken using a PRSeries Precision balance (Model PR822N/E; precision ± 0.01 g). The final weights were recorded in grams for further analyses.

### 2.5 Image analysis and trait extraction

The metadata tags were located using hardcoded coordinates relative to the dish's known position. Extracted tags were binarized, and text regions were isolated using *connected components and bounding rectangles*. An optical character recognition (Tesseract v5.3.0) was configured to restrict the character set. Numeric strings and PI‐prefixed labels were parsed using regular expressions. Using a graphical user interface, regions of interest (ROIs) for four Petri dishes were drawn on a reference image and saved in a cropping‐coordinates.json file. These ROIs were batch‐applied to extract dish‐level crops, each tagged with its associated metadata. Seed segmentation was performed using the SAM with the ViT‐L backbone (checkpoint: sam_vit_l_0b3195.pth). Each segmented mask was assigned a unique pseudo‐color for visualization; these colors carry no biological meaning and are used solely to distinguish individual seed instances. Segmentation used SAM's automatic mask generation mode with default parameters (points_per_side = 32, points_per_batch = 64, pred_iou_thresh = 0.88, stability_score_thresh = 0.95, stability_score_offset = 1.0, box_nms_thresh = 0.7, crop_n_layers = 0, and min_mask_region_area = 0). Unlike traditional methods, which rely on specific color channels, the SAM architecture uses edge‐ and texture‐based features (Kirillov et al., 2023; J. Li et al., 2025; Osco et al., 2023), enabling robust segmentation of seeds across diverse coat colors in this diversity panel. The use of glass slides occasionally introduced specular reflections and lighting artifacts. However, these artifacts typically produced segmented masks that were either significantly smaller or significantly larger than the true seeds. To extract quantitative phenotypic traits (Table 1), pixel dimensions were converted to physical measurements using conversion factors of 1 pixel = 0.127 mm for axis lengths (SL, SW, and seed perimeter [SPM]) and 1‐pixel^2^ = 0.0161 mm^2^ for seed area (SA), consistent with the 200‐dpi scanning resolution (25.4 mm/in. ÷ 200 dpi = 0.127 mm/pixel). This conversion factor was established based on the optical resolution and empirically verified using a physical reference ruler included alongside the color chart in the imaging field of view. The same conversion factor was applied uniformly across all imaging batches and environments. To ensure data quality, we applied an area‐based filtering using an interquartile range (IQR) method: *ℓ* = *Q*_1_ − 1.5 IQR, and = *Q*_3_ + 1.5 IQR; where IQR = Q_3_ − Q_1_ and only masks within the bounds [*ℓ*,*u*] were retained. This filter removed an average of 17.4% of masks per image (SD = 2.6%, range 9.0%–23.8%); removal rate was weakly associated with seed size (Pearson *r* = 0.115, Spearman *ρ* = 0.124), indicating the filter primarily excluded outlier masks rather than shifting the central size distribution. From the filtered masks, we computed the following parameters: major axis length (SL; *ℓ*_max_), minor axis length (SW; *ℓ*_min_), SA, seed aspect ratio (SAR), SPM, and seed circularity (SCR).

**TABLE 1:** Description of the seed size and shape traits and their units used in this study.

Trait	Description	Units/formula
Seed length (*ℓ*_max_) (SL)	Major axis length of the fitted ellipse or the longest dimension of the seed mask	mm
Seed width (*ℓ*_min_) (SW)	Minor axis length of the fitted ellipse or shortest dimension perpendicular to *ℓ*_max_	mm
Seed area (SA)	Total number of pixels within the seed mask	mm^2^
Seed aspect ratio (SAR)	Elongation of the seed, computed as length divided by width	*ℓ*_max_/*ℓ*_m__i__n_
Seed perimeter (SPM)	Length of the boundary of the seed mask	mm
Seed circularity (SCR)	Compactness of the seed relative to a circle	4∏*(seed area/perimeter^2^)
100‐seed weight (SWT)	Weight of 100 seeds	g

Traits were compiled into a Pandas DataFrame and saved as CSV files for downstream analysis. The phenotyping pipeline was developed in Python 3.10 using PyTorch 1.13 for SAM inference. Preprocessing and geometric measurements were performed using OpenCV, Albumentations, and scikit‐image, and the resulted trait data were exported as CSV files for downstream analyses. These computations were executed on NVIDIA A100 GPUs to support high‐throughput image processing. To ensure full computational reproducibility, the complete analysis pipeline and configuration files are version‐controlled and publicly accessible at https://github.com/baskargroup/MungSeedAI/tree/master.

### 2.6 Traditional color‐based image segmentation (baseline method)

**FIGURE 2:**
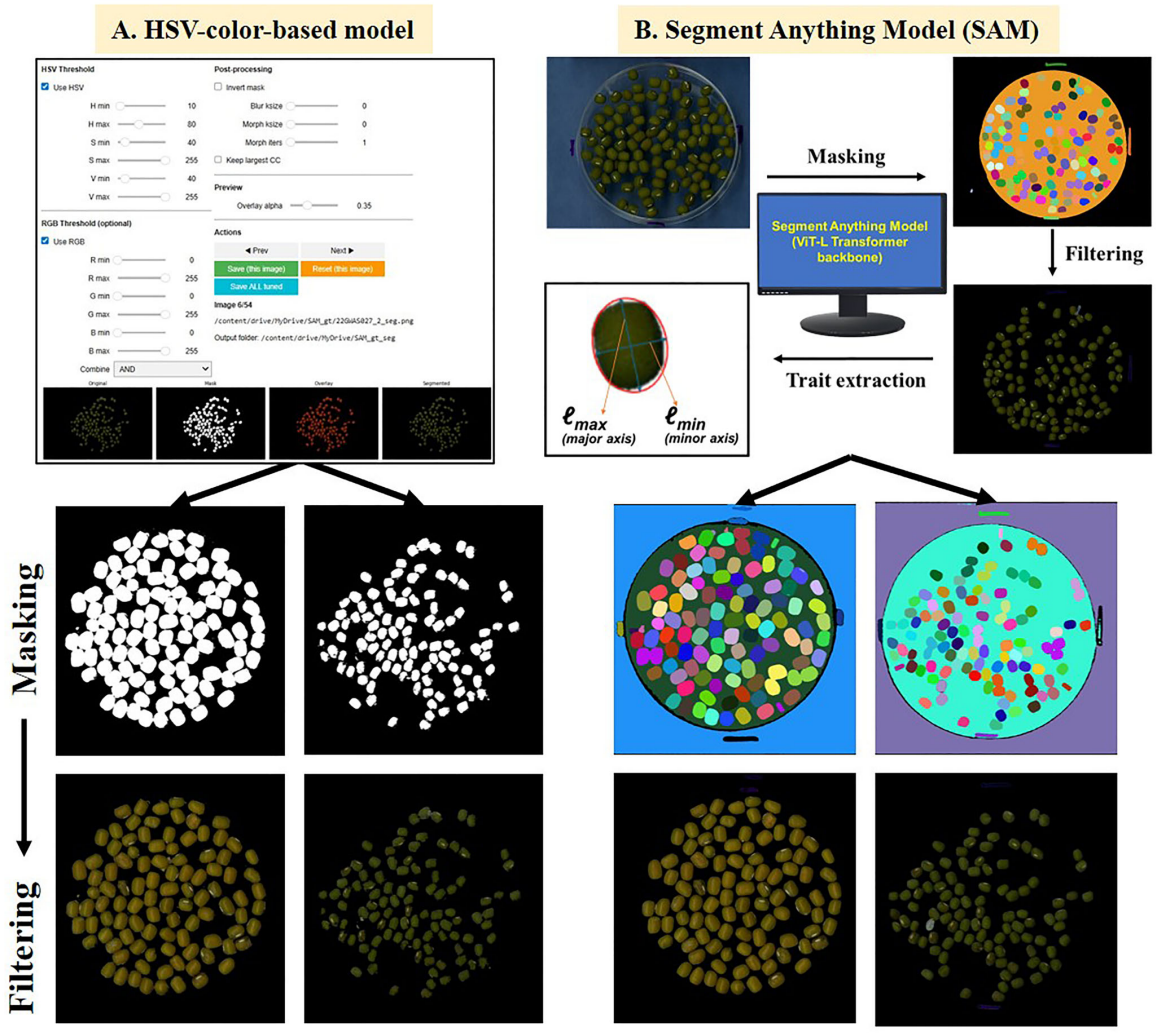
Comparison of seed masking and filtering performance between the traditional hue, saturation, and value (HSV) color‐based model and the segment anything model (SAM). (A) The HSV color‐based model interface (top), its corresponding initial seed masks (middle), and the final filtered output (bottom). (B) The SAM‐based deep learning pipeline (top) demonstrates robust, instance‐level seed masking (middle) and the final filtered output used for precise trait extraction (bottom). Arbitrary pseudo‐colors are applied by the model solely to visually distinguish individual seed instances, while an interquartile range (IQR) filter is applied to automatically discard geometric outliers (e.g., touching or partial seeds) before trait extraction.

To serve as a baseline for the deep learning approach, a traditional color thresholding pipeline was developed in Python using the OpenCV library. Raw images were converted from the standard BGR color space to HSV, which is generally more robust to minor lighting variations (Figure 2). Seed segmentation was performed by defining global upper and lower threshold bounds for the H, S, and V channels to isolate seed coat pixels from the blue background. Because mungbean genotypes exhibit significant variations in seed coat color and variegation, these thresholds required manual, interactive tuning for different batches. Following thresholding, morphological operations (e.g., morphological opening and closing with an elliptical structuring element) were applied to the binary masks to reduce background noise and fill small interior gaps caused by surface reflections. Finally, connected component analysis was utilized to identify and extract the individual seed boundaries for trait measurement.

### 2.7 Phenotypic data analysis

The seed dimensional trait data generated from the image analysis model were analyzed using R software (R Core Team, 2021). Data quality control was performed for each trait prior to analysis. Outliers were identified and removed using an iterative restricted model approach based on studentized residuals (Bonferroni‐corrected *p* < 0.05) using the *outlierTest* function within the *car* package (Fox & Weisberg, 2019). Following rigorous, iterative outlier removal, residual distributions were deemed adequate to satisfy the linear mixed model assumptions without the need for mathematical transformations. Consequently, all traits were modeled on their original phenotypic scales to ensure direct comparability of estimated effects in downstream analyses.

To calculate the best linear unbiased estimations (BLUEs) (Table S2) for each genotype using the lme4 package (Bates et al., 2015). A mixed linear model (MLM) was fitted where genotype was treated as a fixed effect, while environment, genotype × environment interaction, and block (nested within environment) were treated as random effects:

$$y_{ijk}=\mu +G_{i}+E_{j}+{B_{k}}_{\left(j\right)}+{\left(G\times E\right)}_{ij}+\varepsilon_{ijk}$$

Where *y_ijk_* is an observed phenotypic value, *μ* is the overall mean, *G_i_* is the fixed effect of the *i*th genotype, *E_j_* is the random effect of the *j*th environment (assumed to be ∼*N*(0, $\sigma_{E}^{2}$)), *B_k_*_(_ *_j_*_)_ is the random effect of the *k*th block nested within the *j*th environment (assumed to be ∼*N*(0, $\sigma_{B}^{2}$)), (*G* × *E*)*_ij_* is the random interaction effect of the *i*th genotype and *j*th environment (assumed to be ∼ *N*(0, $\sigma_{GE}^{2}$)), and *ε_ijk_* is the residual error. The BLUEs (adjusted means) were extracted using the *emmeans* package (Lenth & Piaskowski, 2017). This fixed‐effects specification was chosen to obtain BLUEs rather than best linear unbiased predictions (BLUPs), since the phenotypic values served as direct input for GWAS, and BLUEs avoid the double‐shrinkage bias that BLUPs can introduce in two‐stage association analyses (Piepho et al., 2003).

Variance components were estimated by fitting a fully random effects model with the same terms as above, but with genotype treated as a random effect. Broad‐sense heritability was assessed using the estimated variance components as:

$$\;H^{2}=\sigma_{g}^{2}\;/\left(\sigma_{g}^{2}+\sigma_{ge}^{2}/n_{\mathrm{env}}+\sigma_{e}^{2}/\left(n_{\mathrm{env}}\times n_{\mathrm{rep}}\right)\right)$$

where $\sigma_{g}^{2}$ is the genotypic variance, $\sigma_{ge}^{2}$ is the genotype × environment interaction variance, $\sigma_{e}^{2}$ is the residual variance, $n_{\mathrm{env}}$ is the number of environments, and $n_{\mathrm{rep}}$ is the harmonic mean of the number of replications per genotype.

Principal component analysis (PCA) was performed on the phenotypic BLUEs for seed size, weight, and shape traits using the FactoMineR package (v2.11) (Husson et al., 2016) in R software. Prior to the analysis, the data were standardized to unit variance to account for trait scales. Trait contributions to the principal components were calculated, followed by the generation of biplots to visualize the relationships between traits and genotype distribution. *K*‐means clustering was conducted on PC1 and PC2 coordinates to identify distinct phenotypic groups. The optimal number of clusters was determined by evaluating average silhouette width and the gap statistic (Tibshirani et al., 2001) across *k* = 2–10, each with 25 random starts; both metrics independently identified *k* = 2 as optimal.

### 2.8 Genotype data

The current IMD panel is a subset of the original panel described by Sandhu and Singh (2021), and is based on the recent identity‐by‐state analysis by Chiteri et al. (2023). To ensure accurate physical mapping and resolve structural variations present in older assemblies (Kang et al., 2014), raw genotyping‐by‐sequencing data were aligned to the newly published gap‐free, telomere‐to‐telomere (T2T) *V. radiata* reference genome assembly *Weilv‐9* (Jia et al., 2025). Of the 372 genotypes evaluated phenotypically, four were excluded from the genomic analysis due to missing sequencing data. Consequently, the final set of 368 genotypes yielded 31,058 single nucleotide polymorphisms (SNPs) for further analysis (Boddepalliet al., 2026a). After the quality control in TASSEL v5.0 (trait analysis by association, evolution, and linkage) (Bradbury et al., 2007) using the following criteria: (i) minor allele frequency (MAF) ≥ 0.05, (ii) sites with less than 15% missing data, and (iii) maximum heterozygosity ≤10%, a total of 17,928 high‐quality SNPs were retained for further downstream analyses.

SNP names were originally assigned based on the first mungbean reference genome assembly (Kang et al., 2014) and have been retained in this naming convention across previously published studies utilizing the IMD panel (Boddepalli et al., 2026a; Chiteri et al., 2021, 2023; Sandhu & Singh, 2021) to preserve marker identity and enable direct comparison of associations across publications. Chromosome and physical position values reported throughout this manuscript reflect realignment to the gap‐free, T2T Weilv‐9 assembly (Jia et al., 2025) used in this study, while SNP identifiers retain their original Kang et al. (2014)‐based naming for continuity.

### 2.9 Genome‐wide association study

Genome‐wide association analyses (GWAS) were conducted using the phenotypic BLUEs for each genotype, generated by a MLM described in Section 2.5. Four complementary statistical approaches were performed to maximize power for QTL detection while controlling false discovery: three frequentist models, BLINK (Bayesian information and linkage‐disequilibrium iteratively nested keyway) (Huang et al., 2019), FarmCPU (fixed and random model circulating probability unification) (X. Liu et al., 2016), and single Locus MLM (Z. Zhang et al., 2010) in the genomic association and prediction integrated tool (GAPIT) package (version 3) (J. Wang & Zhang, 2021) and one Bayesian variable selection method, selection of variables with embedded screening using Bayesian methods (SVEN) (Li et al., 2022). We selected this multi‐model approach to cross‐validate associations, consistent with recent high‐throughput studies in mungbean (Boddepalli et al., 2026a; Boddepalli et al., 2026b; Chiteri et al., 2021, 2023). To control for multiple testing while accounting for the high linkage disequilibrium (LD) inherent in the mungbean genome, we determined the effective number of independent tests (*M*_eff_). Using PLINK v1.9 (Purcell et al., 2007) we performed LD‐based pruning with a sliding window of 50 SNPs, a step size of 5 SNPs, and an *r* ^2^ threshold of 0.2 (Gao et al., 2008). This analysis reduced the total of 17,928 markers to 2513 independent chromosomal segments. Consequently, the genome‐wide significance threshold was set at *α* = 0.05/2513 = 1.99 ×10^−5^ (−log_10_ *P* = 4.7). For SVEN, using a prior lambda of 0.1, markers were declared significant if their marginal inclusion probability exceeded 0.5, corresponding to a posterior odds ratio greater than 1 in favor of inclusion. Both principal components (PC.total = 3) and the default kinship matrix were generated within GAPIT and used as covariates in the model to control for population structure in the IMD panel. Manhattan and *Q*–*Q* plots were created using the results from BLINK, FarmCPU, and MLM models generated with the ggplot2 package (Wickham et al., 2007) in R.

To mitigate the upward bias in phenotypic variance explained (PVE) estimates typical of moderate‐sized mapping panels, a phenomenon known as the Beavis effect (Beavis, 1994; Xu, 2003), we implemented a post hoc nonparametric bootstrap resampling procedure (Sun & Bull, 2005; Xie et al., 2021). Specifically, for any significant locus exhibiting a MAF < 0.10 and an initial PVE point estimate exceeding 20%, we performed 5000 bootstrap iterations. The 95% confidence intervals for the PVE at these loci were subsequently derived from the resulting empirical distributions to provide a more conservative assessment of their true genetic effects.

To evaluate the cumulative genetic effect of the three major loci, we extracted allele dosage at each locus from PLINK‐recoded genotype files (*–recodeA*; Chr 1: 2_14494712_A; Chr 4: 5_35265704_C; Chr 6: 5_21173402_A), where a dosage of 2 indicates homozygous presence of the superior allele. We then summed the superior‐allele counts across all three loci (ranging from 0 to 3) to classify each accession into one of four allele‐dosage groups. We compared the group mean SWT values using one‐way analysis of variance followed by Tukey's HSD test (*α* < 0.05; *agricolae* package in R). Furthermore, for the 11 accessions that carried all three superior alleles in homozygous state, we constructed a morphological profile bubble plot by plotting SA against SWT with bubble size and color scaled to SW, derived from multi‐environment BLUEs estimates.

### 2.10 Candidate gene identification

Significant SNPs associated with more than one trait were selected for further candidate gene identification and comparative mapping studies. Genes located within a specified genomic interval surrounding each significant SNP were retrieved using the reference genome *Weilv‐9* (Jia et al., 2025) by following the methods outlined by Boddepalli et al. (2026a). To define the search window, we applied the average LD decay distance of 223.6 kb, estimated for this population (Boddepalli et al., 2026a). This resulted in a total search window of 447.2 kb, with 223.6 kb upstream and 223.6 kb downstream of each SNP. Based on gene annotations and Gene Ontology terms retrieved from the reference genome, candidate genes involved in seed development were identified. To evaluate the evolutionary conservation and functional relevance of the identified candidate genes, protein sequences for these genes were extracted from the Legume Information System (LIS) using the JBrowse tool. Then we conducted comparative mapping analysis using BLASTp (Camacho et al., 2009) across four legume species: soybean (*Glycine max* (L.) Merr.), cowpea (*Vigna unguiculata* (L.) Walp.), adzuki bean (*Vigna angularis*), and common bean (*Phaseolus vulgaris*) protein datasets available in LIS, as well as against *Arabidopsis thaliana* in TAIR (https://www.arabidopsis.org/tools/blast/) using an *E* value threshold of 1e‐10. Hits from *Arabidopsis* were further examined to identify functions consistent with seed size and shape‐related traits.

### 2.11 GP analysis

GP analysis was conducted for seven seed size and shape traits using a genomic best linear unbiased prediction (gBLUP) model (VanRaden, 2008), fitted with the rrBLUP package (Endelman, 2011) in R (Pérez & de los Campos, 2014). Phenotypic BLUEs for seven seed traits were derived for each genotype. A total of 17,928 SNPs, corresponding to 368 genotypes with complete data, were used to construct a genomic relationship matrix using the A.mat function in the rrBLUP package. An inbuilt expectation‐maximization option was used to impute missing markers, with a threshold of 0.5. The following mixed model was fitted for each trait:

y=Xβ+Zg+e

where **y** is the vector of line BLUEs for the genotype; **X** is the design matrix for fixed effects; **β** is the vector of fixed effects; **Z** is the design matrix linking genotypes to random effects; **g** is the vector of random additive genetic effects with **g** ∼ *N* (0, **K** $\sigma_{g}^{2}$), where **K** is the genomic relationship matrix, *σ_g_* ^2^ is the additive genetic variance; and *e* is the residual term with *e* ∼ *N* (0, **I** $\sigma_{e}^{2}$), where **I** is the identity matrix. This specification corresponds to the standard genomic BLUP model, in which all markers contribute to the genetic signal through **K** under a common prior variance for marker effects. In a second scenario (Gblup + SNP), we identified the top three significant SNPs for each trait based on their percentage of PVE and included them as fixed covariates in **X,** along with the random genomic effect (D. Li et al., 2019).

The model's predictive ability was assessed using repeated *k*‐fold cross‐validation (*k* = 10), with genotypes randomly partitioned into approximately equal‐sized groups. To minimize the bias introduced by population structure, we performed 100 repetitions of the 10‐fold cross‐validation, ensuring robust estimation of prediction accuracy across different training set compositions. In a given trait and cycle, nine folds were utilized as the training dataset, while the remaining fold served as a validation set by setting the phenotype values to missing prior to fitting the model. Predicted values (GEBVs) (Table S3) were extracted for the genotypes in the validation set to compare with their observed BLUEs. Pearson correlation between observed and predicted values in the validation fold was calculated to quantify the prediction accuracy.

Selection coincidence was assessed to evaluate the agreement between the top genotypes selected by GP models and those selected by phenotype‐based methods. To achieve this, genotypes were ranked for each trait based on their BLUEs (observed) and out‐of‐sample GEBVs (predicted). To ensure strict independence and prevent any in‐sample bias, the final GEBV vector was constructed by aggregating only the masked, held‐out predictions across the validation folds. The coincidence index (CI) was then computed as the percentage of shared genotypes within the top 20 individual rankings.

We additionally performed leave‐one‐cluster‐out (LOCO) cross‐validation to assess the influence of population structure on prediction accuracy. Genotypes were assigned to four subpopulations via *k*‐means clustering on the first three genomic principal components; *k* = 4 was validated using average silhouette width and the gap statistic (Tibshirani et al., 2001) across *k* = 2–10 (25 random starts per *k*), with both metrics identifying *k* = 4 as optimal. For each fold, all genotypes in one cluster were masked as the test set, and the remaining three clusters were used to train kin.blup (rrBLUP). The process was repeated until each cluster served as the test set once. Selection coincidence for LOCO was assessed using the same top‐20 CI described above, applied identically to both validation schemes.

## 3 RESULTS

### 3.1 Comparative performance: Deep learning versus traditional thresholding

We visually compared the SAM to a traditional HSV color‐thresholding method (Figure 2) for distinct seed colored genotypes. The HSV pipeline performed adequately on uniformly colored seeds but struggled with the phenotypic complexity present in a broader diversity panel. Specifically, the global color thresholds often misclassified dark pigmentation, mottling, and surface glare as background noise, leading to fragmented or incomplete seed masks (Figure 2A, left). Manually adjusting these thresholds often resulted in adjacent seeds merging into unrecognizable artifacts. In contrast, the SAM workflow consistently produced accurate and complete binary masks across all tested genotypes (Figure 2B, right).

### 3.2 Validation of automated seed trait extraction

To confirm the accuracy of SAM for high‐throughput phenotyping, digital morphological measurements were correlated with manual ground‐truth data (Table S1) for a subset of seeds representing four distinct seed‐coat‐color genotypes (green, yellow, mottled, and black; 60 seeds per genotype, 240 total). Of these, 166 seeds (69%) were successfully matched between SAM output and manual measurements; the remaining seeds were excluded due to SAM detection failures, which were disproportionately concentrated in black‐seeded genotypes (42% retention) relative to green, yellow, and mottled genotypes (70%–93% retention), likely reflecting reduced contrast between dark seed coats and the imaging background.

**FIGURE 3:**
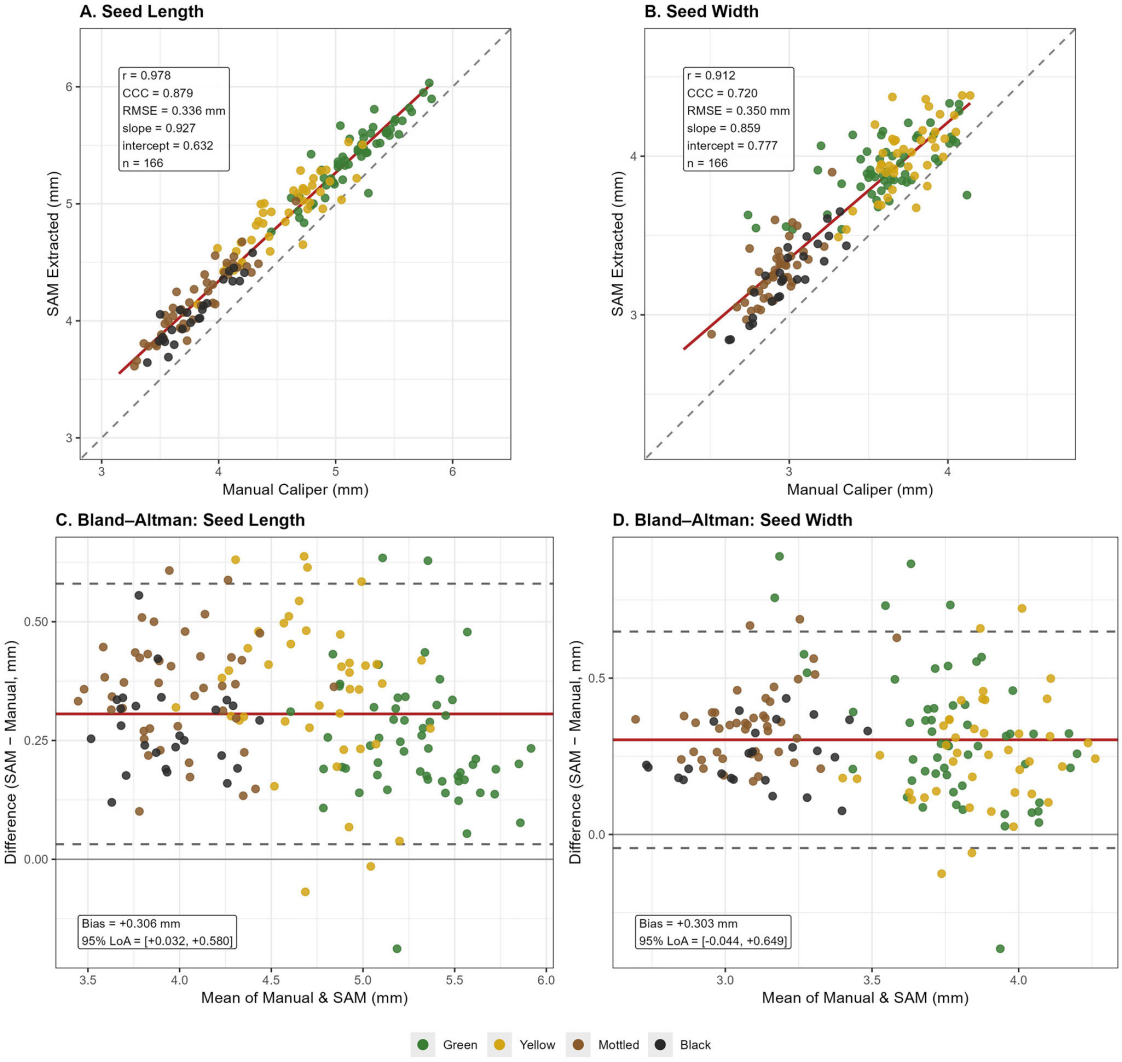
Validation of the segment anything model (SAM) for automated seed trait extraction. (A, B) Linear regression comparing manual caliper measurements (*x*‐axis) against SAM‐extracted measurements (*y*‐axis) for seed length (SL) and seed width (SW), pooled across 166 validated seeds spanning four seed‐coat‐color genotypes. Dashed line = 1:1 agreement; red line = fitted regression. (C, D) Corresponding Bland–Altman plots showing measurement difference versus mean, with mean bias (red line) and 95% limits of agreement (dashed lines). Pearson *r* = 0.98 (SL) and 0.91 (SW); linear regression slope = 0.927 (SL), 0.859 (SW), intercept = 0.632 (SL), 0.777 (SW); root mean square error (RMSE) = 0.336 mm (SL) and 0.350 mm (SW); concordance correlation coefficient (CCC) = 0.88 (SL) and 0.72 (SW); mean bias = +0.31 mm (SL) and +0.30 mm (SW).

**FIGURE 4:**
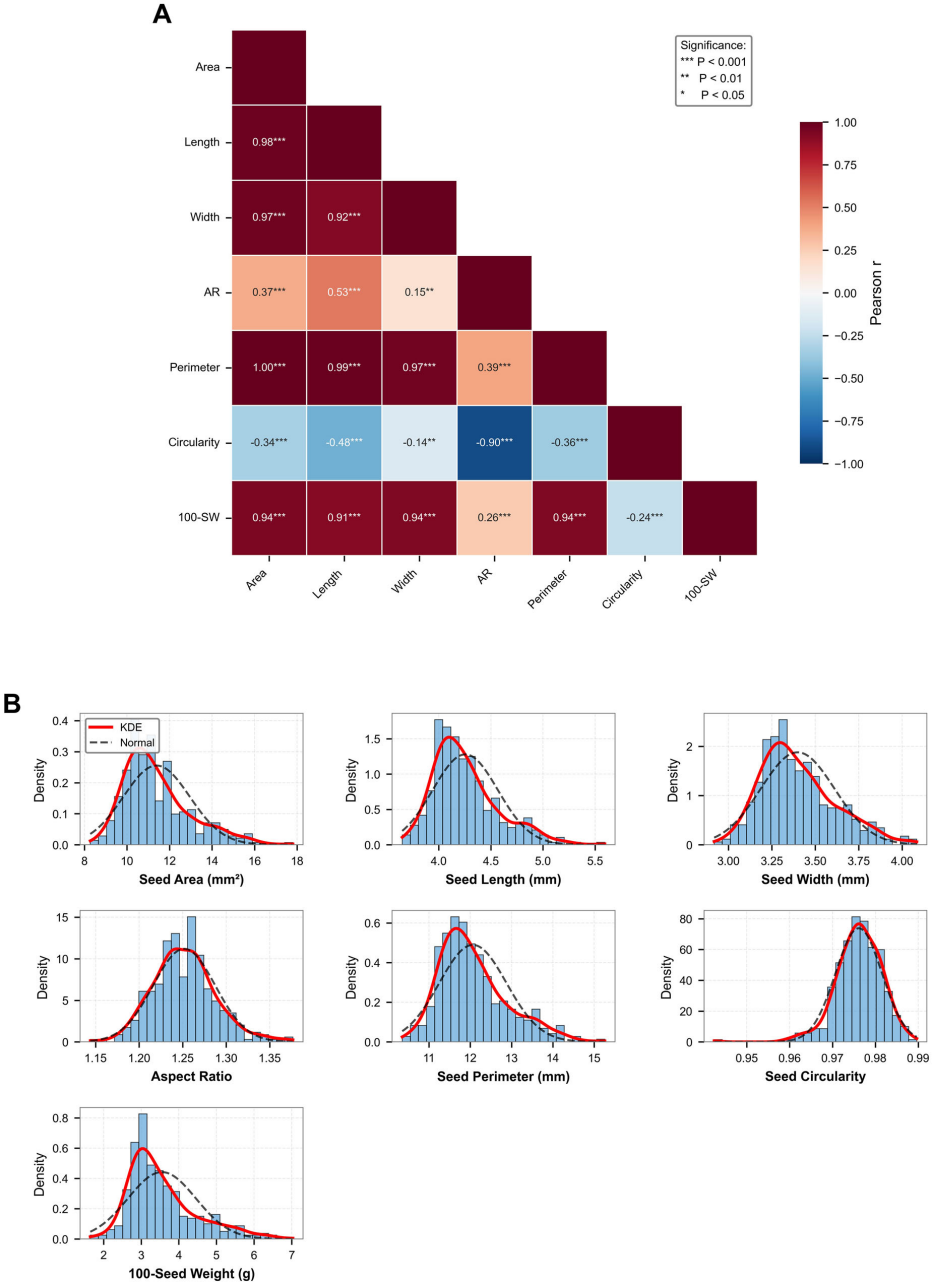
Correlation matrix and frequency distributions of seed size and weight traits in Iowa mungbean diversity (IMD) panel. (A) Heatmap showing pairwise Pearson correlation coefficients among seven seed traits. Statistical significance is denoted by asterisks, ***p < 0.001, **p < 0.01, *p < 0.05. (B) Frequency distributions of individual seed traits showing histograms.

Pearson correlation analysis of the matched 166‐seed set showed strong agreement between SAM‐derived and manually measured dimensions, with a correlation coefficient (*r*) of 0.98 (*p* < 0.001) for SL and 0.91 (*p* < 0.001) for SW (Figure 3A,B) and a root mean square error of 0.336 mm (SL) and 0.350 mm (SW). However, the concordance correlation coefficient (CCC), which jointly accounts for both correlation and measurement bias, was more moderate (CCC = 0.88 for SL, 0.72 for SW), and Bland–Altman analysis revealed a consistent positive bias of +0.31 mm (SL) and +0.30 mm (SW) (Figure 3C,D), with consistent in direction across all four seed‐coat colors, though its magnitude and the strength of within‐color agreement varied (Table S4), with black seeds showing the highest within‐color CCC (SW: 0.90) despite their lower detection rate. Because SA and SPM cannot be measured directly with digital calipers, these two traits were validated indirectly through their strong correlation with SWT (*r* = 0.94, Figure 4A) rather than against a physical ground‐truth measurement.

### 3.3 Descriptive statistics and correlation of seed size, shape, and weight traits

The IMD panel exhibited significant phenotypic diversity across all evaluated seed traits, displaying continuous variation characteristic of polygenic quantitative traits (Figure 4B). While dimensional traits such as SL, SW, and SA exhibited approximately symmetric distributions, traits such as SWT and SAR displayed slight skewness. Seed size parameters, including SL, SW, and SA, showed moderate variation with mean values of 4.24, 3.39, and 11.33 mm^2^, respectively (Table 2). While most size‐related traits showed low coefficients of variation (CV% ≤ 17), SWT showed the highest phenotypic variation (CV% = 27.76), ranging from 1.41 to 7.59 g. Broad‐sense heritability (*H* ^2^) estimates were consistently high for both size traits and shape traits (*H* ^2^ ≥ 0.88), particularly for SWT (*H* ^2^ = 0.99), which also recorded the highest genotypic variance (*V_g_* = 0.82).

**TABLE 2:** Descriptive statistics, variance components, and broad‐sense heritability for seven seed size, shape, and weight traits in the 368‐genotype mungbean diversity panel, evaluated across three environments.

Trait	*N*	Mean	Min	Max	SD	CV (%)	*V*_g_	*V*_env_	*V*_ge_	*V*_e_	*H* ^2^
100‐seed weight (SWT, g)	2189	3.54	1.41	7.59	0.98	27.76	0.8276	0.0450	0.0001	0.1125	0.99
Seed length (SL, mm)	2143	4.24	3.20	5.84	0.39	9.20	0.0998	0.0008	0.0043	0.0493	0.96
Seed width (SW, mm)	2147	3.39	2.57	4.40	0.28	8.17	0.0458	0.0082	0.0021	0.0240	0.96
Seed area (SA, mm^2^)	2136	11.33	5.90	19.34	1.96	17.29	2.5610	0.1754	0.1051	1.0872	0.96
Seed aspect ratio (SAR)	2143	1.26	1.11	1.44	0.05	4.23	0.0011	0.0009	0.0001	0.0011	0.91
Seed perimeter (SPM, mm)	2138	12.03	9.09	15.91	1.03	8.53	0.6957	0.0343	0.0259	0.3188	0.96
Seed circularity (SCR)	2144	0.98	0.94	1.00	0.01	0.89	1.93e‐05	3.12e‐05	1.99e‐06	3.40e‐05	0.88

*Note*: *V*_g_, genotypic variance (line, random); *V*_env_, variance among environments; *V*_ge_, genotype × environment interaction variance; *V*_e_, residual (error) variance; *H* ^2^, broad‐sense heritability on an entry‐mean basis.

Pearson correlation analysis revealed a strong association between seed size and shape traits (Figure 4A). Highly significant positive correlations (*r* ≥ 0.91, *p* < 0.001) were observed among all size traits, including SL, SW, SA, and SPM, as well as all of them with SWT. SCR was significantly and negatively correlated with all size and weight traits (*r* = −0.14 to −0.48; *p* < 0.001. SAR, by contrast, was significantly and positively correlated with all size and weight traits (*r* = 0.15–0.53; *p* < 0.01–0.001), with the weakest association observed for SW (*r* = 0.15, *p* < 0.01). SAR and SCR were themselves strongly and negatively correlated (*r* = −0.90, *p* < 0.001), indicating that more elongated seeds (higher SAR) tend to be less circular.

**FIGURE 5:**
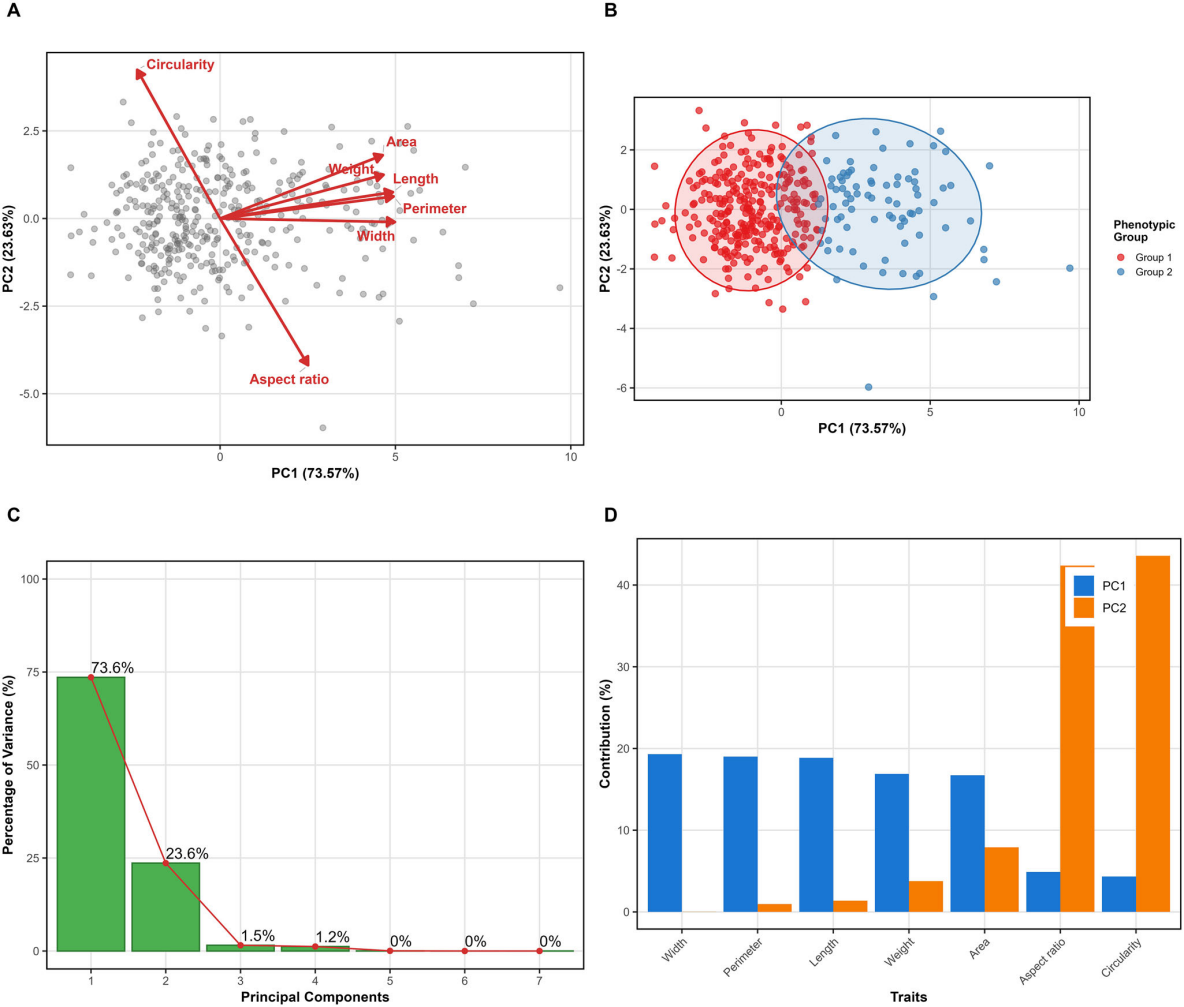
Phenotype‐based principal component analysis of seed traits in the Iowa mungbean diversity (IMD) panel. (A) principal component analysis (PCA) biplot showing the distribution of the genotypes (gray points) and trait loading vectors (red arrows) for seven seed traits. (B) Phenotypic clustering based on *k*‐means analysis, with genotypes clustered into two distinct groups. (C) Scree plot displaying the percentage of variance explained by each principal component. (D) Bar chart showing the contribution of individual traits to PC1 and PC2.

PCA of seed traits identified that the first two components (PC1 and PC2) collectively explained 97.2% of the total phenotypic variance (73.6% and 23.6%, respectively) (Figure 5). When examining the trait loadings within these components, seed size and weight traits predominantly drove PC1 (accounting for 90.8% of its composition), while PC2 was primarily driven by seed shape traits (SAR and SCR), which accounted for 85.8% of its composition (Figure 5A,D). Furthermore, *K*‐means clustering, validated using silhouette width and the gap statistic (Section 2.7), identified two distinct phenotypic groups (Figure 5B), broadly distinguished by overall seed size, with cluster 1 (*n* = 288) comprising smaller‐seeded genotypes and cluster 2 (*n* = 83) comprising larger‐seeded genotypes (Table S5).

### 3.4 GWAS results

**FIGURE 6:**
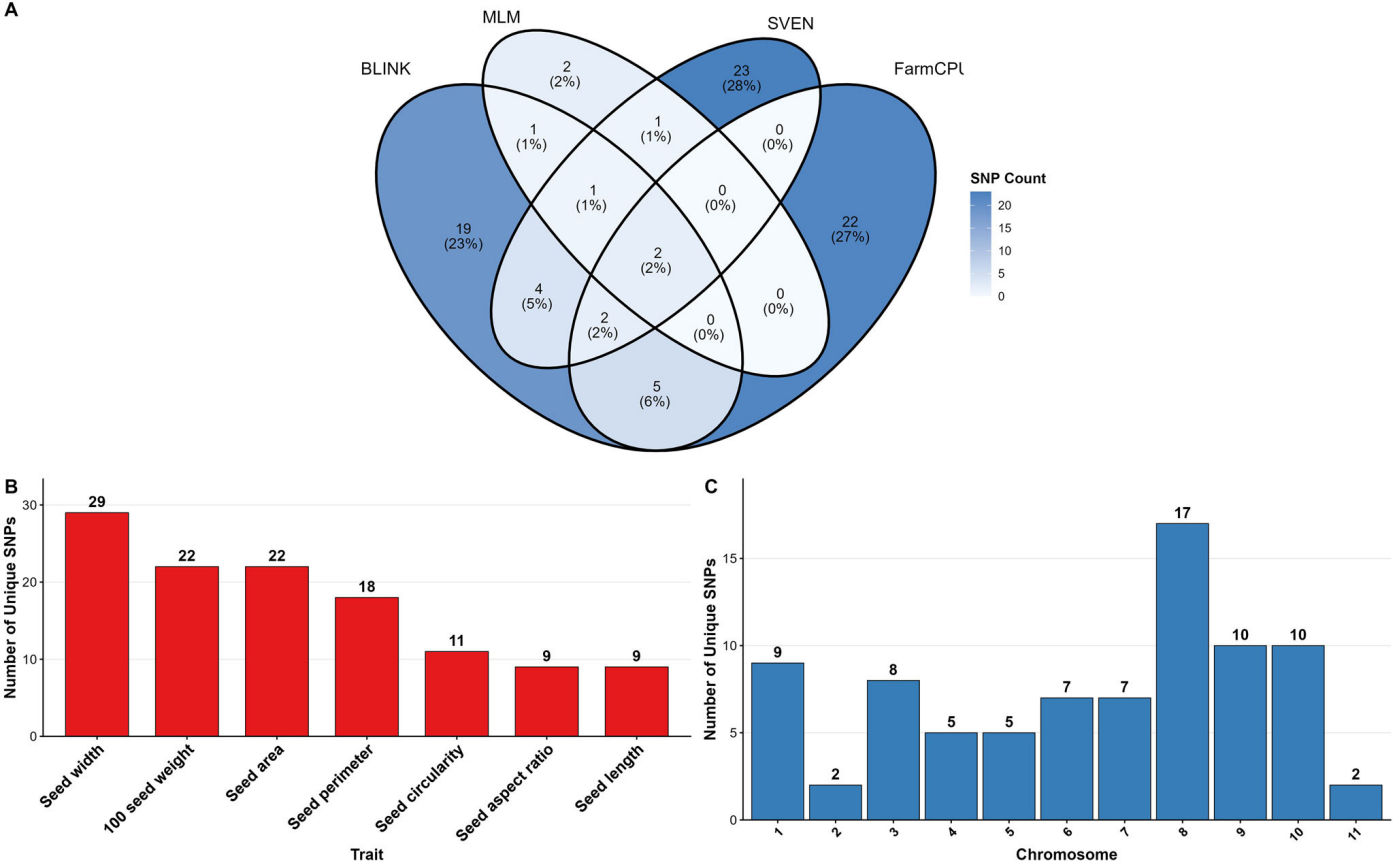
Distribution and overlap of significant single nucleotide polymorphisms (SNPs) identified by genome‐wide association studies (GWAS) for seed size, weight, and shape traits. (A) Venn diagram showing the overlap of significant SNPs detected by four GWAS methods (Bayesian‐information and linkage‐disequilibrium iteratively nested keyway [BLINK], mixed linear model [MLM], selection of variables with embedded screening using Bayesian methods [SVEN], and fixed and random model circulating probability unification [FarmCPU]). (B) Trait‐wise distribution of unique SNPs across seven seed traits. (C) Chromosomal distribution of unique SNPs across the mungbean genome.

Using seed morphological parameters derived from the SAM‐based phenotyping pipeline, we identified 145 marker–trait associations (MTAs) across the four GWAS models (Table S6). Accounting for MTAs overlapping across GWAS models and phenotypes, a total of 82 unique genomic loci were identified. As illustrated in Figure 6, significant MTAs were identified across the four GWAS models, with notable consensus among the models in identifying major genomic regions. The identified associations were distributed across the genome, with chromosome 8 as a hotspot with the highest number of significant markers (16), followed by chromosomes 9, 1, and 3.

**FIGURE 7:**
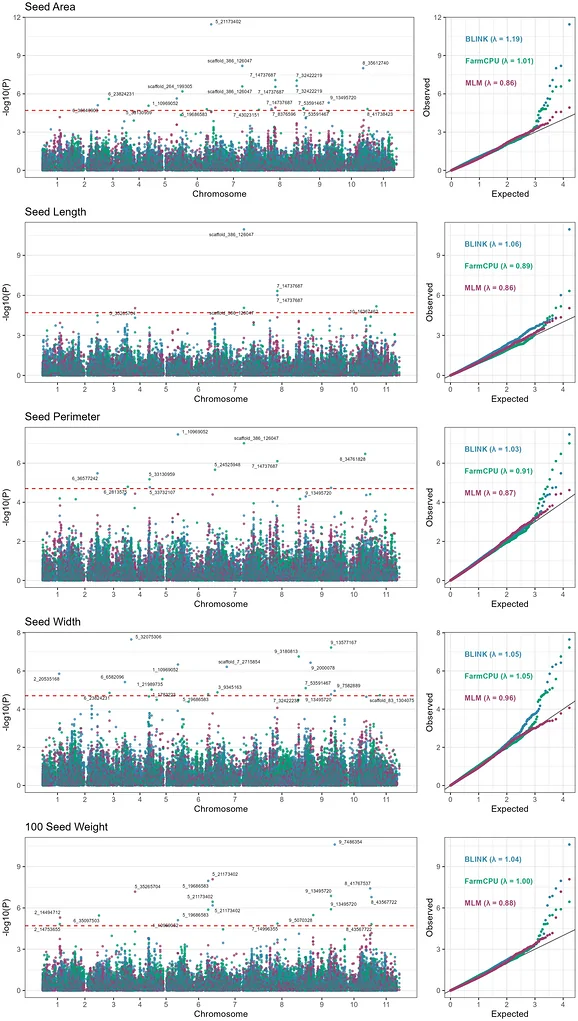
Manhattan and corresponding *Q*–*Q* plots showing the genome‐wide association studies (GWAS) results from Bayesian‐information and linkage‐disequilibrium iteratively nested keyway (BLINK) (green color), FarmCPU (fixed and random model circulating probability unification) (Blue color), and mixed linear model (MLM) (magenta color) models for seed size (seed length [SL], seed width [SW], seed area [SA], and seed perimeter [SPM]) and weight (100‐seed weight [SWT]) traits. The red dashed line indicates that the genome‐wide significance threshold was set at *α* = 0.05/2513 = 1.99 ×10^−5^ (−log_10_ *P* = 4.7).

**FIGURE 8:**
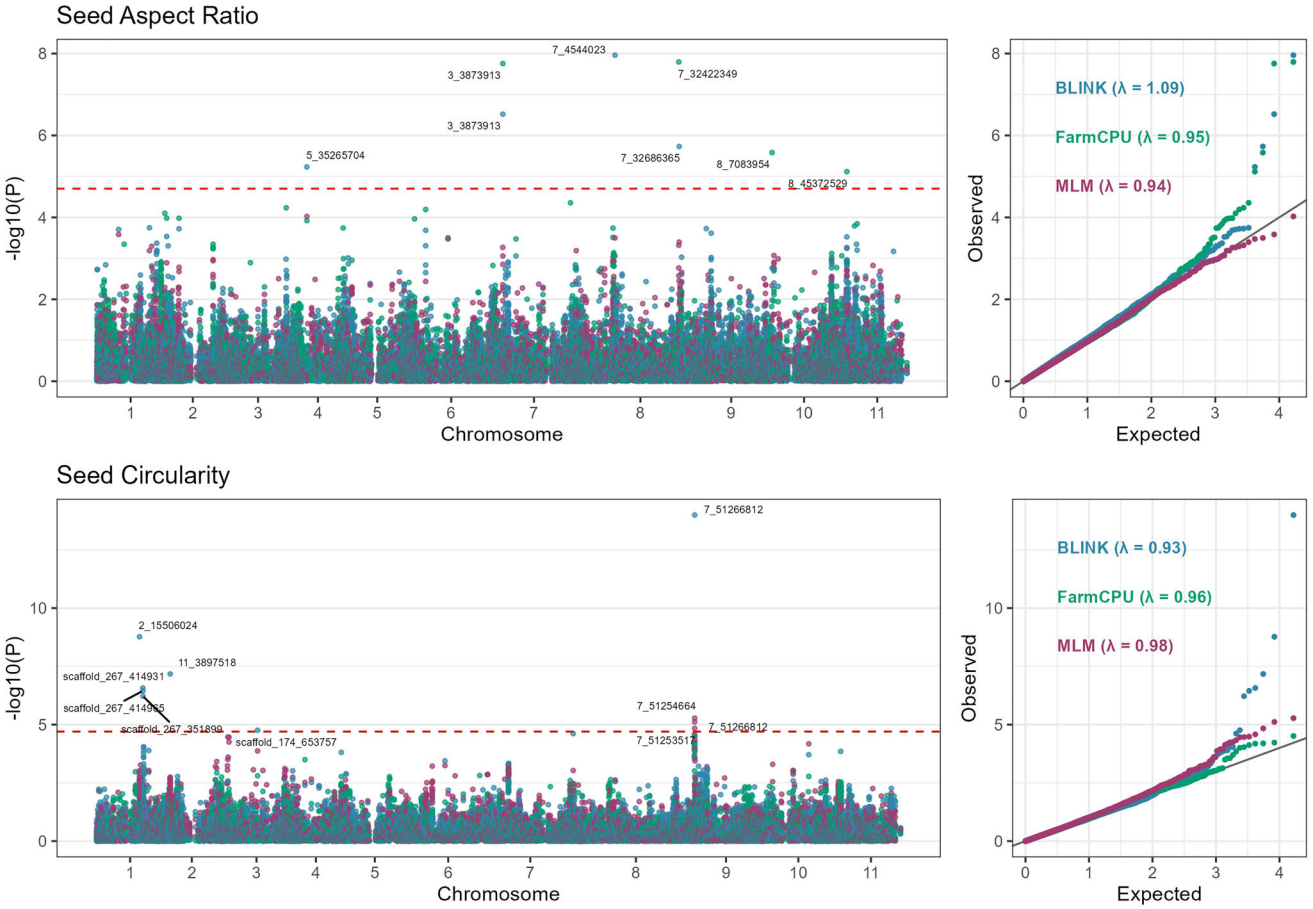
Manhattan and corresponding *Q*–*Q* plots showing the genome‐wide association studies (GWAS) results of seed shape traits (seed aspect ratio [SAR] and seed circularity [SCR]) from Bayesian‐information and linkage‐disequilibrium iteratively nested keyway (BLINK) (green color), fixed and random model circulating probability unification (FarmCPU) (blue color), and mixed linear model (MLM) (magenta color) models. The red dashed line indicates the genome‐wide significance threshold was set at *α* = 0.05/2513 = 1.99 ×10^−5^ (−log_10_ *P* = 4.7).

Manhattan and *Q*–*Q* plots (Figures 7 and 8) illustrate the distinct genomic hotspots for seed size, weight, and shape, revealing that while seed shape traits (SCR and SAR) were significantly associated with Chromosomes 1, 7, and 8, seed weight and size traits exhibited a more integrated genetic architecture characterized by eight common MTAs. Within Chromosome 8, SNPs 7_4544023 (21.96 Mbp) and 7_51266812 (73.85 Mbp) were identified across two GWAS models as being linked to SAR and SCR, respectively. Further analysis revealed 13 key pleiotropic SNPs associated with three or more traits, including SNP 7_14737687 on Chromosome 8 (31.13 Mbp), which was consistently identified by all four models as being associated exclusively with seed size traits such as SA, SL, and SP. Similarly, the SNP scaffold_386_126047 on Chromosome 7 (40.12 Mbp) was colocalized across three GWAS models and also showed exclusive association with these traits of similar size. Notably, three high‐confidence genomic regions for seed size and weight were identified on Chromosomes 1, 4, and 6, which demonstrated PVE values above 20% and consistent colocalization across multiple GWAS models.

**FIGURE 9:**
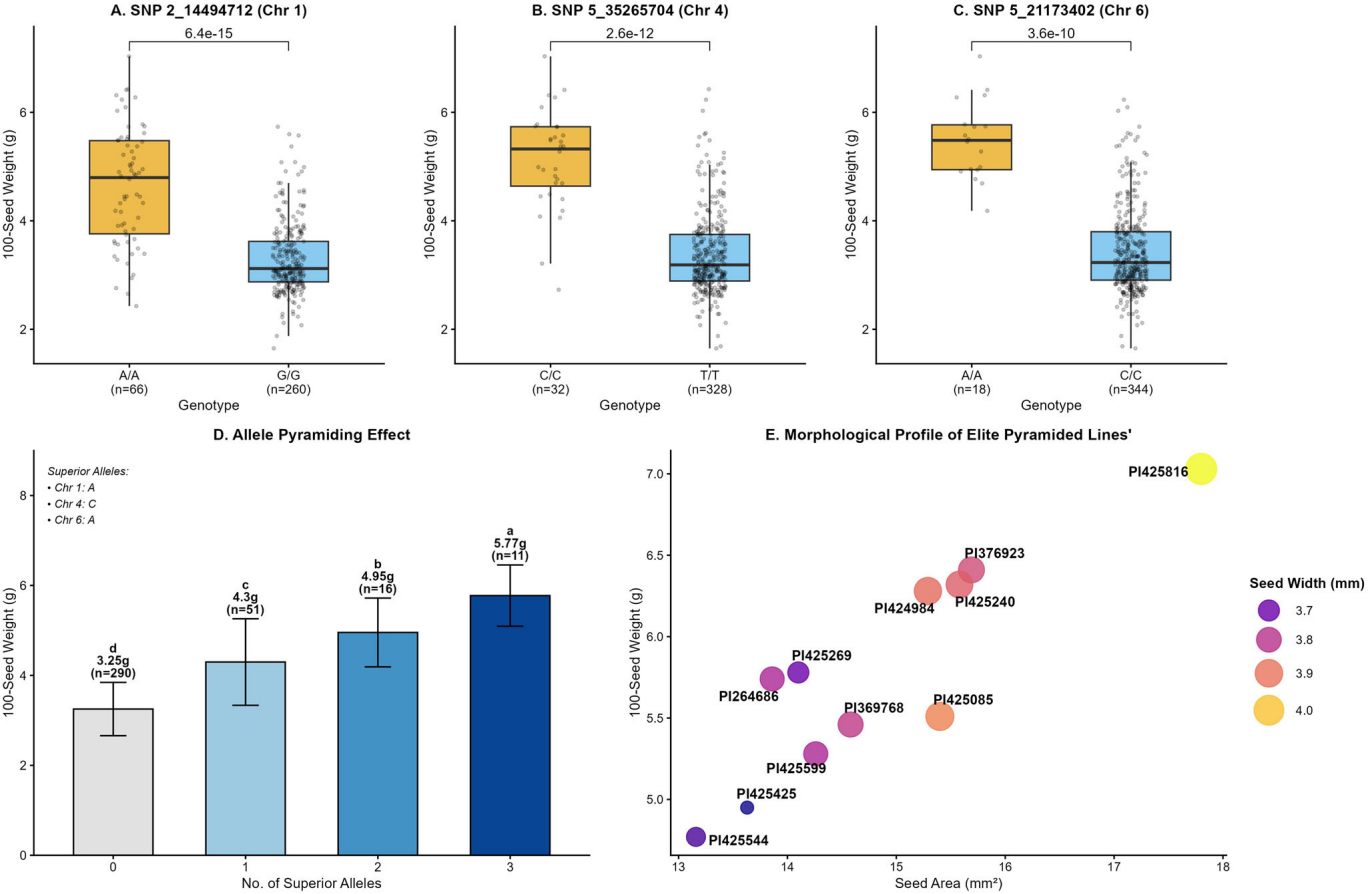
Phenotypic validation and additive effect of major‐effect genomic loci on 100‐seed weight (SWT). Boxplots illustrate the distribution of SWT (g), with sample size (*n*) indicated above each allele class, for major and minor allelic classes at three high‐confidence loci: (A) Single nucleotide polymorphism (SNP) 2_14494712 on Chromosome 1, (B) SNP 5_35265704 on Chromosome 4, and (C) SNP 5_21173402 on Chromosome 6. (D) Allele pyramiding effect illustrating the cumulative additive effect of superior alleles on seed weight. The bar chart represents the mean SWT (g) for accessions grouped by the total number of superior alleles carried across the three validated loci (Chr 1: A, Chr 4: C, and Chr 6: A). Error bars represent the standard deviation (SD). The mean SWT and number of accessions (*n*) for each group are indicated above the bars, and different lowercase letters denote statistically significant differences based on Tukey's HSD test (*p* < 0.05). (E) Morphological profile of the 11 elite accessions possessing all three superior alleles. The bubble plot demonstrates the pleiotropic relationship between seed area (SA) (mm^2^) and SWT (g), with both bubble size and color gradient corresponding to seed width (SW) (mm).

Post hoc validation using these markers confirmed significant allelic effects at each locus (Figure 9; Table 3). An SNP 2_14494712 (24.41 Mb) on chromosome 1 was associated with SA, SPM, and SWT, explaining up to 31.6% of the PVE. Accessions homozygous for the alternate allele (A) exhibited a mean SWT of 4.63 ± 0.12 g, a 40.2% increase over those carrying the major allele (G; 3.30 ± 0.05 g; *p* < 0.0001; Figure 9A). Another pleiotropic SNP, 5_35265704 (7.22 Mb), on chromosome 4, was associated with SL, SWT, SAR, and SCR. The alternate allele (C) conferred a 51.5% advantage in SWT (5.13 g vs. 3.39 g; *p* < 0.0001; Figure 9B). The most significant association was identified on Chromosome 6 at 42.48 Mb (SNP 5_21173402). This locus was consistently detected by all four GWAS models and exhibited the largest single‐marker effect observed in this study. The alternate allele (A) increased seed weight by 60% (5.47 g vs. 3.43 g; *p* < 0.0001; Figure 9C), with an MAF of 0.06 and over 20% PVE.

**TABLE 3:** Description of the significant marker‐trait associations collectively identified by Bayesian‐information and linkage‐disequilibrium iteratively nested keyway (BLINK), fixed and random model circulating probability unification (FarmCPU), mixed linear model (MLM), and selection of variables with embedded screening using Bayesian methods (SVEN) models, and the associated candidate genes extracted from the mungbean genome vigra.Weilv‐9.gnm1.ann1 at legume information system (LIS).

SNP ID	Trait	Chromosome: Position (Weilv‐9)	−Log_10_ *P*	MIP	MAF	REF/ALT	Effect	PVE	Model	Candidate gene	Gene description
1_10969052	SA	5:38876574	5.64	–	0.22	G/A	0.25	3.19	B	Virad05G0159800	Hydroxymethylglutaryl‐CoA synthase
	SW		6.34	–			0.03	2.24			
	SPM		7.47	–			0.16	3.22			
	SWT		5.11	–			0.1	3.02			
2_14494712	SA	1:24413902	–	1	0.2	G/A	−2.17	29.3	S	Virad01G0084400	ABC transporter B family member 15‐like
	SW		–	0.92			−0.28	26.16			
	SPM		–	0.98			−1.11	28.12			
	SWT		5.3				−0.23	31.62	M, S		
5_19686583	SWT	6:36281948	7.97		0.07	C/A	−0.25	5.5	B, F	Virad06G0194800	F‐box/kelch‐repeat protein
	SA		4.8				−0.33	6.46	F		
	SW		4.78				−0.04	6.8			
5_21173402	SA	6:42482684	11.45		0.06	C/A	−0.75	20.71	B, S	Virad06G0255600	Calmodulin‐binding protein (NPGR1)
	SPM		–	1			−1.63	20.04	S		
	SWT		8.08				−0.54	26.29	M, B, F, S		
5_35265704	SL	4:7227756	5.04		0.1	T/C	−0.13	23.65	M, S	Virad04G0076700	Ethylene‐responsive transcription factor
	SWT		7.18				−0.39	29.17	M		
	SAR		5.49				0.01	7.11	B		
	SCR		–	0.9			0	3.96	S		
6_36577242	SL	3:267038	–	0.8	0.18	T/C	0.27	10.12	S	Virad03G0002200	Serine/threonine‐protein kinase
	SPM		5.48				0.13	10.57	B		
	SWT		–	0.88			0.72	8.96	S		
6_6502133	SA	3:38600156	–	0.83	0.37	A/G	−0.88	7.04	S	Virad03G0229500	AP‐3 complex subunit beta
	SL		–	0.99			−0.17	6.43			
	SW		–	0.71			−0.13	7.58			
	SPM		–	0.98			−0.46	7.04			
7_14737687	SA	8:31133824	7.08	–	0.09	A/T	−0.4	5.44	M, B, F, S	Virad08G0252800	Cytochrome P450
	SL		6.02	–			−0.08	5.21	B, F, S		
	SPM		6.11	–			−0.15	5.29	F, S		
8_36270211	SA	10:43643353	–	0.99	0.34	T/C	−1.99	35.25	S	Virad10G0208500	peroxidase family protein
	SW		–	0.99			−0.28	38.01			
	SPM		–	1			−1.02	34.13			
	SWT		–	1			−1.18	36.46			
9_10797185	SA	9:32066020	–	0.71	0.47	G/C	1.09	11.26	S	Virad09G0158800	Transcription factor bHLH62‐like
	SW		–	0.64			0.14	10.47			
	SWT		–	0.94			0.66	12.46			
9_13495720	SA	9:30855923	5.31	–	0.5	C/T	−0.2	11.89	B	Virad09G0150600	LRR receptor‐like kinase
	SW		4.77	–			−0.03	12.83			
	SPM		4.74	–			−0.1	11.25			
	SWT		5.9	–			−0.11	13.81	F		
9_13579483	SA	9:30932950	–	0.86	0.49	A/C	1.14	12.37	S	Virad09G0151000	Chromatin modification‐related protein EAF1 B
	SW		–	0.61			0.16	13.29			
	SPM		–	0.63			0.58	11.75			
scaffold_386_126047	SA	7:40129332	8.19	–	0.07	C/T	−0.83	27.59	B, F	Virad07G0252800	Coumaroyl‐CoA: anthocyanidin 3‐O‐glucoside‐6′'‐O‐coumaroyltransferase 1
	SL		10.94	–			−0.2	25.86	B, F, S		
	SPM		7.02	–			−0.38	26.77	F		

*Note*: SNP ID follows the Kang et al. (2014) marker‐naming convention; the chromosome: position column reflects current Weilv‐9 (Jia et al., 2025) coordinates (see Section 2.8). The marginal inclusion probability (MIP) resulting from the SVEN model was highlighted in bold numbers to distinguish from *p*‐values. MIP values close to 1 are highly significant associations. Short letters for genome‐wide association studies (GWAS) models were mentioned in the table: M, MLM; B, BLINK; F, FarmCPU, and S, SVEN. REF, Reference allele; ALT, alternate allele.

Abbreviations: MAF, minor allele frequency; PVE, phenotypic variance explained; SA, seed area; SAR, seed aspect ratio; SCR, seed circularity; SL, seed length; SNP, single nucleotide polymorphism; SPM, seed perimeter; SW, seed width; SWT, 100‐seed weight.

Analysis of additive effects from combining superior alternate alleles associated with these three QTLs revealed a significant dosage effect (Figure 9D). Genotypes lacking these alleles had an average weight of 3.25 g. However, the presence of a single alternate allele increased the average weight to 4.30 g, a 32% increase, and with two superior alleles, it increased to 52% (4.95 g). Notably, the 11 large‐seed‐weight lines (Table S2) that carried superior alleles at all three loci, such as PI425816 and PI376923, achieved a mean seed weight of 5.77 g (Figure 9E). This represents a 77.5% phenotypic difference from the baseline weight observed within the extremes of this specific diversity panel.

Based on nonparametric bootstrap resampling (5000 replicates) for loci with MAF ≤ 0.10 and point‐estimate PVE > 20% (Table S7), four loci were retained: 5_21173402 and 5_35265704 (both associated with SWT), and scaffold_386_126047 (associated with both SA and SL). All four showed substantially non‐zero 95% CI lower bounds (15.9%–18.4%), indicating these effects are unlikely to be artifacts of sampling variation despite their low allele frequency.

### 3.5 Candidate gene analysis results

A total of 13 markers, each associated with more than three traits and characterized by high PVE in at least one GWAS model, were prioritized for downstream candidate gene analysis (Table 3). These markers are linked to genes involved in diverse biological processes, including signal transduction (protein phosphorylation and kinase activity), intracellular trafficking (AP‐3 adaptor complex), and structural development (pectin esterase activity and cell wall modification).

Several high‐confidence candidates were identified within or near these loci. Notably, marker 2_14494712 lies within the LD‐decay‐based candidate gene search window of *Virad01G0084400*, an ABC transporter protein associated with ATP binding and transmembrane transport. Markers 5_21173402 and 5_35265704 were found within the intronic regions of *Virad06G0255600* (a calmodulin‐binding protein, NPGR1) and *Virad04G0076700* (an ethylene‐responsive transcription factor [ERF]), respectively. Functional annotations also revealed key stress‐responsive genes, such as a dehydration‐responsive element‐binding protein (*Virad03G0002200*) associated with marker 6_36577242. Other notable candidates include an AP‐3 complex subunit beta (*Virad03G0229500*) and a Nup88 domain‐containing protein (*Virad09G0150700*) located within the LD region of marker 9_13495720. Finally, marker scaffold_386_126047 was mapped to the 3′ UTR of *Virad07G0252800*, which encodes a Coumaroyl‐CoA: anthocyanidin 3‐O‐glucoside‐6″‐O‐coumaroyltransferase 1 protein.

### 3.6 Comparative mapping study results

To investigate the evolutionary conservation and potential functional orthology of the top candidate genes associated with seed dimensional traits, a comparative mapping analysis was conducted. The protein sequences of the three most promising mungbean candidate genes (*Virad01G0084400*, *Virad04G0076700*, and *Virad06G0255600)* were queried against the proteomes of four closely related, agriculturally significant legume species: adzuki bean (*V. angularis*), cowpea (*V. unguiculata*), common bean (*P. vulgaris*), and soybean (*G. max*).

Marker 5_21173402 (Chr. 6), located within the LD region of *Virad06G0255600*, explained up to 26.3% of the phenotypic variance for seed size and weight. This gene exhibited the strongest cross‐species conservation among the queried loci, with all top alignments yielding *E*‐values of 0.0 and 100% query coverage. It shared near‐perfect amino acid sequence identity with closely related *Vigna* species, including adzuki bean (96.09%, *Vang05g04720.1*) and cowpea (95.68%, *Vigun09g231400.1*). Similarly, it is maintaining 93.16% identity with common bean (*Phvul. 009G071800.1*) and approximately 88% with soybean (Glyma.*04G045800.2* and *Glyma.06G046600.1*). Its closest *Arabidopsis* homolog, *AT1G27460* (NPGR1), encodes a calmodulin‐binding protein, expressed in flowers and fruits, suggesting a conserved role in reproductive organ development.

Candidate gene *Virad04G0076700* with SNP 5_35265704 (Chr. 4), demonstrated similar conservation, sharing 98.01% sequence identity with adzuki bean (*Vang09g02950.1*) and over 95% with cowpea (*Vigun05g223300.1*) and common bean (Phvul. *005G111200.1*). In soybean, two highly conserved paralogs were identified on chromosomes 13 and 12 (*Glyma. 13G304300.1* and *Glyma. 12G197700.1*), sharing 93.19% and 92.67% identity, respectively.

Similarly, marker 2_14494712 (Chr.1), located close to the ABC transporter gene *Virad01G0084400*, exhibited high protein sequence conservation primarily within the *Vigna* genus, sharing 96.86% identity with adzuki bean (*Vang0137s00290.1*) and 82.89% with cowpea (*Vigun11g076700.3*). Interestingly, sequence identity dropped substantially when compared to common bean (*Phvul.010G055200.1*; *47.54%*) *and soybean* (*Glyma.12G135600.1*; 52.87%). The corresponding *Arabidopsis* ortholog (*AT3G28345*) displays robust seed‐specific expression, further supporting its role in seed‐filling processes. Collectively, these comparative results reinforce the likelihood that the identified mungbean loci are integral components of a conserved genetic network governing legume seed characteristics.

### 3.7 Genomic prediction

**FIGURE 10:**
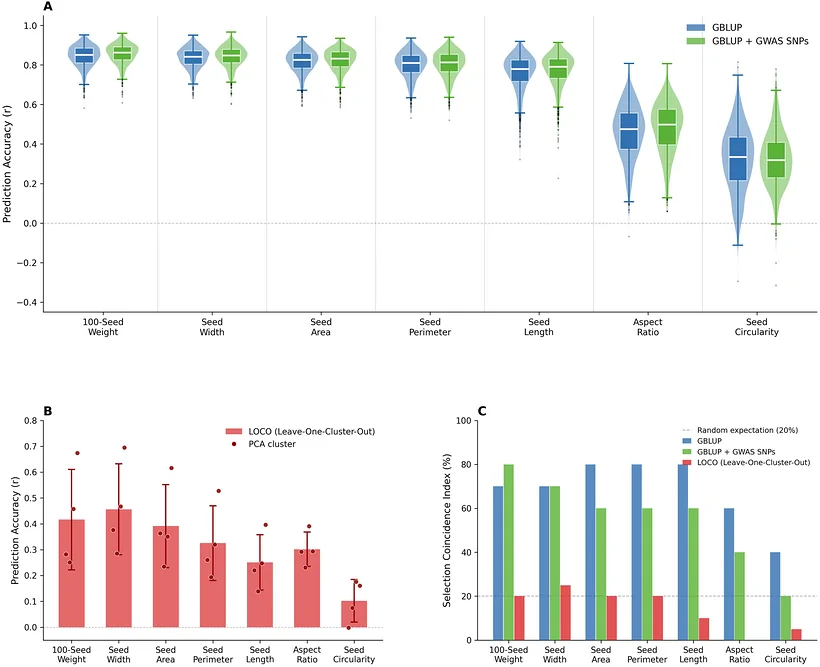
Genomic prediction accuracy and selection efficiency of seed size, weight, and shape traits. (A) Distribution of prediction accuracies for the genomic best linear unbiased prediction (Gblup) and gBLUP + single nucleotide polymorphisms (SNPs) models estimated across 100 cycles of 10‐fold cross‐validation, presented as violin plots with embedded box plots. Traits are ordered left to right by decreasing mean gBLUP accuracy. (B) Prediction accuracy of the leave‐one‐cluster‐out (LOCO) cross‐validation model across *K* = 4 principal component analysis (PCA)‐derived population clusters. Bars represent mean accuracy ± standard deviation; colored dots indicate cluster‐specific accuracy for each withheld population group (Clusters 1–4). (C) Selection coincidence index (CI) for the gBLUP, Gblup + SNPs, and LOCO models, representing the percentage overlap between the top 20 genotypes selected based on genomic estimated breeding values (GEBVs) and observed best linear unbiased estimation (BLUEs). The dashed line denotes the expected CI under random selection (20%).

GP analysis using two approaches, gBLUP based on genome‐wide markers, and an augmented model incorporating significant SNPs identified through GWAS (gBLUP + SNPs), was conducted for seven seed traits. Prediction accuracies were calculated through 100 cycles of 10‐fold cross‐validation, with results summarized in Figure 10A and Table S8. The gBLUP model yielded moderate to high prediction accuracies for most seed‐dimension traits. Among them, SWT exhibited the highest accuracy (*r* = 0.84), followed by SW (*r* = 0.83), SA (*r* = 0.82), and SPM (*r* = 0.80). SL showed slightly lower accuracy (*r* = 0.76). In contrast, substantially lower prediction accuracies were observed for seed shape traits, including SAR (*r* = 0.46) and SCR (*r* = 0.32), consistent with their lower heritability estimates and limited phenotypic variation.

The augmented approach (gBLUP + SNPs), incorporating significant GWAS‐identified SNPs into the prediction model, resulted in slight but consistent improvements in accuracy for seed size and weight traits. The most notable relative improvements were recorded for SAR (3.74%, *r* = 0.479) and SL (1.78%, *r* = 0.772), while SWT achieved the highest overall mean accuracy of 0.855 ± 0.051. Additionally, the augmented model improved prediction stability for highly heritable traits, as evidenced by lower standard deviations for SWT and SL compared to the gBLUP model.

Selection efficiency was assessed using the selection CI, which measured the overlap between the top 20 genotypes selected by genomic estimated breeding values (GEBVs) (Table S3) and observed BLUEs (Table S2) (Figure 10C). Despite systematic increases in genomic prediction accuracies (GPAs), the gBLUP + SNPs model only enhanced selection efficiency for SWT, resulting in a 10% increase in CI from 70% to 80%. Conversely, five traits, SL, SA, SPM, SAR, and SCR, exhibited a 20% reduction in CI when transitioning to the augmented model. SW maintained a stable CI of 70% across both models. These results indicate that while incorporating major‐effect SNPs as fixed covariates improves overall model fit, it can lead to re‐ranking of elite individuals, potentially reducing the efficiency of identifying the absolute top‐performing genotypes for certain seed shape and size parameters.

To evaluate whether the random‐CV accuracies above were inflated by relatedness between training and test sets, we additionally performed LOCO cross‐validation across four kinship‐based subpopulations (Figure 10B; Table S9). Across all seven traits, LOCO accuracies were substantially lower than random 10‐fold CV (Figure 10B; Table S9), with the largest absolute declines observed for SW (*r* = 0.83–0.46) and SWT (*r* = 0.84–0.42). The relative ranking of traits was largely preserved across schemes: SW, SWT, SA, and SPM remained the most predictable traits under both validation approaches, while circularity showed the lowest accuracy under both (*r* = 0.32 in random CV; *r* = 0.10 ± 0.08 in LOCO). Selection coincidence was correspondingly reduced under LOCO relative to random CV across all traits (Figure 10C; Table S9), ranging from 0% (SAR) to 25% (SW), representing relative declines of 35%–68% from the random‐CV CIs reported above.

## 4 DISCUSSION

### 4.1 Automated phenotyping reveals phenotypic variation in seed traits

Seed size traits are strongly correlated among themselves and with seed weight, a major yield‐contributing component, factors influencing processing efficiency, and farmer and consumer preferences (Skylas et al., 2025; Yu et al., 2023). Large diversity panels can serve as valuable sources of genotypes exhibiting broad variation in seed size and weight. Therefore, exploring and understanding the genetic architecture of these traits is essential for genomics‐assisted mungbean improvement. This study demonstrates, for the first time in mungbean, that image‐based automated seed phenotyping can reliably extract seed dimensional traits, providing the precise measurements required for downstream genetic analyses. The stronger concordance between image‐based dimensions and Vernier caliper measurements (*r* > 0.91, *p* < 0.001) confirms the precision and reliability of our automated high‐throughput phenotyping pipeline. Furthermore, the image‐derived dimensional traits showed strong correlations (*r* = 0.91–0.94, *p* < 0.001) with manually recorded SWT. This demonstrates that the two‐dimensional digital extraction accurately captures the primary drivers of physical seed mass, a pattern consistent with high‐throughput image analysis previously reported in crops such as soybean and sorghum (J. Kim et al., 2025; Shrestha et al., 2025). The estimated larger heritabilities (*H* ^2^) of these traits could indicate their potential role in genomics‐assisted crop improvement (Alemu et al., 2024; Ferrão et al., 2024). Ultimately, this validates that the SAM‐based approach can successfully replace laborious manual measurements without sacrificing quantitative accuracy.

Furthermore, the high heritability and prediction accuracies observed in this study highlight the advantage of using deep learning models over traditional computer vision techniques. While threshold‐based methods are often sensitive to seed coat pigmentation, potentially leading to the exclusion or mis‐segmentation of non‐green (e.g., black, yellow, or mottled) genotypes, the SAM framework relies on geometric and textural features rather than color intensity (Kirillov et al., 2023; Osco et al., 2023). This zero‐shot capability ensured that phenotypic data remained robust across the diverse morphological variations present in the IMD panel, thereby minimizing error and maximizing the power of downstream genomic analyses (J. Li et al., 2025; R. Wang et al., 2025; Xiao et al., 2022).

### 4.2 GWAS and candidate genes for seed traits

This study used the IMD panel to perform a multi‐model GWAS with BLINK, FarmCPU, MLM, and SVEN to identify QTLs associated with seed size, shape, and weight traits. A greater overlap of QTLs among the four GWAS models was observed for these traits, consistent with previous studies (Chiteri et al., 2023; Rairdin et al., 2022; Van der Laan et al., 2025). From the combined‐environment data analysis, we identified 82 unique significant markers associated with the seven seed traits.

Approximately 13 MTAs were associated with more than three traits, and several of these overlapped with previously reported mungbean loci. Notably, A marker on chromosome 6, 5_21173402, was associated with SL, SA, SPM, and SWT, and SNPs near these markers have also been reported in a multi‐environment GWAS to be associated with pod width (Boddepalli, et al., 2026a), SWT, and seed yield (Iqbal et al., 2025; Lin et al., 2025; Manjunatha et al., 2023). Another marker, 5_35265704, located on chromosome 4, associated with SL, SAR, SCR, and SWT in our study, was previously linked to SL (J. Liu et al., 2022), pod length (Boddepalli et al., 2026a; Manjunatha et al., 2023), pod width, pod curvature (Boddepalli et al., 2026a), and seed weight (X. Han et al., 2024). Similarly, a region on chromosome 9 with SNP 9_13495720, which was associated with SA, SW, SPM, and SWT in this study, was previously reported to be associated with pod width and SWT (Boddepalli et al., 2026a; X. Han et al., 2024). The overlap between these genomic regions and those reported in earlier mungbean studies strengthens the reliability of our findings and suggests that these loci may harbor candidate genes underlying variation in seed traits. In addition to these previously reported QTLs, we detected several novel loci with relatively larger PVE values that associated with seed size, shape, and weight traits. For instance, markers 1_10969052 and 2_14494712 on chromosome 1, and 8_36270211 on chromosome 10 were consistently associated with multiple seed size and weight traits with high PVE, providing new insights into the genetic architecture of seed traits in mungbean. We prioritized candidate‐gene discussion for loci with cross‐model replication (identified by ≥2 of the four GWAS models); 8_36270211, despite its high PVE, was detected by SVEN alone and is therefore not carried forward for comparative mapping pending independent replication.

### 4.3 Comparative mapping of candidate genes

Comparative mapping of mungbean candidate genes identified through GWAS revealed strong evolutionary conservation with orthologs across multiple legume species and *Arabidopsis*, supporting their functional relevance in seed development. BLASTp analysis detected high‐confidence orthologs (*E*‐value = 0) across soybean, adzuki bean, cowpea, and common bean, indicating the conservation of these protein‐coding regions and their potential roles in regulating seed traits. For example, *Virad06G0255600*, harboring the major‐effect SNP 5_21173402 associated with multiple seed traits, showed strong conservation with orthologous genes in cowpea, adzuki bean, and common bean, as well as with several soybean paralogs. Its closest *Arabidopsis* ortholog, AT1G27460 (NPGR1, NO POLLEN GERMINATION RELATED 1), encodes a calmodulin‐binding protein expressed in pollen, suspension culture cells, flowers, and fruits, and is characterized primarily for its essential role in pollen germination and tube growth (Golovkin & Reddy, 2003). Also, the soybean paralogs, *Glyma.04G045800* and *Glyma.06G046600* were previously reported for their involvement in seed development (Duan et al., 2023; Liang et al., 2024). While *Virad06G0255600* is the primary candidate due to the physical location of the significant SNP, interestingly, *Virad06G0255800*, located approximately 12.4 kb downstream, is annotated as a RING‐type E3 ubiquitin‐protein ligase known for regulating seed and organ size in *Arabidopsis* (Du et al., 2014; Xia et al., 2013) has also been previously reported in mungbean (Lin et al., 2025) for seed size traits. Its soybean ortholog, *Glyma.06G046500* (RING/FYVE/PHD zinc finger superfamily protein; IPR013083, IPR016181), is located on chromosome 6 in close physical proximity to *Glyma.06G046600*, the soybean paralog of *Virad06G0255600* cited above, suggesting both mungbean candidates lie within a syntenically conserved genomic block across *Vign*a and *Glycine*. Given the stronger direct functional precedent for RING‐type zinc finger proteins in seed‐size regulation, *Virad06G0255800* may represent a comparably or more parsimonious candidate than *Virad06G0255600* for this locus; we present both as hypothesis‐generating candidates pending fine‐mapping or functional validation.

Similarly, *Virad01G0084400* gene, with SNP 2_14494712, has been shown to have strong conservation across legumes and its seed‐specific expression in the *Arabidopsis* ortholog *AT3G28345*. This gene encodes an ABC transporter family protein, which is widely implicated in metabolite transport, hormone signaling, and seed filling in plants (Banasiak & Jasiński, 2022; Guo et al., 2022; Mall et al., 2023; Y. Zhang et al., 2025). Another strong candidate, *Virad03G0002200*, harbored the SNP 6_36577242, which encodes a serine/threonine protein kinase homologous to *AT1G20650* (ASG5), a gene known to regulate seed germination and early growth (Baudouin et al., 2022; Qiu et al., 2024; Tomita et al., 2021). Similarly, a strong candidate, *Virad04G0076700*, was associated with the SNP 5_35265704, which encodes an ERF. This gene is likely involved in the hormonal regulation of seed development, as ERFs are known to integrate ethylene and ABA signaling to modulate seed size and biomass accumulation (Z. Li et al., 2022). The strong cross‐species conservation of these genes, together with their annotated molecular functions in protein transport, kinase signaling, chromatin modification, hormone metabolism, and cell wall organization, suggests that they represent biologically meaningful loci underlying variation in mungbean seed traits.

### 4.4 GP of seed traits in mungbean

We assessed the GPA for the seed size, shape, and weight. The quantitative inheritance of these traits makes GP a valuable tool for accelerating the improvement of complex traits in mungbean breeding programs. To date, no GP reports have been published on mungbean seed traits, except for a few on pod morphological traits (Boddepalli et al., 2026a) and leaf water content traits (Iqbal et al., 2025). Among the assessed traits, SL, SW, SA, SWT, and SPM exhibited the highest accuracies (0.76–0.84), consistent with their relatively higher heritabilities (0.96–0.98). Seed shape traits showed lower GPAs (SAR: *r* = 0.46; SCR: *r* = 0.32) despite high heritability (SAR: *H* ^2^ = 0.91; SCR: *H* ^2^ = 0.88), so heritability does not explain the weaker predictability. Both traits instead have the narrowest phenotypic ranges in the panel (CV = 4.23% and 0.89%, respectively; Table 2), even with a high proportion of genetic variance, leaving very little absolute variation to predict when the range is this constrained. Seed shape traits, particularly SCR, may thus be less amenable to genomic selection in this panel, not for lack of a genetic basis, but for lack of absolute phenotypic spread. Incorporating significant SNPs identified from GWAS into the prediction model resulted in slight but consistent improvements across all traits, a pattern previously reported in mungbean (Iqbal et al., 2025). This small magnitude of improvement could be due to the polygenic background effect dominating the genetic control of seed traits in mungbean.

The evaluation of selection coincidence provided additional insights into the practical utility of GP for breeding applications. The high coincidence (up to 80%) between the top 20 selection candidates based on GEBVs and phenotypic BLUEs for SWT suggests that genomic selection could effectively identify superior genotypes for this trait. Interestingly, the gBLUP + SNPs model resulted in lower selection coincidence for most traits, suggesting that while incorporating major‐effect SNPs as fixed covariates improves overall model fit, it can lead to re‐ranking of elite individuals, potentially reducing the efficiency of identifying the absolute top‐performing genotypes for certain seed shape and size parameters. These coincidence estimates should be interpreted cautiously, as they are inherently sensitive to the small selection fraction evaluated (top 20 of 368 genotypes, ∼5%) and to the underlying population structure of the diversity panel; small shifts in ranking near the selection threshold can produce disproportionately large swings in the CI.

We observed a substantial decline in accuracy under LOCO relative to random CV indicates that our random‐CV estimates reflect a best‐case scenario in which test genotypes share close relatives with the training set, realistic for predicting additional individuals within this panel, but optimistic for genetically distant germplasm. Therefore, gBLUP using genome‐wide markers is sufficiently robust for within‐panel selection and for incorporating closely related germplasm into breeding efforts, consistent with prior use of diverse panels for pre‐breeding (Alemu et al., 2024), but accuracy is expected to decline substantially for more distantly related material, and independent validation within the target germplasm pool is recommended before deployment. Notably, the relative predictability of traits, size and weight traits outperforming shape traits, was robust to validation scheme, suggesting this ranking reflects genuine differences in genetic architecture rather than an artifact of population structure.

## 5 CONCLUSION

This study pioneered the integration of the SAM‐based deep learning model with multi‐model GWAS, comparative genomics, and GP to precisely dissect the phenotypic and genetic architecture underlying the seed traits in the IMD panel. The zero‐shot capability of the phenotyping pipeline enabled precise trait extraction across diverse seed coat colors, overcoming the limitations of traditional color‐thresholding methods. This approach enabled the identification of both previously reported and novel QTLs, including robust, large‐effect loci that withstood rigorous bootstrap validation. While comparative mapping of the identified candidate genes revealed strong conservation across other legumes and *Arabidopsis*, we emphasize that these remain hypothesis‐generating association candidates pending validation in independent populations or functional follow‐up. Furthermore, while incorporating significant SNPs into the GP framework provided marginal gains, the results demonstrate that the standard gBLUP model is sufficiently robust and resource‐efficient for practical breeding applications. Collectively, the findings from this study deepen our understanding of the genetic architecture of mungbean seed traits and provide validated predictive tools and target regions to accelerate genomics‐assisted breeding for improved seed yield.

## AUTHOR CONTRIBUTIONS

**Venkata Naresh Boddepalli**: Conceptualization; data curation; formal analysis; investigation; methodology; software; validation; visualization; writing—original draft; writing—review and editing. **Talukder Zaki Jubery**: Methodology; software; writing—review and editing. **Steven B. Cannon**: Methodology; writing—review and editing. **Andrew Farmer**: Writing—review and editing. **Somak Dutta**: Formal analysis; methodology; writing—review and editing. **Baskar Ganapathysubramanian**: Conceptualization; methodology; software; writing—review and editing. **Arti Singh**: Conceptualization; funding acquisition; methodology; project administration; supervision; writing—original draft; writing—review and editing.

## CONFLICT OF INTEREST STATEMENT

The authors declare no conflicts of interest.

## DECLARATION OF GENERATIVE AI AND AI‐ASSISTED TECHNOLOGIES

During the preparation of this manuscript, the authors utilized Gemini (Google) to assist with language editing, structural formatting, and improving the overall readability of the text. The AI was not used to generate, alter, or manipulate any original research data, figures, or scientific results. After using this tool, the authors rigorously reviewed and edited the content to ensure scientific accuracy and take full responsibility for the final publication.

## Supporting information



Supplementary Material

## Data Availability

The data and computational pipelines supporting the findings of this study are publicly available to ensure full reproducibility. Data, including raw phenotypic traits, best linear unbiased estimators (BLUEs), GWAS results from four models, and detailed GP results, are provided in the Supporting Information accompanying this article. The mungbean genotypic dataset utilized in this study is publicly accessible through the Legume Information System (LIS) at https://data.legumeinfo.org/Vigna/radiata/genomes/Weilv‐9.gnm1.5TZZ/. The custom Python scripts, Segment Anything Model (SAM) configurations, and the complete image processing pipeline used for automated trait extraction are open‐source and available at https://github.com/baskargroup/MungSeedAI/tree/master. Additionally, all R scripts utilized for descriptive statistics, multi‐model GWAS, and genomic prediction analyses are version‐controlled and publicly accessible at https://github.com/vboddepalli89/Integrative‐Genomics‐and‐Deep‐Learning‐Based‐Phenotyping‐Reveal‐the‐Genetic‐Architecture‐of‐Seed‐Tra.
