# Supplementary material for: Integrative genomics and zero‐shot deep learning–based phenotyping reveal the genetic architecture of seed traits in mungbean (Vigna radiata L.)
Source: Plant Genome. 2026 Aug 19;19(3):e70294. doi: 10.1002/tpg2.70294 (PMC13492609; doi:10.1002/tpg2.70294)
Supplement: Supplementary file 1 [file TPG2-19-e70294-s001.pdf]

Table S1. Ground-truth validation dataset: SAM-extracted and manual caliper measurements of seed length and width for 166 seeds spanning four seed-coat colors.

Seed Coat Color	Seed Number	SAM-Extracted Seed Length (mm)	SAM-Extracted Seed Width (mm)	Manual Caliper Seed Length (mm)	Manual Caliper Seed Width (mm)
Green	1	5.6	3.91	5.3	3.51
Green	2	5.56	4.03	5.12	3.49
Green	3	5.33	3.89	5.08	3.73
Green	4	5.29	3.99	4.88	3.92
Green	5	5.67	3.97	5.04	3.94
Green	7	5.95	4.33	5.75	4.01
Green	8	5.22	4.11	4.9	3.8
Green	9	5.37	3.89	5.08	3.52
Green	10	5.52	3.85	5.38	3.66
Green	12	5.4	3.72	5.22	3.55
Green	13	6.03	4.33	5.8	4.07
Green	15	5.71	3.85	5.57	3.71
Green	16	5.34	3.9	5.02	3.62
Green	17	5.06	3.91	4.69	3.18
Green	18	5.81	3.98	5.33	3.45
Green	19	5.47	3.96	5.24	3.7
Green	20	5.58	3.85	5.46	3.6
Green	21	5.05	3.83	4.69	3.33
Green	22	5.34	3.88	5.11	3.59
Green	23	5.51	3.9	5.35	3.61
Green	24	4.76	3.55	4.45	2.79
Green	25	4.88	3.63	4.69	2.74
Green	26	5.4	4.12	5.06	4.02
Green	27	5.16	3.76	4.92	3.52
Green	28	5.79	3.85	5.65	3.77
Green	29	4.84	3.54	4.73	3.33
Green	30	5.59	4.21	5.54	3.75
Green	31	5.61	4	5.23	3.65
Green	32	4.98	3.87	4.79	3.54
Green	33	5.36	4.16	5.02	3.59
Green	34	5.66	4.14	5.32	3.91
Green	35	5.6	4.13	5.44	3.81
Green	36	5.09	3.75	5.28	4.12
Green	37	5.31	3.89	5.02	3.49
Green	38	5.62	4.09	5.44	4.05
Green	39	5.05	4.07	4.62	3.2
Green	40	5.64	3.83	5.47	3.74
Green	41	5.72	3.98	5.51	3.57
Green	42	5.21	3.68	5.06	3.56
Green	43	5.22	3.81	4.96	3.45
Green	44	5.6	4.13	5.43	3.58
Green	45	5.42	4.13	4.79	3.4
Green	47	5.45	4.1	5.19	4.03
Green	48	5.82	4.28	5.63	4.07
Green	49	5.05	3.81	4.91	3.62
Green	50	5.18	3.63	4.98	3.24
Green	51	5.44	3.78	5.26	3.58
Green	52	4.94	3.54	4.68	3.02

Seed Coat Color	Seed Number	SAM-Extracted Seed Length (mm)	SAM-Extracted Seed Width (mm)	Manual Caliper Seed Length (mm)	Manual Caliper Seed Width (mm)
Green	53	5.56	3.56	5.24	2.98
Green	54	5	3.86	4.81	3.5
Green	55	5.51	3.85	5.22	3.64
Green	56	5.9	4.21	5.82	3.89
Green	57	5.7	4.08	5.5	4.01
Green	58	5.17	4	4.99	3.72
Green	59	5.44	3.72	5.27	3.63
Green	60	5.47	4.02	5.19	3.61
Yellow	1	4.83	3.95	4.38	3.58
Yellow	2	5.01	4.11	4.78	3.98
Yellow	3	5.19	4.11	4.95	3.65
Yellow	4	4.92	4.02	4.38	3.59
Yellow	6	5.28	4.1	4.87	3.83
Yellow	7	4.62	3.67	3.99	3.8
Yellow	9	5.22	4.31	4.81	3.88
Yellow	10	4.5	3.69	4.2	3.58
Yellow	12	4.85	4.09	4.57	3.66
Yellow	13	5.16	4.15	4.8	4.05
Yellow	14	5.11	3.99	4.75	3.97
Yellow	15	5.22	3.88	5.18	3.79
Yellow	17	5.03	3.94	4.72	3.63
Yellow	19	4.59	4.04	4.15	3.72
Yellow	20	4.96	3.65	4.89	3.4
Yellow	21	4.82	4.15	4.32	3.92
Yellow	22	4.42	3.74	4.04	3.62
Yellow	24	4.47	3.49	4.07	3.31
Yellow	25	5.13	3.95	4.72	3.58
Yellow	26	4.72	3.81	4.43	3.87
Yellow	27	5.29	3.9	4.92	3.61
Yellow	30	4.65	3.94	4.72	3.87
Yellow	31	5.11	4.38	4.64	4.09
Yellow	34	5.09	4.02	4.68	3.68
Yellow	35	4.85	3.92	4.34	3.57
Yellow	36	5.53	4.26	5.11	3.95
Yellow	37	5.51	4.16	5.23	3.84
Yellow	38	5.28	4.26	4.7	4.04
Yellow	39	4.69	4.1	4.28	3.67
Yellow	43	4.92	3.93	4.6	3.75
Yellow	44	5	4.2	4.36	3.54
Yellow	45	4.59	3.54	4.44	3.36
Yellow	46	4.93	3.92	4.45	3.66
Yellow	47	4.43	3.69	4.13	3.56
Yellow	48	4.47	4.02	4.18	3.77
Yellow	49	5.12	4.38	4.73	4.14
Yellow	50	4.95	4.11	4.76	3.9
Yellow	53	5	4.36	4.39	3.86
Yellow	56	4.67	4.37	4.19	3.65
Yellow	57	4.14	3.79	3.82	3.65
Yellow	58	5.04	4.05	5.05	3.92
Yellow	59	5.1	3.89	4.87	3.66

Seed Coat Color	Seed Number	SAM-Extracted Seed Length (mm)	SAM-Extracted Seed Width (mm)	Manual Caliper Seed Length (mm)	Manual Caliper Seed Width (mm)
Mottled	1	4.07	3.36	3.7	2.94
Mottled	2	4.39	2.97	4.08	2.73
Mottled	3	4.16	3.19	3.75	2.84
Mottled	4	4.49	3.5	4.12	3
Mottled	5	4.25	3.6	3.64	2.91
Mottled	7	4.56	3.9	3.97	3.27
Mottled	8	3.99	3.08	3.57	2.72
Mottled	9	3.78	3.37	3.4	3.01
Mottled	10	4.41	3.32	4.05	2.94
Mottled	11	4.01	3.16	3.78	2.77
Mottled	12	3.88	3.32	3.51	2.95
Mottled	13	4.11	3.42	3.61	2.75
Mottled	14	4.27	3.28	3.79	2.91
Mottled	17	4.33	3.56	3.9	3.05
Mottled	18	4.4	3.31	3.88	2.98
Mottled	19	4.25	3.4	3.91	2.93
Mottled	20	3.8	3.35	3.46	3.12
Mottled	21	3.81	2.88	3.36	2.51
Mottled	22	3.83	3.1	3.73	2.84
Mottled	23	4.1	3.04	3.68	2.8
Mottled	24	4.31	3.24	3.95	2.97
Mottled	25	4.05	3.27	3.54	2.81
Mottled	26	4.14	3.25	3.97	2.89
Mottled	29	4.04	3.31	3.61	3.05
Mottled	30	4.13	3.15	3.85	2.81
Mottled	31	4.41	3.21	4.28	3.02
Mottled	32	4.46	3.11	4.24	2.87
Mottled	33	4.15	3.22	3.95	2.96
Mottled	34	3.98	3.24	3.7	2.9
Mottled	39	4.68	3.58	4.2	3.02
Mottled	40	4.49	3.03	4.07	2.82
Mottled	41	3.98	3.4	3.54	3.09
Mottled	42	3.66	3.24	3.3	2.99
Mottled	43	5.02	3.18	4.66	3.01
Mottled	44	3.78	3.32	3.47	2.97
Mottled	45	4.49	3.25	4.34	2.9
Mottled	49	3.93	3.22	3.68	2.86
Mottled	50	3.94	3.15	3.67	2.76
Mottled	52	4.55	3.43	4.13	3.22
Mottled	53	3.61	3.02	3.28	2.76
Mottled	54	4.46	3.36	4.16	2.92
Mottled	59	3.94	3.05	3.72	2.67
Mottled	60	3.85	3.09	3.53	2.9
Black	1	4.45	3.61	4.13	3.24
Black	2	4.35	3.49	4.04	3.11
Black	10	3.8	2.93	3.62	2.75
Black	12	3.64	2.98	3.39	2.77
Black	14	4.43	3.45	4.09	3.18
Black	18	4.34	3.34	4.18	3.22
Black	19	4.41	3.44	4.22	3.36

Seed Coat Color	Seed Number	SAM-Extracted Seed Length (mm)	SAM-Extracted Seed Width (mm)	Manual Caliper Seed Length (mm)	Manual Caliper Seed Width (mm)
Black	20	3.82	3.21	3.54	2.95
Black	23	4.06	3.36	3.5	2.99
Black	28	4.58	3.22	4.29	3.1
Black	29	4.07	3.5	3.73	3.25
Black	30	4.13	3.11	3.87	2.93
Black	32	3.84	2.84	3.52	2.63
Black	33	4.02	3.12	3.83	2.94
Black	38	3.69	3.14	3.57	2.78
Black	39	4.1	3.22	3.86	3.05
Black	40	3.93	2.94	3.69	2.77
Black	41	4.09	3.08	3.67	2.89
Black	43	3.98	3.22	3.76	2.96
Black	48	3.92	3.65	3.6	3.32
Black	50	4.02	2.84	3.84	2.62
Black	52	4.15	3.26	3.9	2.94
Black	56	4.34	3.43	4.12	2.99
Black	59	3.86	3.37	3.52	3.09
Black	60	3.83	3.25	3.49	2.85

Table S2. Best linear unbiased estimates (BLUEs) for seven seed traits across 372 genotypes of the Iowa Mungbean Diversity (IMD) panel.

Genotype	Seed Area (mm^2)	Seed Length (mm)	Seed Width (mm)	Aspect Ratio	Seed Perimeter (mm)	Seed Circularity	100-Seed Weight (g)
AVMU0001	9.38	3.83	3.13	1.23	10.96	0.98	2.73
AVMU0201	11.77	4.38	3.43	1.28	12.3	0.972	4.49
AVMU8501	12.9	4.65	3.53	1.32	12.91	0.967	4.18
AVMU09701	13.18	4.63	3.6	1.29	12.97	0.972	5.37
Berken	12.98	4.53	3.68	1.23	12.92	0.976	4.83
CommChk1	11.82	4.39	3.43	1.28	12.34	0.972	3.33
CommChk2	13.98	4.8	3.71	1.29	13.43	0.971	5.25
Lincon	13.12	4.54	3.68	1.23	12.94	0.977	4.88
OK2000	13.36	4.64	3.66	1.27	13.09	0.975	4.94
PI163110	10.55	4.1	3.29	1.24	11.64	0.978	3.28
PI164726	12	4.37	3.52	1.24	12.43	0.974	3.7
PI164770	11.05	4.23	3.33	1.27	11.92	0.971	3.07
PI167295	10.67	4.09	3.32	1.23	11.68	0.98	3.33
PI179703	11.09	4.09	3.46	1.18	11.87	0.986	3.62
PI183458	13.06	4.58	3.65	1.25	12.97	0.975	4.13
PI183936	10.95	4.1	3.39	1.21	11.8	0.983	3.77
PI199355	11.23	4.25	3.37	1.26	12.02	0.973	3.08
PI201869	16.03	5.29	3.86	1.37	14.48	0.96	5.62
PI201874	11.19	4.2	3.4	1.23	11.98	0.978	3.6
PI213012	12.23	4.4	3.54	1.25	12.51	0.979	3.55
PI254557	10.1	4.05	3.19	1.27	11.41	0.973	2.91
PI264686	13.86	4.67	3.78	1.24	13.31	0.98	5.74
PI271401	15.58	4.96	4	1.24	14.12	0.975	6.03
PI271403	10.59	4.04	3.34	1.21	11.62	0.983	3.16
PI305413	10.85	4.14	3.35	1.24	11.79	0.98	3.39
PI346313	10.55	4.04	3.34	1.21	11.61	0.98	2.83
PI346315	12.79	4.53	3.6	1.26	12.81	0.977	4.2
PI362307	11.16	4.18	3.4	1.23	11.94	0.983	3.46
PI362309	11.84	4.31	3.5	1.23	12.3	0.98	3.48
PI362310	11.75	4.26	3.5	1.22	12.23	0.983	3.83
PI362311	11.08	4.07	3.47	1.17	11.86	0.988	3.58
PI362316	10.79	4.05	3.4	1.19	11.73	0.984	3.42
PI362322	12.07	4.41	3.49	1.26	12.46	0.976	4.01
PI362324	11.74	4.29	3.49	1.23	12.26	0.979	3.46
PI362326	11.78	4.25	3.54	1.20	12.25	0.983	3.89
PI362327	11.59	4.29	3.44	1.25	12.18	0.981	3.67
PI363147	9.98	3.97	3.21	1.24	11.31	0.977	2.77
PI363149	10.65	4.13	3.32	1.24	11.75	0.978	2.98
PI363152	9.9	3.94	3.2	1.23	11.26	0.977	2.75
PI363153	11.62	4.39	3.35	1.31	12.22	0.969	3.5
PI363190	11.63	4.28	3.47	1.24	12.21	0.98	3.89
PI363204	12.33	4.43	3.55	1.25	12.58	0.976	3.67
PI363222	9.81	3.87	3.23	1.20	11.19	0.983	2.88
PI363231	10.47	4.01	3.33	1.21	11.55	0.982	2.95
PI363271	9.93	3.97	3.2	1.24	11.3	0.975	3.12
PI363272	11.02	4.19	3.35	1.25	11.88	0.979	3.14
PI363301	11.16	4.19	3.24	1.29	11.91	0.976	2.69
PI363302	10.38	4.15	3.19	1.30	11.58	0.971	3.17
PI363315	9.88	3.95	3.21	1.23	11.28	0.973	2.94
PI363324	12.96	4.49	3.66	1.23	12.83	0.981	3.95

Genotype	Seed Area (mm²)	Seed Length (mm)	Seed Width (mm)	Aspect Ratio	Seed Perimeter (mm)	Seed Circularity	100-Seed Weight (g)
PI363326	8.99	3.78	3.06	1.24	10.77	0.974	2.89
PI363330	10.02	3.94	3.25	1.21	11.32	0.981	3
PI363332	10.45	4.12	3.25	1.27	11.61	0.978	2.92
PI363333	10.36	4.02	3.28	1.23	11.51	0.98	3.21
PI363334	11.74	4.39	3.42	1.28	12.31	0.97	3.16
PI363341	12.62	4.59	3.38	1.36	12.66	0.942	3.29
PI363346	10.07	4.03	3.2	1.26	11.39	0.977	2.63
PI363354	11.18	4.28	3.31	1.29	11.98	0.972	2.73
PI363358	14.13	4.91	3.66	1.34	13.54	0.964	4.91
PI363359	12.3	4.32	3.63	1.19	12.51	0.984	4.28
PI363360	15.69	4.97	4.02	1.24	14.16	0.977	5.16
PI363362	14.35	4.81	3.81	1.26	13.59	0.977	5
PI363374	10.62	4.09	3.27	1.25	11.59	0.973	2.76
PI363377	10.81	4.13	3.33	1.24	11.75	0.977	2.95
PI363380	10.62	4.14	3.27	1.26	11.68	0.976	2.97
PI363381	14.57	4.86	3.82	1.27	13.69	0.974	5.48
PI363391	10.25	4.03	3.24	1.24	11.45	0.976	2.43
PI363407	10.73	4.15	3.3	1.26	11.75	0.977	3.43
PI363416	12.61	4.51	3.57	1.26	12.74	0.971	4.04
PI363419	12.13	4.53	3.42	1.33	12.54	0.967	3.86
PI363459	10.16	4.02	3.22	1.25	11.41	0.979	2.94
PI363474	11.93	4.3	3.53	1.22	12.33	0.984	3.29
PI363475	9.05	3.76	3.05	1.23	10.73	0.979	2.23
PI363484	10.18	3.85	3.37	1.14	11.36	0.99	3.04
PI363485	10.85	4.09	3.39	1.20	11.77	0.983	3.34
PI363493	10.32	4	3.3	1.21	11.49	0.982	3.03
PI363494	10.29	3.98	3.29	1.21	11.46	0.982	3
PI363506	11.21	4.13	3.47	1.19	11.95	0.986	3.41
PI363514	12.71	4.6	3.53	1.30	12.82	0.968	4.17
PI363516	10.68	4.1	3.32	1.24	11.69	0.979	2.98
PI363517	11.9	4.35	3.51	1.24	12.37	0.982	4.29
PI363523	13.9	4.63	3.81	1.21	13.29	0.981	5.6
PI363565	9.96	4.02	3.15	1.28	11.31	0.975	2.5
PI363566	9.56	3.85	3.17	1.22	11.04	0.983	2.68
PI363585	9.88	3.91	3.2	1.22	11.2	0.981	2.62
PI363591	10.31	4.17	3.16	1.32	11.57	0.969	2.7
PI363592	10.72	4.02	3.4	1.18	11.67	0.985	3.1
PI363595	10.81	4.1	3.37	1.22	11.76	0.98	3.13
PI363596	9.39	3.77	3.19	1.18	10.94	0.979	2.92
PI363597	9.72	4.02	3.08	1.30	11.21	0.972	2.66
PI363600	10.6	4.11	3.31	1.24	11.67	0.978	3.07
PI363604	11.75	4.34	3.46	1.26	12.28	0.977	3.76
PI363605	10.44	4.04	3.28	1.23	11.53	0.98	2.33
PI363613	11.94	4.32	3.55	1.22	12.39	0.975	4.34
PI363615	11.92	4.36	3.48	1.25	12.37	0.976	3.86
PI363635	10.79	4.21	3.27	1.29	11.79	0.973	3.02
PI363645	11.16	4.26	3.35	1.27	11.99	0.975	3.44
PI363647	10.24	3.89	3.35	1.16	11.4	0.988	3.01
PI363657	10.13	4.01	3.24	1.24	11.42	0.978	2.89
PI363668	10.41	4.11	3.23	1.27	11.57	0.971	2.48
PI363678	10.27	4.02	3.25	1.23	11.45	0.981	3.15
PI363696	9.89	3.99	3.16	1.26	11.27	0.975	2.93

Genotype	Seed Area (mm²)	Seed Length (mm)	Seed Width (mm)	Aspect Ratio	Seed Perimeter (mm)	Seed Circularity	100-Seed Weight (g)
PI363699	12.54	4.39	3.64	1.21	12.65	0.983	4.1
PI363763	14.08	4.82	3.72	1.30	13.47	0.973	4.45
PI363766	11.75	4.34	3.44	1.26	12.27	0.976	3.53
PI363778	11.04	4.23	3.33	1.27	11.92	0.975	3.8
PI363779	12	4.42	3.46	1.28	12.42	0.975	3.43
PI363781	9.56	3.92	3.14	1.25	11.12	0.971	2.69
PI363782	10.32	4.05	3.26	1.24	11.53	0.974	3.06
PI363820	9.16	3.76	2.97	1.27	10.61	0.975	1.65
PI363823	9.23	3.86	3.06	1.26	10.91	0.971	2.61
PI363824	9.06	3.74	3.01	1.24	10.63	0.981	1.69
PI363825	8.91	3.68	3.08	1.19	10.64	0.988	2.28
PI363828	10.6	4.08	3.32	1.23	11.66	0.978	3.1
PI363836	11.38	4.23	3.42	1.24	12.06	0.98	3.15
PI363840	10.22	4.01	3.25	1.24	11.44	0.981	2.86
PI363869	10.61	4.02	3.34	1.21	11.58	0.98	3.61
PI363909	11.74	4.27	3.46	1.24	12.29	0.974	3.71
PI363974	13.29	4.73	3.6	1.32	13.15	0.963	4.26
PI363988	10.4	4.08	3.25	1.26	11.55	0.975	3.13
PI363989	10.12	4.03	3.22	1.25	11.43	0.973	3.01
PI364007	11.88	4.38	3.47	1.26	12.37	0.975	3.43
PI364015	10.96	4.2	3.34	1.26	11.88	0.974	3.07
PI364016	12	4.37	3.5	1.25	12.41	0.977	3.29
PI364028	9.52	3.82	3.18	1.20	11.03	0.98	2.99
PI364047	11.3	4.35	3.33	1.31	12.12	0.967	3.37
PI364049	11.84	4.31	3.5	1.23	12.3	0.981	3.86
PI364070	10.46	4.1	3.28	1.25	11.64	0.973	3.35
PI364119	12.29	4.46	3.5	1.27	12.54	0.972	3.38
PI364123	10.49	4.12	3.27	1.26	11.66	0.969	3
PI364167	10.8	4.16	3.29	1.26	11.74	0.973	2.63
PI364205	13.11	4.48	3.74	1.20	12.94	0.981	4.51
PI364213	11.99	4.37	3.54	1.23	12.46	0.974	4.18
PI364214	10.5	4.17	3.24	1.29	11.7	0.969	3.08
PI364218	10.07	4.18	3.19	1.31	11.38	0.975	2.91
PI364224	11.14	4.17	3.42	1.22	11.95	0.978	3.41
PI364225	10.94	4.14	3.37	1.23	11.83	0.98	3.17
PI364230	10.78	4.21	3.26	1.29	11.79	0.966	2.63
PI364231	9.65	4.01	3.06	1.31	11.16	0.97	2.72
PI368266	10.72	4.14	3.3	1.26	11.73	0.976	2.98
PI368275	8.51	3.65	2.97	1.23	10.44	0.979	2.12
PI368277	11.72	4.37	3.41	1.28	12.26	0.974	3.19
PI368279	14.45	4.79	3.86	1.24	13.63	0.971	5.37
PI368285	9.78	3.99	3.13	1.27	11.23	0.969	2.69
PI368302	11.66	4.34	3.45	1.26	12.26	0.972	3.55
PI368305	9.61	3.9	3.15	1.24	11.11	0.973	2.65
PI368329	11	4.22	3.33	1.27	11.89	0.977	3.12
PI368330	11.72	4.27	3.51	1.22	12.25	0.981	3.87
PI368332	9.74	4	3.11	1.29	11.21	0.971	2.61
PI369768	14.58	4.87	3.81	1.28	13.69	0.973	5.46
PI369795	13.78	4.76	3.68	1.29	13.31	0.972	4.99
PI374130	10.86	4.18	3.31	1.26	11.82	0.976	3.1
PI376849	10.98	4.27	3.28	1.30	11.91	0.971	3.25
PI376850	12.33	4.45	3.53	1.26	12.58	0.979	3.8

Genotype	Seed Area (mm^2)	Seed Length (mm)	Seed Width (mm)	Aspect Ratio	Seed Perimeter (mm)	Seed Circularity	100-Seed Weight (g)
PI376856	12.02	4.34	3.52	1.23	12.38	0.981	4.34
PI376923	15.69	5.16	3.84	1.34	14.22	0.965	6.41
PI376951	8.89	3.71	3.05	1.22	10.65	0.981	2.23
PI376952	8.28	3.65	2.92	1.25	10.35	0.974	1.88
PI376989	10.25	4.05	3.18	1.27	11.4	0.972	2.28
PI377014	8.94	3.76	3.03	1.24	10.71	0.978	2.08
PI377022	11.41	4.2	3.47	1.21	12.08	0.982	3.58
PI377039	10.3	4.07	3.21	1.27	11.47	0.974	2.78
PI377081	10.03	3.99	3.21	1.24	11.34	0.978	2.97
PI377138	13	4.59	3.57	1.29	12.87	0.972	3.92
PI377147	11.71	4.31	3.48	1.24	12.27	0.976	3.79
PI377186	9.63	3.87	3.17	1.22	11.1	0.98	2.76
PI377193	11.06	4.28	3.34	1.28	12.02	0.97	3.21
PI377195	11.76	4.37	3.44	1.27	12.31	0.974	3.75
PI377196	11.07	4.23	3.34	1.27	11.93	0.977	3.49
PI377197	11.21	4.29	3.37	1.27	12.09	0.972	3.4
PI377198	10.96	4.25	3.29	1.29	11.89	0.972	3.3
PI377202	10.24	4.06	3.21	1.26	11.48	0.975	2.77
PI377203	10.11	3.92	3.27	1.20	11.33	0.981	2.63
PI377207	10.71	4.27	3.2	1.34	11.8	0.965	3.08
PI377208	10.84	4.14	3.34	1.24	11.78	0.978	3.08
PI377217	9.71	3.97	3.12	1.27	11.19	0.974	2.73
PI377220	12.69	4.53	3.58	1.26	12.77	0.975	3.86
PI377223	11.46	4.27	3.42	1.25	12.13	0.975	3.74
PI377228	9.91	3.9	3.25	1.20	11.25	0.981	2.66
PI377231	10.23	4.06	3.23	1.26	11.48	0.975	2.96
PI377232	12.17	4.42	3.51	1.26	12.49	0.977	4.45
PI377233	10.52	4.07	3.29	1.24	11.59	0.981	3.22
PI377235	11.02	4.26	3.3	1.29	11.93	0.972	3.37
PI377236	11.25	4.15	3.44	1.21	11.95	0.982	3.82
PI377238	10.19	4.06	3.23	1.26	11.49	0.974	2.86
PI377239	11.93	4.35	3.51	1.24	12.37	0.979	3.77
PI377241	10.61	4.11	3.31	1.24	11.68	0.977	3.15
PI377242	11	4.12	3.41	1.21	11.85	0.982	3.16
PI377244	12.16	4.39	3.54	1.24	12.49	0.98	3.79
PI377248	11.98	4.37	3.47	1.26	12.35	0.976	3.27
PI377249	11.08	4.19	3.36	1.25	11.91	0.979	3.04
PI377250	10.93	4.16	3.35	1.24	11.85	0.976	3.33
PI377255	10.54	3.98	3.38	1.18	11.58	0.987	3.3
PI377256	9.94	3.95	3.21	1.23	11.27	0.98	2.94
PI377258	11.31	4.25	3.37	1.26	12	0.977	3.04
PI377259	10.2	4.06	3.21	1.27	11.45	0.974	3.26
PI377261	10.57	4.09	3.31	1.24	11.65	0.978	3.35
PI377262	11.85	4.32	3.5	1.24	12.31	0.98	3.56
PI377263	10.44	4.11	3.24	1.27	11.59	0.973	2.68
PI377265	9.15	5.05	2.77	1.82	12.95	0.893	2.63
PI377266	10.73	4.13	3.29	1.26	11.7	0.976	2.88
PI377267	9.46	3.88	3.12	1.24	11.03	0.975	2.64
PI377268	10.3	4.1	3.22	1.27	11.54	0.97	2.97
PI377271	9.76	4	3.12	1.28	11.22	0.974	2.83
PI377272	9.89	3.99	3.14	1.27	11.25	0.977	2.88
PI377274	10.15	4.02	3.17	1.27	11.32	0.975	2.87

Genotype	Seed Area (mm²)	Seed Length (mm)	Seed Width (mm)	Aspect Ratio	Seed Perimeter (mm)	Seed Circularity	100-Seed Weight (g)
PI377275	9.25	3.83	3.08	1.24	10.89	0.979	2.89
PI377276	10.99	4.21	3.33	1.26	11.89	0.975	3.35
PI377281	10.67	4.08	3.34	1.22	11.69	0.98	3.28
PI377283	10.21	4.01	3.25	1.24	11.44	0.98	3.16
PI377284	11.18	4.27	3.32	1.29	11.97	0.972	2.88
PI377286	10.42	4.03	3.29	1.23	11.53	0.982	3.24
PI377287	10.9	4.12	3.36	1.23	11.78	0.978	2.89
PI377288	10.66	4.16	3.27	1.27	11.71	0.976	3.1
PI377296	10.27	4.07	3.2	1.27	11.47	0.977	2.77
PI377297	10.51	4.11	3.27	1.26	11.62	0.975	2.77
PI377298	9.43	3.84	3.14	1.22	10.99	0.98	2.68
PI377299	10.49	3.98	3.36	1.18	11.56	0.985	3.09
PI377304	11.14	4.23	3.36	1.26	11.97	0.975	3.52
PI377305	9.91	3.94	3.18	1.24	11.23	0.978	2.36
PI377306	11.16	4.19	3.4	1.23	11.95	0.978	3.04
PI377312	9.61	3.94	3.12	1.26	11.13	0.974	2.59
PI377313	10.2	4.06	3.19	1.27	11.43	0.973	2.5
PI377316	11.41	4.33	3.33	1.30	12.09	0.969	2.74
PI377338	10.15	3.94	3.29	1.20	11.37	0.984	3.34
PI377342	10.23	3.96	3.29	1.20	11.41	0.985	2.94
PI377349	10.19	4.11	3.17	1.29	11.47	0.969	2.76
PI377354	11.01	4.12	3.42	1.20	11.87	0.981	3.66
PI377363	12	4.45	3.43	1.30	12.43	0.972	3.93
PI385297	9.95	3.96	3.2	1.24	11.29	0.98	3.08
PI390419	14.96	5	3.82	1.31	13.91	0.968	5.73
PI413223	10.68	4.15	3.3	1.26	11.74	0.971	2.94
PI424964	12.05	4.46	3.43	1.30	12.43	0.972	4.45
PI424984	15.29	5	3.89	1.29	14.03	0.974	6.28
PI425029	11.44	4.27	3.42	1.25	12.12	0.977	3.4
PI425038	13.49	4.65	3.7	1.26	13.15	0.976	5.08
PI425045	15.06	4.82	3.81	1.27	13.59	0.974	5.54
PI425085	15.4	5	3.92	1.28	14.07	0.972	5.51
PI425130	11.27	4.2	3.43	1.22	12.02	0.977	3.88
PI425136	9.79	3.89	3.21	1.21	11.17	0.985	2.81
PI425138	11.54	4.28	3.43	1.25	12.16	0.978	3.56
PI425160	14.49	4.88	3.77	1.29	13.64	0.971	5.48
PI425167	15.65	5.18	3.85	1.34	14.26	0.962	6.09
PI425169	10.56	4.11	3.27	1.26	11.63	0.973	2.88
PI425192	9.17	3.79	3.1	1.22	10.85	0.978	2.54
PI425201	13.79	4.69	3.75	1.25	13.3	0.978	4.85
PI425219	10.7	4.15	3.28	1.26	11.72	0.977	3.39
PI425229	16.57	5.19	4.08	1.27	14.62	0.973	6.43
PI425240	15.58	5.1	3.87	1.32	14.16	0.969	6.32
PI425254	10.23	3.97	3.28	1.21	11.4	0.981	2.73
PI425259	11.8	4.29	3.5	1.22	12.28	0.98	3.42
PI425261	11.25	4.16	3.44	1.21	11.98	0.982	4.08
PI425264	10.43	4.19	3.21	1.30	11.69	0.963	2.8
PI425269	14.1	4.85	3.7	1.31	13.49	0.967	5.78
PI425271	10.9	4.22	3.3	1.28	11.86	0.972	3.08
PI425274	11.11	4.23	3.35	1.26	11.94	0.975	3
PI425330	10.27	3.99	3.31	1.21	11.5	0.974	3.46
PI425385	9.82	3.92	3.2	1.23	11.21	0.98	2.82

Genotype	Seed Area (mm²)	Seed Length (mm)	Seed Width (mm)	Aspect Ratio	Seed Perimeter (mm)	Seed Circularity	100-Seed Weight (g)
PI425387	9.73	3.93	3.17	1.24	11.18	0.977	2.77
PI425388	9.84	3.92	3.19	1.23	11.21	0.982	2.86
PI425389	9.59	3.91	3.14	1.25	11.11	0.975	3.06
PI425399	11.22	4.38	3.27	1.34	12.08	0.965	3.5
PI425413	12.5	4.5	3.55	1.27	12.68	0.973	3.54
PI425418	11.68	4.35	3.42	1.27	12.26	0.975	3.57
PI425421	11.06	4.16	3.39	1.23	11.9	0.98	3.45
PI425422	11.55	4.25	3.48	1.22	12.16	0.98	3.62
PI425423	10.24	4.02	3.25	1.24	11.46	0.978	2.89
PI425424	10.48	4.05	3.31	1.22	11.58	0.979	3.05
PI425425	13.63	4.81	3.61	1.33	13.29	0.964	4.95
PI425435	10.4	3.99	3.34	1.19	11.55	0.985	3.11
PI425446	9.71	3.97	3.14	1.26	11.18	0.976	2.85
PI425456	10.97	4.26	3.27	1.30	11.89	0.972	3.43
PI425463	14.13	4.92	3.68	1.34	13.57	0.963	4.54
PI425464	13.94	4.8	3.7	1.30	13.4	0.97	5.08
PI425481	10.48	4.08	3.27	1.25	11.6	0.976	3.61
PI425483	12.93	4.48	3.63	1.23	12.9	0.977	3.98
PI425484	10.36	4.1	3.24	1.26	11.56	0.974	3.02
PI425485	10.14	4.03	3.21	1.26	11.43	0.976	2.83
PI425487	10.18	4.01	3.24	1.24	11.42	0.98	2.69
PI425503	10.95	4.18	3.33	1.25	11.83	0.978	3.19
PI425504	11.75	4.33	3.45	1.26	12.26	0.976	3.61
PI425506	10.24	4.07	3.2	1.27	11.47	0.974	2.72
PI425507	10.51	4.12	3.23	1.27	11.59	0.977	3.03
PI425508	9.85	3.98	3.18	1.25	11.22	0.978	2.7
PI425509	9.62	3.94	3.12	1.26	11.12	0.975	2.79
PI425510	11.97	4.38	3.44	1.27	12.42	0.969	2.98
PI425511	10.36	4.06	3.27	1.24	11.54	0.973	3.02
PI425512	11.56	4.29	3.45	1.24	12.19	0.976	3.83
PI425513	13.32	4.74	3.59	1.32	13.14	0.966	4.7
PI425515	14.18	4.87	3.72	1.31	13.56	0.967	4.4
PI425517	13.01	4.54	3.66	1.24	12.92	0.979	4.49
PI425518	12.83	4.53	3.62	1.25	12.85	0.976	4.5
PI425520	11.13	4.29	3.32	1.29	12	0.972	3.62
PI425522	11.24	4.15	3.45	1.20	11.96	0.984	3.58
PI425523	13.4	4.62	3.7	1.25	13.12	0.975	5.22
PI425527	12.89	4.53	3.62	1.25	12.85	0.978	4.56
PI425528	11.21	4.21	3.4	1.24	11.98	0.98	3.75
PI425529	10.21	3.97	3.28	1.21	11.43	0.983	3.05
PI425533	11.82	4.3	3.51	1.23	12.3	0.982	3.85
PI425534	9.87	3.98	3.16	1.26	11.26	0.976	2.96
PI425535	10.07	4.06	3.17	1.28	11.4	0.973	2.85
PI425536	12.12	4.46	3.47	1.29	12.5	0.973	3.97
PI425537	10.41	4.02	3.29	1.22	11.52	0.984	3.22
PI425544	13.16	4.58	3.66	1.25	12.98	0.975	4.77
PI425546	10.36	4.01	3.3	1.22	11.51	0.981	2.97
PI425548	11.62	4.25	3.5	1.21	12.2	0.979	3.43
PI425551	14.64	4.85	3.85	1.26	13.72	0.976	5.57
PI425552	11.78	4.22	3.56	1.19	12.24	0.987	3.91
PI425554	11.39	4.19	3.48	1.21	12.07	0.982	3.66
PI425557	11.48	4.2	3.48	1.20	12.09	0.984	3.77

Genotype	Seed Area (mm²)	Seed Length (mm)	Seed Width (mm)	Aspect Ratio	Seed Perimeter (mm)	Seed Circularity	100-Seed Weight (g)
PI425569	12.84	4.56	3.61	1.26	12.88	0.974	4.33
PI425570	11.53	4.2	3.5	1.20	12.12	0.984	3.78
PI425573	11.16	4.18	3.43	1.22	11.97	0.979	3.8
PI425575	12.27	4.45	3.52	1.26	12.56	0.976	3.92
PI425577	10.85	4.12	3.35	1.23	11.76	0.982	3.35
PI425580	10.91	4.08	3.42	1.19	11.8	0.984	3.45
PI425581	11.35	4.19	3.45	1.21	12.02	0.984	3.77
PI425582	12.17	4.34	3.58	1.21	12.47	0.982	3.9
PI425583	12.69	4.48	3.61	1.24	12.74	0.977	4.2
PI425585	11.68	4.37	3.4	1.28	12.26	0.974	3.93
PI425586	10.91	4.1	3.38	1.21	11.78	0.985	3.54
PI425587	13.07	4.55	3.67	1.24	12.95	0.977	4.52
PI425591	12.57	4.37	3.68	1.19	12.66	0.984	4.08
PI425592	13.16	4.54	3.69	1.23	12.96	0.979	4.91
PI425593	14.21	4.83	3.74	1.29	13.52	0.97	4.9
PI425594	14.79	4.82	3.91	1.23	13.75	0.979	5.55
PI425599	14.26	4.84	3.78	1.28	13.58	0.969	5.28
PI425610	11.79	4.38	3.44	1.27	12.33	0.97	3.81
PI425634	12.09	4.49	3.46	1.30	12.54	0.97	3.84
PI425646	14.18	4.8	3.75	1.28	13.48	0.973	4.86
PI425725	13.91	4.72	3.75	1.26	13.38	0.975	5.06
PI425765	11.22	4.31	3.36	1.28	12.08	0.972	3.72
PI425766	11.04	4.23	3.33	1.27	11.92	0.975	3.21
PI425770	10.09	3.94	3.28	1.20	11.36	0.981	2.93
PI425771	10.45	4.07	3.28	1.24	11.59	0.978	3.24
PI425803	12.66	4.53	3.56	1.27	12.76	0.973	4.27
PI425811	11.01	4.16	3.36	1.24	11.85	0.983	3.27
PI425812	12.82	4.5	3.63	1.24	12.8	0.977	4.7
PI425814	14.39	4.87	3.79	1.28	13.65	0.97	4.84
PI425815	11.86	4.31	3.51	1.23	12.33	0.978	4.32
PI425816	17.8	5.59	4.06	1.38	15.26	0.959	7.03
PI425818	12.33	4.36	3.59	1.22	12.52	0.981	4.53
PI425822	9.74	3.87	3.22	1.20	11.17	0.982	2.87
PI425823	9.16	3.8	3.09	1.23	10.85	0.976	2.54
PI425824	9.78	3.99	3.16	1.26	11.27	0.97	2.73
PI425832	10.87	4.18	3.31	1.26	11.81	0.973	2.98
PI425840	9.99	3.98	3.21	1.24	11.32	0.978	2.82
PI425841	10.58	4.1	3.29	1.25	11.65	0.977	3.02
PI425842	10.03	3.97	3.22	1.23	11.32	0.98	3.01
PI425852	11.11	4.26	3.32	1.28	11.95	0.974	3.09
PI425859	15.5	4.93	4.01	1.23	14.09	0.978	6.23
PI425862	13.63	4.7	3.7	1.27	13.24	0.975	4.45
PI425891	12.57	4.39	3.65	1.20	12.66	0.984	4.16
PI425965	11.56	4.28	3.41	1.26	12.12	0.978	3.06
PI426026	12.36	4.55	3.49	1.30	12.67	0.97	4.06
PI426029	13.04	4.57	3.61	1.27	12.9	0.976	4.56
PI426038	12.84	4.56	3.59	1.27	12.85	0.974	4.69
PI426039	14.87	4.94	3.83	1.29	13.83	0.971	5.74
PI426042	13.65	4.67	3.74	1.25	13.25	0.969	5.39
PI426055	11.32	4.3	3.37	1.28	12.1	0.97	3.61
PI426058	9.99	4.03	3.16	1.28	11.33	0.971	2.68
PI426098	10.59	4.11	3.3	1.25	11.67	0.975	3.25

Genotype	Seed Area (mm^2)	Seed Length (mm)	Seed Width (mm)	Aspect Ratio	Seed Perimeter (mm)	Seed Circularity	100-Seed Weight (g)
PI426109	9.29	3.82	3.09	1.24	10.9	0.977	2.94
PI426118	10.77	4.16	3.3	1.26	11.75	0.976	2.73
PI426127	10.53	4.05	3.32	1.22	11.6	0.982	3.34
PI426139	10.56	4.05	3.32	1.22	11.6	0.98	3
PI426149	9.79	3.98	3.15	1.26	11.25	0.972	2.83
PI427024	9.33	3.94	3.03	1.30	11	0.968	2.41
PI427060	13.11	4.57	3.63	1.26	12.92	0.975	4.92
PI674166	14.29	4.8	3.78	1.27	13.52	0.976	5.04
TypeP	14.51	5.03	3.69	1.36	13.79	0.961	4.39
TypeRB	11.26	4.34	3.3	1.32	12.05	0.971	3.04

Table S3. Genomic estimated breeding values (GEBVs) for seven seed traits from genomic prediction (gBLUP) across 368 genotypes.

Genotype	Seed Area (mm^2)	Seed Length (mm)	Seed Width (mm)	Aspect Ratio	Seed Perimeter (mm)	Seed Circularity	100-Seed Weight (g)
AVMU0001	10.29	4.19	3.25	1.29	11.59	0.973	3.38
AVMU0201	11.4	4.32	3.34	1.29	11.86	0.973	4.17
AVMU8501	13.55	4.79	3.62	1.32	13.16	0.972	4.89
AVMU09701	11.83	4.41	3.42	1.29	12.11	0.968	4.45
Berken	13.53	4.71	3.71	1.27	13.21	0.972	5.02
CommChk1	11.4	4.31	3.4	1.27	12.13	0.977	3.32
CommChk2	13.09	4.66	3.64	1.28	13.07	0.977	4.54
Lincon	13.34	4.54	3.7	1.23	13.15	0.977	4.78
OK2000	13.47	4.79	3.62	1.32	13.22	0.972	5.16
PI163110	11.11	4.26	3.37	1.26	12.04	0.977	3.49
PI164726	12.1	4.31	3.52	1.22	12.4	0.978	3.77
PI164770	11.36	4.18	3.39	1.23	12.03	0.976	3.4
PI167295	10.73	4.1	3.32	1.23	11.7	0.977	3.33
PI179703	11.73	4.27	3.52	1.21	12.22	0.978	3.79
PI183458	12.77	4.55	3.62	1.26	12.91	0.978	4.16
PI183936	10.47	4.08	3.32	1.23	11.57	0.978	3.3
PI199355	11.18	4.28	3.38	1.27	12.01	0.977	3.32
PI201869	14.22	4.72	3.77	1.25	13.53	0.976	4.96
PI201874	11.28	4.27	3.39	1.26	11.99	0.977	3.66
PI213012	12.05	4.24	3.53	1.20	12.48	0.978	3.65
PI254557	10.58	4.2	3.24	1.30	11.68	0.976	3.16
PI264686	14.37	4.76	3.78	1.26	13.43	0.973	5.59
PI271401	14.29	4.71	3.86	1.22	13.49	0.977	5.2
PI271403	10.6	4.03	3.33	1.21	11.65	0.978	3.2
PI305413	11.56	4.33	3.39	1.28	12.17	0.977	3.7
PI346313	10.17	3.94	3.25	1.21	11.3	0.973	2.87
PI346315	12.26	4.42	3.56	1.24	12.54	0.977	4.07
PI362307	11.82	4.3	3.49	1.23	12.32	0.979	3.62
PI362309	11.61	4.3	3.49	1.23	12.16	0.978	3.52
PI362310	12.3	4.36	3.51	1.24	12.54	0.978	3.68
PI362311	11.29	4.17	3.45	1.21	12.02	0.979	3.67
PI362316	11.18	4.14	3.42	1.21	11.95	0.978	3.6
PI362322	11.62	4.31	3.44	1.25	12.16	0.978	3.87
PI362324	11.49	4.33	3.44	1.26	12.16	0.978	3.58
PI362326	11.78	4.32	3.5	1.23	12.23	0.979	3.88
PI362327	12.19	4.41	3.53	1.25	12.46	0.978	3.89
PI363147	10.72	4.18	3.29	1.27	11.75	0.976	3.03
PI363149	10.68	4.21	3.32	1.27	11.73	0.977	3.1
PI363152	10.59	4.03	3.28	1.23	11.62	0.977	3.03
PI363153	11.73	4.4	3.4	1.29	12.31	0.975	3.61
PI363190	11.51	4.26	3.45	1.23	12.09	0.977	3.68
PI363204	12.33	4.37	3.56	1.23	12.54	0.978	3.85
PI363222	10.18	4.01	3.25	1.23	11.41	0.977	3.09
PI363231	10.49	4.1	3.3	1.24	11.58	0.977	3.07
PI363271	10.11	3.96	3.2	1.24	11.37	0.976	2.92
PI363272	11.15	4.19	3.37	1.24	11.95	0.976	3.17
PI363301	10.74	4.14	3.26	1.27	11.69	0.977	2.93
PI363302	10.48	4.11	3.23	1.27	11.65	0.976	3.1
PI363315	10.15	3.99	3.24	1.23	11.4	0.976	2.94
PI363324	14.02	4.68	3.81	1.23	13.35	0.977	4.96

Genotype	Seed Area (mm^2)	Seed Length (mm)	Seed Width (mm)	Aspect Ratio	Seed Perimeter (mm)	Seed Circularity	100-Seed Weight (g)
PI363326	9.43	3.89	3.09	1.26	11.01	0.968	2.74
PI363330	10.84	4.12	3.29	1.25	11.72	0.977	3.33
PI363332	10.36	3.98	3.22	1.24	11.5	0.977	2.68
PI363333	10.43	4.07	3.27	1.24	11.58	0.977	3.16
PI363334	11.21	4.24	3.4	1.25	12.02	0.976	3.16
PI363341	11.45	4.25	3.33	1.28	12.09	0.965	3.2
PI363346	10.1	4.15	3.19	1.30	11.48	0.976	2.71
PI363354	10.45	4.14	3.24	1.28	11.61	0.975	2.62
PI363358	11.88	4.37	3.47	1.26	12.29	0.977	3.75
PI363359	12.76	4.47	3.68	1.21	12.8	0.978	4.44
PI363360	14.18	4.7	3.84	1.22	13.43	0.977	5.12
PI363362	13.63	4.74	3.7	1.28	13.33	0.977	4.77
PI363374	10.16	3.96	3.24	1.22	11.35	0.977	2.74
PI363377	10.67	4.13	3.29	1.26	11.7	0.976	3.02
PI363380	10.57	4.14	3.26	1.27	11.67	0.976	2.85
PI363381	14.09	4.72	3.77	1.25	13.6	0.977	5.14
PI363391	10.22	4.03	3.21	1.26	11.41	0.976	2.54
PI363407	10.92	4.12	3.31	1.24	11.77	0.977	3.44
PI363416	12.83	4.52	3.64	1.24	12.8	0.978	4.36
PI363419	11.63	4.36	3.39	1.29	12.22	0.976	3.65
PI363459	10.36	4.08	3.24	1.26	11.53	0.976	2.89
PI363474	11.68	4.26	3.45	1.23	12.2	0.977	3.42
PI363475	9.88	3.99	3.16	1.26	11.19	0.977	2.67
PI363484	10.14	4.02	3.24	1.24	11.41	0.977	2.86
PI363485	11.93	4.28	3.5	1.22	12.23	0.977	3.74
PI363493	10.97	4.24	3.38	1.25	11.82	0.978	3.34
PI363494	10.14	4.05	3.25	1.25	11.47	0.977	2.94
PI363506	11.3	4.12	3.44	1.20	12.03	0.978	3.29
PI363514	12.59	4.37	3.53	1.24	12.63	0.976	4.04
PI363516	10.87	4.18	3.34	1.25	11.82	0.976	3.11
PI363517	12.15	4.36	3.54	1.23	12.56	0.978	4.1
PI363523	13.05	4.53	3.69	1.23	12.92	0.978	4.77
PI363565	9.94	3.97	3.17	1.25	11.3	0.977	2.59
PI363566	9.92	3.95	3.19	1.24	11.26	0.977	2.69
PI363585	9.95	3.99	3.19	1.25	11.4	0.977	2.71
PI363591	10.48	4.17	3.22	1.30	11.64	0.975	2.8
PI363592	11.83	4.32	3.49	1.24	12.38	0.977	3.54
PI363595	10.84	4.19	3.36	1.25	11.9	0.977	3.27
PI363596	10.44	4.16	3.27	1.27	11.55	0.976	3.23
PI363597	9.87	3.99	3.14	1.27	11.28	0.977	2.76
PI363600	10.71	4.14	3.3	1.25	11.74	0.976	3.09
PI363604	11.56	4.18	3.4	1.23	12.21	0.977	3.48
PI363605	10.29	4.06	3.22	1.26	11.54	0.976	3.12
PI363613	11.1	4.24	3.41	1.24	12	0.976	3.7
PI363615	11.81	4.28	3.48	1.23	12.26	0.977	3.61
PI363635	10.74	4.17	3.27	1.28	11.79	0.976	3.03
PI363645	12.06	4.3	3.47	1.24	12.33	0.978	3.87
PI363647	11.29	4.16	3.41	1.22	12.02	0.977	3.27
PI363657	10.2	4.01	3.23	1.24	11.43	0.977	2.85
PI363668	10.25	4.05	3.22	1.26	11.51	0.976	2.67
PI363678	10.37	4.13	3.24	1.27	11.53	0.976	3.15
PI363696	10.22	4.12	3.21	1.28	11.45	0.976	3.08

Genotype	Seed Area (mm²)	Seed Length (mm)	Seed Width (mm)	Aspect Ratio	Seed Perimeter (mm)	Seed Circularity	100-Seed Weight (g)
PI363699	12.67	4.48	3.65	1.23	12.76	0.978	4.21
PI363763	13.34	4.62	3.69	1.25	13.03	0.977	4.56
PI363766	11.77	4.32	3.45	1.25	12.28	0.976	3.43
PI363778	10.94	4.15	3.33	1.25	11.86	0.968	3.54
PI363779	11.44	4.37	3.39	1.29	12.1	0.976	3.35
PI363781	9.95	4	3.18	1.26	11.28	0.976	2.87
PI363782	10.37	4.12	3.26	1.26	11.55	0.975	2.94
PI363820	9.19	3.83	3.03	1.26	10.82	0.977	2.04
PI363823	9.57	3.93	3.1	1.27	11.06	0.976	2.48
PI363824	9.12	3.82	3.03	1.26	10.77	0.977	1.99
PI363825	10.28	4	3.25	1.23	11.47	0.978	2.97
PI363828	10.45	4.08	3.27	1.25	11.63	0.976	3.02
PI363836	11.71	4.33	3.47	1.25	12.3	0.978	3.39
PI363840	10.48	4.1	3.27	1.25	11.57	0.976	2.85
PI363869	10.65	4	3.32	1.20	11.54	0.977	3.48
PI363909	11.91	4.29	3.48	1.23	12.33	0.976	3.69
PI363974	12.08	4.43	3.48	1.27	12.45	0.975	3.78
PI363988	10.49	4.09	3.26	1.25	11.64	0.976	2.99
PI363989	10.16	4	3.23	1.24	11.44	0.968	3.02
PI364015	10.64	4.12	3.31	1.24	11.73	0.976	2.98
PI364016	11.14	4.18	3.39	1.23	11.87	0.976	3.11
PI364028	10.03	3.96	3.23	1.23	11.33	0.973	3.04
PI364047	10.81	4.21	3.31	1.27	11.85	0.976	3.22
PI364049	10.52	4.17	3.3	1.26	11.64	0.977	2.96
PI364070	10.26	4.13	3.24	1.27	11.53	0.976	3.07
PI364119	11.54	4.29	3.44	1.25	12.32	0.967	3.36
PI364123	10.62	4.12	3.28	1.26	11.69	0.972	3.07
PI364167	10.28	4.02	3.22	1.25	11.47	0.977	2.7
PI364205	12.97	4.47	3.68	1.21	12.89	0.977	4.31
PI364213	13	4.42	3.64	1.21	12.86	0.977	4.48
PI364214	10.29	4.15	3.24	1.28	11.56	0.975	2.95
PI364218	10.25	4.17	3.22	1.30	11.5	0.975	3
PI364224	11.53	4.26	3.46	1.23	12.16	0.978	3.42
PI364225	11.28	4.22	3.38	1.25	12.01	0.976	3.2
PI364230	10.55	4.14	3.24	1.28	11.63	0.975	2.71
PI364231	10.84	4.17	3.27	1.28	11.79	0.976	3.24
PI368266	10.76	4.16	3.29	1.26	11.76	0.976	3.07
PI368275	9.3	3.84	3.05	1.26	10.87	0.977	2.15
PI368277	11.19	4.23	3.35	1.26	11.99	0.976	3.16
PI368279	13.69	4.72	3.74	1.26	13.28	0.972	5.08
PI368285	9.76	4.06	3.14	1.29	11.25	0.967	2.69
PI368302	11.23	4.24	3.4	1.25	12.01	0.977	3.47
PI368305	10.45	3.95	3.22	1.23	11.45	0.967	3.01
PI368329	10.89	4.18	3.31	1.26	11.85	0.976	3.07
PI368330	12.51	4.38	3.58	1.22	12.54	0.978	4.14
PI368332	10.03	4.1	3.16	1.30	11.34	0.976	2.76
PI369768	14.9	4.88	3.81	1.28	13.6	0.972	5.73
PI369795	13.78	4.68	3.67	1.28	13.07	0.973	5.13
PI374130	10.99	4.19	3.32	1.26	11.88	0.976	3.16
PI376849	11.12	4.23	3.32	1.27	11.95	0.976	3.23
PI376850	11.66	4.29	3.45	1.24	12.2	0.977	3.68
PI376856	12.09	4.34	3.54	1.23	12.4	0.978	4.04

Genotype	Seed Area (mm²)	Seed Length (mm)	Seed Width (mm)	Aspect Ratio	Seed Perimeter (mm)	Seed Circularity	100-Seed Weight (g)
PI376923	14.89	4.88	3.8	1.28	13.71	0.972	5.79
PI376951	9.54	3.86	3.13	1.23	11.01	0.977	2.46
PI376952	9.29	3.85	3.05	1.26	10.9	0.976	2.15
PI376989	10.19	4.06	3.18	1.28	11.42	0.976	2.38
PI377014	9.18	3.85	3.05	1.26	10.89	0.976	2.11
PI377022	12.12	4.38	3.53	1.24	12.55	0.978	3.87
PI377039	10.11	4.02	3.24	1.24	11.43	0.976	2.99
PI377081	10.13	3.99	3.23	1.24	11.34	0.977	2.85
PI377138	12.5	4.36	3.52	1.24	12.53	0.977	4.05
PI377147	11.74	4.3	3.46	1.24	12.23	0.977	3.76
PI377186	10.35	4.14	3.24	1.28	11.51	0.976	3.03
PI377193	10.84	4.15	3.33	1.25	11.82	0.976	3.03
PI377195	11.02	4.17	3.38	1.23	11.9	0.977	3.35
PI377196	11.59	4.31	3.41	1.26	12.12	0.975	3.8
PI377197	10.67	4.09	3.33	1.23	11.72	0.977	3.14
PI377198	10.73	4.15	3.29	1.26	11.7	0.976	3.11
PI377202	10.19	4.03	3.25	1.24	11.49	0.977	2.81
PI377203	10.29	4.02	3.27	1.23	11.46	0.978	2.83
PI377207	10.86	4.29	3.25	1.32	11.89	0.975	3.19
PI377208	10.81	4.12	3.34	1.23	11.77	0.977	3.27
PI377217	9.73	3.97	3.14	1.26	11.21	0.977	2.71
PI377220	11.71	4.25	3.51	1.21	12.22	0.978	3.57
PI377223	11.32	4.21	3.4	1.24	12.01	0.978	3.6
PI377228	10.65	4.15	3.3	1.26	11.71	0.977	2.97
PI377231	10.33	4.09	3.23	1.27	11.56	0.969	3
PI377232	11.93	4.23	3.47	1.22	12.24	0.977	4.13
PI377233	10.52	4.21	3.27	1.29	11.65	0.974	3.19
PI377235	10.97	4.19	3.32	1.26	11.88	0.976	3.17
PI377236	10.87	4.17	3.37	1.24	11.88	0.977	3.54
PI377238	10.17	4.04	3.2	1.26	11.46	0.975	2.76
PI377239	11.04	4.24	3.39	1.25	11.92	0.977	3.34
PI377241	10.86	4.26	3.31	1.29	11.86	0.977	3.32
PI377242	10.96	4.26	3.41	1.25	11.9	0.978	3.2
PI377244	11.45	4.3	3.46	1.24	12.11	0.978	3.6
PI377248	11.41	4.28	3.42	1.25	12.06	0.978	3.29
PI377249	10.68	4.13	3.3	1.25	11.72	0.976	3.14
PI377250	10.76	4.15	3.31	1.25	11.74	0.976	3.17
PI377255	11.31	4.22	3.45	1.22	11.98	0.979	3.6
PI377256	10.9	4.09	3.31	1.24	11.74	0.977	3.37
PI377258	10.57	4.06	3.28	1.24	11.6	0.977	2.98
PI377259	10.17	4.03	3.22	1.25	11.44	0.977	3.07
PI377261	10.37	4.05	3.28	1.23	11.56	0.977	3.08
PI377262	11.4	4.22	3.45	1.22	12.06	0.977	3.49
PI377263	10.58	4.21	3.27	1.29	11.69	0.976	2.94
PI377265	9.35	4.26	2.95	1.44	11.87	0.963	2.6
PI377266	10.44	4.13	3.26	1.27	11.6	0.977	2.98
PI377267	10.31	4.06	3.22	1.26	11.48	0.976	2.98
PI377268	10.09	4	3.21	1.25	11.39	0.977	2.81
PI377271	9.95	4.06	3.13	1.30	11.35	0.968	2.76
PI377272	10.19	4.11	3.18	1.29	11.56	0.967	2.88
PI377274	10.14	4.04	3.19	1.27	11.53	0.977	2.98
PI377275	9.98	4.01	3.16	1.27	11.25	0.978	2.88

Genotype	Seed Area (mm^2)	Seed Length (mm)	Seed Width (mm)	Aspect Ratio	Seed Perimeter (mm)	Seed Circularity	100-Seed Weight (g)
PI377276	10.55	4.12	3.26	1.26	11.64	0.976	3.13
PI377281	10.27	4.05	3.28	1.23	11.52	0.978	3.09
PI377283	10.58	4.15	3.32	1.25	11.64	0.977	3.2
PI377284	10.56	4.2	3.29	1.28	11.66	0.978	2.92
PI377286	10.49	4.13	3.29	1.26	11.64	0.976	3.18
PI377287	11.07	4.38	3.37	1.30	12.08	0.976	3.16
PI377288	10.34	4.05	3.26	1.24	11.53	0.977	2.99
PI377296	10.38	4.12	3.23	1.28	11.51	0.976	2.82
PI377297	10.13	3.98	3.21	1.24	11.42	0.976	2.69
PI377298	10.07	3.99	3.23	1.24	11.35	0.977	2.94
PI377299	10.39	4.05	3.31	1.22	11.57	0.978	3.1
PI377304	11	4.22	3.38	1.25	11.86	0.977	3.41
PI377305	10.45	4.14	3.25	1.27	11.56	0.977	2.94
PI377306	10.79	4.1	3.36	1.22	11.75	0.978	3.12
PI377312	9.86	3.98	3.17	1.26	11.23	0.977	2.74
PI377313	10.19	4.04	3.21	1.26	11.47	0.976	2.7
PI377316	11.15	4.28	3.33	1.29	12.15	0.976	2.95
PI377338	10.62	3.99	3.31	1.21	11.44	0.978	3.44
PI377342	10.31	3.97	3.28	1.21	11.47	0.978	2.93
PI377349	10.59	4.08	3.22	1.27	11.56	0.968	3.04
PI377354	10.38	4	3.31	1.21	11.5	0.978	3.2
PI377363	11.93	4.48	3.45	1.30	12.57	0.974	3.92
PI385297	9.98	4	3.18	1.26	11.37	0.976	2.89
PI390419	13.96	4.7	3.73	1.26	13.29	0.973	5.26
PI413223	10.28	4.06	3.23	1.26	11.48	0.976	2.88
PI424964	12.73	4.7	3.54	1.33	12.93	0.971	4.54
PI424984	14.77	4.87	3.81	1.28	13.7	0.972	5.76
PI425029	11.93	4.27	3.46	1.23	12.39	0.977	3.72
PI425038	12.25	4.39	3.67	1.20	13.03	0.978	3.66
PI425045	13.86	4.82	3.76	1.28	13.39	0.972	5.1
PI425085	15.15	4.91	3.83	1.28	13.7	0.972	5.89
PI425130	11.67	4.25	3.47	1.22	12.18	0.977	3.81
PI425136	10.16	4	3.22	1.24	11.4	0.977	2.84
PI425138	12.29	4.33	3.5	1.24	12.46	0.977	4.03
PI425160	13.72	4.75	3.72	1.28	13.32	0.972	5.18
PI425167	14.42	5	3.81	1.31	13.66	0.971	5.71
PI425169	10.36	4	3.23	1.24	11.46	0.967	2.94
PI425192	9.78	3.93	3.17	1.24	11.2	0.977	2.65
PI425201	12.2	4.4	3.66	1.20	13	0.977	3.76
PI425219	11.87	4.32	3.4	1.27	12.29	0.977	3.74
PI425229	15.07	4.75	3.89	1.22	13.77	0.977	5.59
PI425240	15.08	4.91	3.84	1.28	13.88	0.972	5.87
PI425254	9.78	3.97	3.2	1.24	11.27	0.977	2.84
PI425259	11.05	4.11	3.41	1.21	11.89	0.977	3.23
PI425261	11.59	4.43	3.43	1.29	12.35	0.977	3.65
PI425264	10.43	4.06	3.24	1.25	11.53	0.977	2.85
PI425269	14.79	4.88	3.78	1.29	13.67	0.972	5.7
PI425271	10.91	4.15	3.34	1.24	11.84	0.977	3.14
PI425274	10.62	4.05	3.29	1.23	11.63	0.976	2.96
PI425330	10.39	4.01	3.31	1.21	11.51	0.977	3.26
PI425385	9.84	3.96	3.18	1.25	11.25	0.977	2.72
PI425387	10.16	4.01	3.21	1.25	11.39	0.976	2.9

Genotype	Seed Area (mm²)	Seed Length (mm)	Seed Width (mm)	Aspect Ratio	Seed Perimeter (mm)	Seed Circularity	100-Seed Weight (g)
PI425388	10.16	4	3.22	1.24	11.4	0.977	2.85
PI425389	9.9	4.08	3.17	1.29	11.3	0.974	3.01
PI425399	11.49	4.3	3.33	1.29	12.1	0.974	3.82
PI425413	11.23	4.32	3.41	1.27	12.2	0.977	3.21
PI425418	11.18	4.23	3.38	1.25	11.94	0.977	3.42
PI425421	11.04	4.26	3.37	1.26	11.95	0.976	3.39
PI425422	11.66	4.21	3.49	1.21	12.26	0.978	3.57
PI425423	11.16	4.11	3.37	1.22	11.92	0.977	3.19
PI425424	10.39	4.07	3.24	1.26	11.59	0.976	3.1
PI425425	14	4.82	3.63	1.33	13.03	0.971	5.41
PI425435	10.88	4.13	3.38	1.22	11.79	0.978	3.45
PI425446	9.98	4.01	3.2	1.25	11.36	0.976	2.84
PI425456	11.68	4.37	3.42	1.28	12.23	0.977	3.83
PI425463	13.06	4.63	3.64	1.27	13.13	0.977	4.2
PI425464	12.81	4.68	3.62	1.29	13	0.976	4.43
PI425481	11.65	4.31	3.4	1.27	12.23	0.977	3.8
PI425483	12.54	4.43	3.6	1.23	12.77	0.978	4.01
PI425484	10.24	4.15	3.19	1.30	11.68	0.974	2.88
PI425485	10.73	4.1	3.28	1.25	11.63	0.978	2.9
PI425487	10.65	4.14	3.29	1.26	11.66	0.977	2.9
PI425503	10.65	4.12	3.28	1.26	11.67	0.976	3.13
PI425504	11.64	4.28	3.46	1.24	12.28	0.978	3.56
PI425506	10.05	4.06	3.16	1.28	11.39	0.968	2.81
PI425507	10.13	4.09	3.17	1.29	11.45	0.968	2.87
PI425508	9.98	4.04	3.18	1.27	11.35	0.968	2.81
PI425509	10.08	4.08	3.16	1.29	11.43	0.968	2.83
PI425510	10.04	3.99	3.15	1.27	11.24	0.978	2.68
PI425511	10.55	4.09	3.3	1.24	11.68	0.977	3.17
PI425512	11.54	4.35	3.46	1.26	12.2	0.977	3.91
PI425513	12.46	4.63	3.57	1.30	12.75	0.973	4.37
PI425515	13.32	4.8	3.68	1.30	13.36	0.972	4.48
PI425517	12.24	4.57	3.55	1.29	12.78	0.977	4.24
PI425518	13.22	4.75	3.71	1.28	13.34	0.974	4.48
PI425520	12.05	4.41	3.45	1.28	12.31	0.977	4.11
PI425522	12.6	4.38	3.62	1.21	12.58	0.978	3.93
PI425523	13.18	4.41	3.68	1.20	12.9	0.976	4.85
PI425527	12.55	4.64	3.58	1.30	12.85	0.976	4.32
PI425528	11.45	4.23	3.43	1.23	12.03	0.977	3.76
PI425529	10.73	4.13	3.34	1.24	11.7	0.977	3.25
PI425533	12.42	4.42	3.59	1.23	12.69	0.978	4.26
PI425534	10.32	4.02	3.24	1.24	11.48	0.977	3.11
PI425535	9.97	4.03	3.17	1.27	11.33	0.977	2.88
PI425536	11.75	4.27	3.46	1.23	12.19	0.977	3.87
PI425537	10.63	4.28	3.32	1.29	12	0.978	3.23
PI425544	13.85	4.73	3.67	1.29	13.1	0.972	5.36
PI425546	11.01	4.23	3.37	1.26	11.87	0.978	3.4
PI425548	11.77	4.32	3.51	1.23	12.24	0.978	3.55
PI425551	13.93	4.65	3.74	1.24	13.13	0.972	5.29
PI425552	12.63	4.41	3.66	1.20	12.78	0.977	4.3
PI425554	12.2	4.4	3.55	1.24	12.61	0.978	4.1
PI425557	11.73	4.25	3.48	1.22	12.24	0.978	3.71
PI425569	13.05	4.46	3.65	1.22	13.01	0.977	4.31

Genotype	Seed Area (mm²)	Seed Length (mm)	Seed Width (mm)	Aspect Ratio	Seed Perimeter (mm)	Seed Circularity	100-Seed Weight (g)
PI425570	11.56	4.31	3.48	1.24	12.16	0.978	3.75
PI425573	11.47	4.29	3.45	1.24	12.09	0.978	3.79
PI425575	11.83	4.33	3.49	1.24	12.28	0.979	3.79
PI425577	11.33	4.28	3.39	1.26	12.01	0.979	3.62
PI425580	10.97	4.1	3.4	1.21	11.85	0.979	3.38
PI425581	11.25	4.27	3.42	1.25	12.01	0.978	3.66
PI425582	11.74	4.29	3.51	1.22	12.2	0.978	3.71
PI425583	11.99	4.39	3.54	1.24	12.37	0.978	4.05
PI425585	11.68	4.34	3.47	1.25	12.21	0.978	3.91
PI425586	11.33	4.22	3.41	1.24	11.98	0.979	3.64
PI425587	12.52	4.43	3.61	1.23	12.66	0.977	4.13
PI425591	12.35	4.56	3.68	1.24	12.81	0.974	4.15
PI425592	13.46	4.46	3.63	1.23	12.82	0.977	4.9
PI425593	14.03	4.7	3.77	1.25	13.53	0.976	4.71
PI425594	13.74	4.6	3.79	1.21	13.32	0.978	4.81
PI425599	14.59	4.81	3.71	1.30	13.4	0.972	5.54
PI425610	12.56	4.51	3.51	1.28	12.8	0.976	4.23
PI425634	12.22	4.55	3.49	1.30	12.58	0.975	4.04
PI425646	13.32	4.62	3.66	1.26	12.92	0.976	4.64
PI425725	13.65	4.63	3.71	1.25	13.18	0.976	4.75
PI425765	11.19	4.21	3.35	1.26	11.86	0.976	3.52
PI425766	11.54	4.46	3.42	1.30	12.21	0.972	3.88
PI425770	10.47	4.07	3.3	1.23	11.56	0.977	3.12
PI425771	10.58	4.18	3.29	1.27	11.62	0.977	3.29
PI425811	11.25	4.25	3.37	1.26	12.01	0.978	3.56
PI425812	12.8	4.39	3.63	1.21	12.7	0.978	4.16
PI425814	13.76	4.65	3.74	1.24	13.38	0.977	4.68
PI425815	12.95	4.41	3.6	1.23	12.99	0.977	4.39
PI425816	14.99	4.89	3.83	1.28	13.81	0.972	5.82
PI425818	13.24	4.71	3.65	1.29	13.24	0.975	4.72
PI425822	10.02	3.99	3.22	1.24	11.37	0.977	2.9
PI425823	9.65	3.96	3.18	1.25	11.22	0.977	2.76
PI425824	11	4.15	3.3	1.26	11.83	0.976	3.31
PI425832	10.24	4.05	3.27	1.24	11.6	0.977	2.83
PI425840	10.41	4.04	3.27	1.24	11.57	0.978	3.08
PI425841	10.43	4.03	3.27	1.23	11.59	0.977	3.05
PI425842	10.31	4.03	3.25	1.24	11.53	0.978	3.04
PI425852	11.17	4.18	3.33	1.26	11.83	0.976	3.24
PI425859	14.05	4.53	3.82	1.19	13.32	0.977	5.06
PI425862	12.92	4.47	3.66	1.22	12.91	0.977	4.17
PI425891	12.27	4.25	3.59	1.18	12.43	0.978	3.86
PI425965	10.79	4.2	3.33	1.26	11.67	0.977	3.09
PI426026	12.8	4.67	3.56	1.31	12.91	0.971	4.49
PI426029	12.15	4.21	3.53	1.19	12.53	0.977	3.83
PI426038	13.71	4.57	3.61	1.27	12.98	0.972	5.12
PI426039	13.79	4.75	3.73	1.27	13.33	0.972	5.23
PI426042	13.78	4.57	3.71	1.23	13.28	0.976	5.09
PI426055	11.1	4.13	3.33	1.24	11.93	0.975	3.23
PI426058	10.07	4.07	3.17	1.28	11.41	0.975	2.77
PI426098	11	4.23	3.34	1.27	11.89	0.977	3.35
PI426109	10.2	3.98	3.19	1.25	11.46	0.976	2.99
PI426118	10.58	4.08	3.28	1.24	11.61	0.977	2.91

Genotype	Seed Area (mm^2)	Seed Length (mm)	Seed Width (mm)	Aspect Ratio	Seed Perimeter (mm)	Seed Circularity	100-Seed Weight (g)
PI426127	10.46	4.06	3.34	1.22	11.61	0.978	3.2
PI426139	10.58	4.07	3.3	1.23	11.63	0.978	3.04
PI426149	9.91	4.03	3.16	1.28	11.34	0.968	2.79
PI427024	9.7	3.98	3.09	1.29	11.17	0.967	2.6
PI427060	13.68	4.64	3.71	1.25	13.2	0.976	4.81
PI674166	13.73	4.64	3.72	1.25	13.22	0.976	4.8

Table S4. Agreement statistics between SAM-extracted and manual caliper measurements for seed length and seed width, overall and stratified by seed-coat color.

Trait	Seed Color	n	r	CCC	Slope	Intercept	Bias (mm)	RMSE (mm)	95% LoA (mm)
**Seed Length**	**Overall**	**166**	**0.978**	**0.884**	**0.927**	**0.632**	**0.306**	**0.336**	**[+0.032, +0.580]**
Seed Length	Green	56	0.91	0.693	0.837	1.09	0.247	0.28	[−0.012, +0.507]
Seed Length	Yellow	42	0.882	0.563	0.793	1.299	0.353	0.388	[+0.035, +0.672]
Seed Length	Mottled	43	0.927	0.559	0.915	0.677	0.354	0.372	[+0.126, +0.582]
Seed Length	Black	25	0.938	0.616	0.906	0.633	0.275	0.29	[+0.095, +0.456]
**Seed Width**	**Overall**	**166**	**0.912**	**0.723**	**0.859**	**0.777**	**0.303**	**0.35**	**[−0.044, +0.649]**
Seed Width	Green	56	0.667	0.349	0.431	2.369	0.317	0.388	[−0.128, +0.761]
Seed Width	Yellow	42	0.686	0.39	0.796	1.023	0.26	0.309	[−0.071, +0.590]
Seed Width	Mottled	43	0.753	0.225	1.006	0.337	0.355	0.376	[+0.110, +0.600]
Seed Width	Black	25	0.904	0.546	0.994	0.271	0.253	0.27	[+0.063, +0.443]

r, Pearson correlation coefficient; CCC, Lin's concordance correlation coefficient; Slope and Intercept, from ordinary least-squares regression of SAM-extracted values on manual caliper values; Bias, mean difference (SAM − Manual); RMSE, root mean square error; 95% LoA, Bland-Altman 95% limits of agreement (bias ± 1.96 SD of the differences). Overall rows (bold) pool all four seed-coat-color genotypes (n = 166); color-specific rows are based on n = 25–56 seeds each. Overall pooled correlations are higher than any individual within-color correlation, reflecting between-genotype size variation rather than stronger true measurement agreement; CCC, which accounts for both correlation and bias, better reflects true within-color agreement.

Table S5. K-means clustering (K = 2) of the Iowa Mungbean Diversity (IMD) panel based on seed trait BLUEs.

Seed Trait	Cluster 1 (n = 288) Mean ± SD	Cluster 2 (n = 83) Mean ± SD	p-value
100-Seed Weight (g)	3.17 ± 0.48	4.89 ± 0.75	<0.001
Seed Length (mm)	4.12 ± 0.17	4.73 ± 0.23	<0.001
Seed Width (mm)	3.31 ± 0.14	3.70 ± 0.15	<0.001
Seed Area (mm²)	10.71 ± 0.83	13.75 ± 1.16	<0.001
Aspect Ratio (L/W)	1.24 ± 0.03	1.28 ± 0.04	<0.001
Seed Perimeter (mm)	11.71 ± 0.46	13.29 ± 0.56	<0.001
Seed Circularity	0.98 ± 0.00	0.97 ± 0.01	<0.001

Welch's two-sample t-test comparing Cluster 1 vs. Cluster 2 for each trait. Cluster 1 corresponds to the small-seeded group; Cluster 2 corresponds to the large-seeded group.

Table S6. Genome-wide association study (GWAS) results for seven seed traits across four models (BLINK, FarmCPU, MLM, SVEN).

Trait	SNP	Chr	Position (bp)	P-value	MAF	Major Allele	Minor Allele	Effect	PVE (%)	SE	Model
Aspect_Ratio	5_35265704	4	7227756	5.86e-6	0.098	T	C	-0.013	9.19	0.003	BLINK
Aspect_Ratio	3_3873913	7	8662388	3.02e-7	0.327	T	C	-0.009	7.74	0.002	BLINK
Aspect_Ratio	7_4544023	8	21967218	1.10e-8	0.054	T	A	-0.023	8.99	0.004	BLINK
Aspect_Ratio	7_32686365	8	61315057	1.85e-6	0.183	G	A	-0.014	0.92	0.004	BLINK
Aspect_Ratio	3_3873913	7	8662388	1.76e-8	0.327	T	C	-0.010	7.74	0.002	FarmCPU
Aspect_Ratio	7_32422349	8	61053530	1.61e-8	0.446	C	A	-0.009	3.29	0.002	FarmCPU
Aspect_Ratio	8_7083954	10	6919926	2.62e-6	0.198	G	A	-0.010	7.92	0.002	FarmCPU
Aspect_Ratio	8_45372529	10	52758894	7.64e-6	0.067	A	G	0.012	5.32	0.004	FarmCPU
Aspect_Ratio	1_28550460	1	13498841	7.63e-1	0.057	T	C	-0.027	2.89	0.008	SVEN
Aspect_Ratio	3_3926418	7	8614977	9.75e-1	0.345	T	A	-0.022	8.45	0.004	SVEN
Aspect_Ratio	7_4544023	8	21967218	5.45e-1	0.054	T	A	-0.047	8.99	0.008	SVEN
Seed_area_mm2	6_36043903	3	1443532	7.85e-6	0.050	C	A	-0.421	10.34	0.178	BLINK
Seed_area_mm2	1_10969052	5	38876574	2.31e-6	0.216	G	A	0.254	3.19	0.112	BLINK
Seed_area_mm2	5_21173402	6	42482684	3.55e-12	0.057	C	A	-0.748	20.71	0.163	BLINK
Seed_area_mm2	scaffold_386_126047	7	40129332	6.44e-9	0.075	C	T	-0.832	27.59	0.195	BLINK
Seed_area_mm2	7_8376596	8	25791292	1.54e-5	0.265	T	C	-0.220	0.06	0.098	BLINK
Seed_area_mm2	7_14737687	8	31133824	8.31e-8	0.091	A	T	-0.401	5.44	0.147	BLINK
Seed_area_mm2	7_32422219	8	61053400	2.38e-7	0.438	C	T	-0.249	20.17	0.076	BLINK
Seed_area_mm2	9_13495720	9	30855923	4.95e-6	0.500	C	T	-0.203	11.89	0.084	BLINK
Seed_area_mm2	8_35612740	10	42997469	9.69e-9	0.102	A	G	-0.475	18.24	0.128	BLINK
Seed_area_mm2	6_23824231	3	17002303	2.51e-6	0.459	A	G	0.259	8.52	0.105	FarmCPU
Seed_area_mm2	5_33130959	4	27275658	8.57e-6	0.075	T	A	0.319	0.52	0.177	FarmCPU
Seed_area_mm2	scaffold_264_199305	6	2064679	6.46e-7	0.333	G	A	-0.330	0.11	0.141	FarmCPU
Seed_area_mm2	5_19686583	6	36281948	1.59e-5	0.073	C	A	-0.329	6.46	0.158	FarmCPU
Seed_area_mm2	scaffold_386_126047	7	40129332	2.61e-7	0.075	C	T	-0.697	27.59	0.195	FarmCPU
Seed_area_mm2	7_43023151	8	7926783	1.79e-5	0.053	G	C	0.399	4.19	0.185	FarmCPU
Seed_area_mm2	7_14737687	8	31133824	2.81e-7	0.091	A	T	-0.318	5.44	0.147	FarmCPU
Seed_area_mm2	7_32422219	8	61053400	8.98e-8	0.438	C	T	-0.242	20.17	0.076	FarmCPU
Seed_area_mm2	7_53591467	8	70674432	1.54e-5	0.120	C	G	0.257	0.01	0.132	FarmCPU
Seed_area_mm2	8_41738423	10	49101348	1.54e-5	0.345	C	G	-0.229	18.92	0.079	FarmCPU
Seed_area_mm2	7_14737687	8	31133824	1.19e-5	0.091	A	T	-0.517	5.44	0.147	MLM
Seed_area_mm2	2_14494712	1	24413902	9.98e-1	0.201	G	A	-2.173	29.30	0.176	SVEN
Seed_area_mm2	scaffold_238_460032	2	26790619	5.70e-1	0.334	C	T	0.880	6.77	0.171	SVEN
Seed_area_mm2	6_6502133	3	38600156	8.30e-1	0.372	A	G	-0.881	7.04	0.167	SVEN
Seed_area_mm2	5_21173402	6	42482684	9.99e-1	0.057	C	A	-3.185	20.71	0.326	SVEN
Seed_area_mm2	7_14737687	8	31133824	9.73e-1	0.087	A	T	-1.360	5.72	0.289	SVEN
Seed_area_mm2	7_32422219	8	61053400	8.53e-1	0.438	C	T	-1.458	20.17	0.152	SVEN
Seed_area_mm2	9_13579483	9	30932950	8.61e-1	0.486	A	C	1.137	12.37	0.158	SVEN
Seed_area_mm2	9_10797185	9	32066020	7.09e-1	0.473	G	C	1.085	11.26	0.159	SVEN

Trait	SNP	Chr	Position (bp)	P-value	MAF	Major Allele	Minor Allele	Effect	PVE (%)	SE	Model
Seed_area_mm2	8_36270211	10	43643353	9.89e-1	0.340	T	C	-1.993	35.25	0.141	SVEN
Seed_circularity	2_15506024	1	26723483	1.69e-9	0.052	C	T	0.003	2.98	0.001	BLINK
Seed_circularity	scaffold_267_414965	1	28773230	3.56e-7	0.058	G	A	-0.006	0.01	0.001	BLINK
Seed_circularity	scaffold_267_414931	1	28773264	2.68e-7	0.056	C	T	-0.006	0.00	0.001	BLINK
Seed_circularity	scaffold_267_351899	1	28835696	6.04e-7	0.060	C	A	-0.006	0.01	0.001	BLINK
Seed_circularity	11_3897518	2	3938167	6.69e-8	0.122	G	A	-0.003	0.39	0.001	BLINK
Seed_circularity	scaffold_174_653757	3	23364798	1.74e-5	0.076	G	A	0.003	3.73	0.001	BLINK
Seed_circularity	7_51266812	8	73856244	1.01e-14	0.057	T	C	0.005	7.83	0.001	BLINK
Seed_circularity	7_51253517	8	73844460	1.46e-5	0.056	T	C	0.004	7.12	0.001	MLM
Seed_circularity	7_51254664	8	73845603	5.28e-6	0.058	C	T	0.005	6.97	0.001	MLM
Seed_circularity	7_51266812	8	73856244	7.66e-6	0.057	T	C	0.004	7.83	0.001	MLM
Seed_circularity	scaffold_149_281864	1	28013950	7.36e-1	0.060	C	T	0.005	2.30	0.002	SVEN
Seed_circularity	scaffold_267_351899	1	28835696	9.83e-1	0.058	C	A	0.000	0.01	0.002	SVEN
Seed_circularity	5_35265704	4	7227756	9.03e-1	0.097	T	C	0.005	3.96	0.001	SVEN
Seed_length_mm	scaffold_386_126047	7	40129332	1.14e-11	0.075	C	T	-0.204	25.86	0.039	BLINK
Seed_length_mm	7_14737687	8	31133824	9.65e-7	0.091	A	T	-0.075	5.21	0.030	BLINK
Seed_length_mm	scaffold_386_126047	7	40129332	8.71e-6	0.075	C	T	-0.131	25.86	0.039	FarmCPU
Seed_length_mm	7_14737687	8	31133824	4.70e-7	0.091	A	T	-0.080	5.21	0.030	FarmCPU
Seed_length_mm	10_16367462	11	4668882	6.65e-6	0.467	A	G	0.039	3.95	0.017	FarmCPU
Seed_length_mm	5_35265704	4	7227756	9.06e-6	0.098	T	C	-0.128	23.65	0.025	MLM
Seed_length_mm	6_36577242	3	267038	8.02e-1	0.182	T	C	0.265	10.12	0.041	SVEN
Seed_length_mm	6_6502133	3	38600156	9.86e-1	0.372	A	G	-0.169	6.43	0.034	SVEN
Seed_length_mm	5_35265704	4	7227756	9.81e-1	0.097	T	C	-0.529	23.65	0.050	SVEN
Seed_length_mm	1_33591256	5	35941149	7.98e-1	0.406	T	C	-0.364	31.77	0.028	SVEN
Seed_length_mm	3_12011523	6	7320433	6.51e-1	0.346	G	A	0.567	18.43	0.062	SVEN
Seed_length_mm	scaffold_386_126047	7	40129332	1.00e+0	0.075	C	T	-0.891	25.86	0.079	SVEN
Seed_length_mm	7_14737687	8	31133824	8.82e-1	0.087	A	T	-0.264	5.37	0.058	SVEN
Seed_length_mm	8_35612740	10	42997469	9.26e-1	0.098	A	G	-0.456	17.65	0.051	SVEN
Seed_perimeter_mm	6_36577242	3	267038	3.29e-6	0.182	T	C	0.126	10.57	0.054	BLINK
Seed_perimeter_mm	5_33732107	4	27865590	1.71e-5	0.276	T	C	0.172	0.91	0.085	BLINK
Seed_perimeter_mm	1_10969052	5	38876574	3.36e-8	0.216	G	A	0.157	3.22	0.058	BLINK
Seed_perimeter_mm	9_13495720	9	30855923	1.82e-5	0.500	C	T	-0.100	11.25	0.044	BLINK
Seed_perimeter_mm	6_2813575	3	42551184	1.61e-5	0.099	C	T	-0.208	0.28	0.127	FarmCPU
Seed_perimeter_mm	5_33130959	4	27275658	6.66e-6	0.075	T	A	0.174	0.53	0.092	FarmCPU
Seed_perimeter_mm	5_24525948	6	45806089	2.17e-6	0.128	G	A	0.147	10.80	0.066	FarmCPU
Seed_perimeter_mm	scaffold_386_126047	7	40129332	9.57e-8	0.075	C	T	-0.376	26.77	0.102	FarmCPU
Seed_perimeter_mm	7_14737687	8	31133824	7.81e-7	0.091	A	T	-0.152	5.29	0.077	FarmCPU
Seed_perimeter_mm	8_34761828	10	42107711	3.34e-7	0.262	T	C	0.190	5.78	0.073	FarmCPU
Seed_perimeter_mm	2_14494712	1	24413902	9.83e-1	0.201	G	A	-1.109	28.12	0.093	SVEN
Seed_perimeter_mm	6_6502133	3	38600156	9.85e-1	0.372	A	G	-0.459	7.04	0.087	SVEN

Trait	SNP	Chr	Position (bp)	P-value	MAF	Major Allele	Minor Allele	Effect	PVE (%)	SE	Model
Seed_perimeter_mm	3_12011610	6	7320346	5.50e-1	0.345	C	A	1.419	16.27	0.168	SVEN
Seed_perimeter_mm	5_21173402	6	42482684	9.96e-1	0.057	C	A	-1.632	20.04	0.170	SVEN
Seed_perimeter_mm	7_14737687	8	31133824	8.28e-1	0.087	A	T	-0.698	5.54	0.150	SVEN
Seed_perimeter_mm	7_32422219	8	61053400	9.63e-1	0.438	C	T	-0.756	19.97	0.079	SVEN
Seed_perimeter_mm	9_13579483	9	30932950	6.33e-1	0.486	A	C	0.578	11.75	0.083	SVEN
Seed_perimeter_mm	9_10955689	9	32259958	8.22e-1	0.287	T	A	0.611	10.60	0.093	SVEN
Seed_perimeter_mm	8_36270211	10	43643353	9.99e-1	0.340	T	C	-1.022	34.13	0.074	SVEN
Seed_width_mm	2_20535168	1	23109130	1.41e-6	0.061	G	A	-0.066	0.04	0.037	BLINK
Seed_width_mm	6_6582096	3	38519889	3.82e-6	0.308	C	T	-0.029	6.07	0.013	BLINK
Seed_width_mm	5_32075306	4	1955759	2.24e-8	0.473	G	A	0.033	0.86	0.012	BLINK
Seed_width_mm	1_10969052	5	38876574	4.57e-7	0.216	G	A	0.029	2.24	0.015	BLINK
Seed_width_mm	scaffold_7_2715854	7	16224248	6.10e-7	0.103	G	A	0.045	8.98	0.018	BLINK
Seed_width_mm	7_32422238	8	61053419	1.92e-5	0.378	T	C	0.028	9.75	0.011	BLINK
Seed_width_mm	9_2000078	9	2361133	3.70e-7	0.340	G	A	0.032	0.11	0.012	BLINK
Seed_width_mm	9_13495720	9	30855923	1.68e-5	0.500	C	T	-0.025	12.83	0.011	BLINK
Seed_width_mm	9_7582889	9	35931388	1.10e-5	0.147	C	T	0.034	0.82	0.017	BLINK
Seed_width_mm	6_23824231	3	17002303	1.41e-5	0.459	A	G	0.031	8.88	0.015	FarmCPU
Seed_width_mm	1_1783223	5	2174677	9.49e-6	0.319	G	A	0.027	12.79	0.012	FarmCPU
Seed_width_mm	1_21989735	5	17175215	2.66e-6	0.212	G	A	-0.032	0.66	0.014	FarmCPU
Seed_width_mm	5_19686583	6	36281948	1.66e-5	0.073	C	A	-0.043	6.80	0.022	FarmCPU
Seed_width_mm	3_9345163	7	3217510	1.32e-5	0.083	A	G	0.051	2.43	0.033	FarmCPU
Seed_width_mm	9_3180813	8	60958922	1.74e-7	0.095	G	C	-0.053	0.08	0.021	FarmCPU
Seed_width_mm	7_53591467	8	70674432	7.93e-6	0.120	C	G	0.037	0.01	0.018	FarmCPU
Seed_width_mm	9_13577167	9	30930634	5.94e-8	0.432	C	A	0.030	9.19	0.012	FarmCPU
Seed_width_mm	scaffold_83_1304075	11	9520499	1.86e-5	0.069	T	A	-0.055	0.00	0.040	FarmCPU
Seed_width_mm	2_14494712	1	24413902	9.18e-1	0.201	G	A	-0.282	26.16	0.025	SVEN
Seed_width_mm	scaffold_238_460032	2	26790619	5.54e-1	0.334	C	T	0.128	7.63	0.023	SVEN
Seed_width_mm	6_6502133	3	38600156	7.13e-1	0.372	A	G	-0.126	7.58	0.023	SVEN
Seed_width_mm	5_15791720	6	37969190	5.94e-1	0.110	A	G	-0.243	11.07	0.036	SVEN
Seed_width_mm	3_7862817	7	4699015	6.60e-1	0.193	T	C	-0.101	3.22	0.029	SVEN
Seed_width_mm	7_14309659	8	30416942	5.98e-1	0.375	G	C	-0.238	27.09	0.020	SVEN
Seed_width_mm	7_32471250	8	61100759	5.61e-1	0.456	C	T	-0.183	17.06	0.021	SVEN
Seed_width_mm	9_13579483	9	30932950	6.08e-1	0.486	A	C	0.162	13.29	0.022	SVEN
Seed_width_mm	9_10797185	9	32066020	6.37e-1	0.473	G	C	0.144	10.47	0.022	SVEN
Seed_width_mm	8_22164297	10	29017031	5.61e-1	0.235	A	G	0.221	18.19	0.025	SVEN
Seed_width_mm	8_36270211	10	43643353	9.90e-1	0.340	T	C	-0.285	38.01	0.019	SVEN
SWT100	1_10969052	5	38876574	7.74e-6	0.216	G	A	0.105	3.02	0.064	BLINK
SWT100	5_19686583	6	36281948	1.08e-8	0.073	C	A	-0.251	5.50	0.092	BLINK
SWT100	5_21173402	6	42482684	6.58e-7	0.057	C	A	-0.330	26.29	0.091	BLINK
SWT100	9_13495720	9	30855923	1.35e-7	0.500	C	T	-0.128	13.81	0.048	BLINK

Trait	SNP	Chr	Position (bp)	P-value	MAF	Major Allele	Minor Allele	Effect	PVE (%)	SE	Model
SWT100	9_7486354	9	36027957	2.47e-11	0.096	A	G	0.301	6.51	0.122	BLINK
SWT100	8_41767537	10	49130402	3.85e-8	0.185	C	T	0.193	1.35	0.062	BLINK
SWT100	8_43567722	10	50930579	1.63e-7	0.099	G	T	-0.271	12.86	0.113	BLINK
SWT100	2_14753655	1	24157941	1.49e-5	0.201	G	C	-0.135	31.98	0.050	FarmCPU
SWT100	6_35097503	3	2387273	3.55e-6	0.417	C	G	0.112	3.84	0.050	FarmCPU
SWT100	5_19686583	6	36281948	1.35e-6	0.073	C	A	-0.194	5.50	0.092	FarmCPU
SWT100	5_21173402	6	42482684	3.58e-7	0.057	C	A	-0.361	26.29	0.091	FarmCPU
SWT100	7_14996355	8	31381571	1.39e-5	0.088	G	T	-0.179	5.43	0.100	FarmCPU
SWT100	9_5070328	9	6080393	3.29e-6	0.299	C	T	0.108	0.36	0.054	FarmCPU
SWT100	9_13495720	9	30855923	1.25e-6	0.500	C	T	-0.110	13.81	0.048	FarmCPU
SWT100	8_43567722	10	50930579	1.51e-5	0.099	G	T	-0.212	12.86	0.113	FarmCPU
SWT100	2_14494712	1	24413902	5.03e-6	0.236	G	A	-0.233	31.62	0.052	MLM
SWT100	5_35265704	4	7227756	6.60e-8	0.098	T	C	-0.386	29.17	0.069	MLM
SWT100	5_21173402	6	42482684	8.26e-9	0.057	C	A	-0.544	26.29	0.091	MLM
SWT100	2_14494712	1	24413902	1.00e+0	0.201	G	A	-1.306	31.50	0.101	SVEN
SWT100	6_36577242	3	267038	8.80e-1	0.182	T	C	0.722	8.96	0.120	SVEN
SWT100	5_25707455	4	26422272	8.13e-1	0.094	T	G	-1.171	13.89	0.152	SVEN
SWT100	1_5741121	5	44081345	9.97e-1	0.189	T	C	-1.050	9.34	0.171	SVEN
SWT100	5_21173402	6	42482684	1.00e+0	0.057	C	A	-2.081	26.29	0.182	SVEN
SWT100	4_15777525	7	50220517	9.25e-1	0.472	C	G	-0.933	24.74	0.085	SVEN
SWT100	7_22470394	8	53232135	8.14e-1	0.208	T	C	0.703	9.16	0.116	SVEN
SWT100	scaffold_387_2663	9	28739080	5.49e-1	0.064	G	A	0.071	0.03	0.200	SVEN
SWT100	9_10797185	9	32066020	9.39e-1	0.473	G	C	0.662	12.46	0.092	SVEN
SWT100	scaffold_273_77916	10	37502339	7.28e-1	0.062	T	C	-0.990	6.72	0.193	SVEN
SWT100	8_36270211	10	43643353	9.99e-1	0.340	T	C	-1.175	36.46	0.081	SVEN
Seed_area_mm2	7_53591467	8	70674432	1.35e-5	0.120	C	G	0.277	0.01	0.132	BLINK

MAF = minor allele frequency; PVE = phenotypic variance explained (naive single-marker R^2); SE = standard error of the marker effect estimate.

Table S7. Nonparametric bootstrap confidence intervals for PVE at low-MAF, high-PVE loci (Beavis effect assessment).

SNP	Chr	Position (bp)	Trait	Major/ Minor	MAF	Model(s)	PVE (point est.)	95% CI (bootstrap)	N
5_21173402	6	42482684	100-Seed Weight	C/A	5.7%	BLINK, FarmCPU, MLM, SVEN	26.29%	15.85–37.08%	368
5_35265704	4	7227756	100-Seed Weight	T/C	9.8%	MLM	29.12%	18.41–40.53%	368
scaffold_386_126047	7	40129332	Seed Area	C/T	7.5%	BLINK, FarmCPU	27.59%	18.30–37.83%	368
scaffold_386_126047	7	40129332	Seed Length	C/T	7.5%	BLINK, FarmCPU, SVEN	25.86%	16.30–35.76%	368

PVE = phenotypic variance explained (naive single-marker R^2). MAF = minor allele frequency. N = number of genotype-phenotype pairs used per bootstrap. Model(s) indicates which GWAS model(s) (BLINK, FarmCPU, MLM, SVEN) identified the association. scaffold_386_126047 is associated with both Seed Area and Seed Length, consistent with a pleiotropic or linked locus affecting overall seed size.

Table S8. Genomic prediction accuracy and selection efficiency of seed traits under random 10-fold cross-validation (100 cycles).

Accuracies are Pearson correlations (r) between GEBVs and observed BLUEs. CI = top-20 selection coincidence index (% overlap between top-20 GEBV and top-20 BLUE rankings).

Trait	gBLUP					gBLUP + GWAS SNPs					Change (SNPs − gBLUP)		
	Mean (r)	SD	Min	Max	CI (%)	Mean (r)	SD	Min	Max	CI (%)	Δ Mean (r)	Δ Mean (%)	Δ CI (pp)
100-Seed Weight	0.844	0.056	0.581	0.952	70	0.855	0.051	0.609	0.960	80	0.011	1.33	10
Seed Width	0.835	0.053	0.632	0.951	70	0.841	0.054	0.601	0.967	70	0.007	0.79	0
Seed Area	0.817	0.058	0.592	0.943	80	0.824	0.058	0.585	0.934	60	0.006	0.80	-20
Seed Perimeter	0.800	0.067	0.531	0.936	80	0.804	0.067	0.520	0.940	60	0.005	0.56	-20
Seed Length	0.758	0.094	0.322	0.919	80	0.772	0.088	0.227	0.914	60	0.014	1.78	-20
Aspect Ratio	0.461	0.134	-0.068	0.808	60	0.479	0.131	0.058	0.806	40	0.017	3.73	-20
Seed Circularity	0.322	0.164	-0.294	0.815	40	0.324	0.139	-0.315	0.779	20	0.002	0.62	-20

Table S9. Genomic prediction accuracy and selection efficiency under leave-one-cluster-out (LOCO) cross-validation across K = 4 PC-based subpopulations.

Trait	Prediction Accuracy by Cluster (Pearson r)				Summary				Selection
	Cluster 1	Cluster 2	Cluster 3	Cluster 4	Mean (r)	SD	Min	Max	
100-Seed Weight	0.282	0.458	**0.674**	0.251	0.416	0.195	0.251	0.674	20% / -50.7%
Seed Width	0.376	0.467	**0.695**	0.286	0.456	0.176	0.286	0.695	25% / -45.4%
Seed Area	0.364	0.351	**0.616**	0.234	0.391	0.161	0.234	0.616	20% / -52.1%
Seed Perimeter	0.260	0.320	**0.527**	0.193	0.325	0.144	0.193	0.527	20% / -59.4%
Seed Length	0.220	0.247	**0.396**	0.139	0.251	0.107	0.139	0.396	10% / -67.0%
Aspect Ratio	0.292	**0.391**	0.294	0.230	0.302	0.067	0.230	0.391	0% / -34.6%
Seed Circularity	-0.002	**0.176**	0.160	0.075	0.102	0.082	-0.002	0.176	5% / -68.2%

Selection column reports the top-20 coincidence index (%) followed by the relative drop in mean prediction accuracy versus random 10-fold cross-validation (%).
